# Conducting polymer hydrogels based on supramolecular strategies for wearable sensors

**DOI:** 10.1002/EXP.20220167

**Published:** 2024-03-14

**Authors:** Zhiyuan Sun, Qingdong Ou, Chao Dong, Jinsheng Zhou, Huiyuan Hu, Chong Li, Zhandong Huang

**Affiliations:** ^1^ School of Chemical Engineering and Technology Xi'an Jiaotong University Xi'an People's Republic of China; ^2^ Macao Institute of Materials Science and Engineering (MIMSE) Faculty of Innovation Engineering Macau University of Science and Technology Macao Taipa People's Republic of China; ^3^ Chemistry and Physics Department College of Art and Science The University of Texas of Permian Basin Odessa Texas USA; ^4^ College of Chemistry and Environmental Engineering Shenzhen University Shenzhen People's Republic of China; ^5^ Guangdong Polytechnic of Science and Technology Zhuhai People's Republic of China

**Keywords:** conductive polymer, hydrogel, supramolecular interaction, wearable sensor

## Abstract

Conductive polymer hydrogels (CPHs) are gaining considerable attention in developing wearable electronics due to their unique combination of high conductivity and softness. However, in the absence of interactions, the incompatibility between hydrophobic conductive polymers (CPs) and hydrophilic polymer networks gives rise to inadequate bonding between CPs and hydrogel matrices, thereby significantly impairing the mechanical and electrical properties of CPHs and constraining their utility in wearable electronic sensors. Therefore, to endow CPHs with good performance, it is necessary to ensure a stable and robust combination between the hydrogel network and CPs. Encouragingly, recent research has demonstrated that incorporating supramolecular interactions into CPHs enhances the polymer network interaction, improving overall CPH performance. However, a comprehensive review focusing on supramolecular CPH (SCPH) for wearable sensing applications is currently lacking. This review provides a summary of the typical supramolecular strategies employed in the development of high‐performance CPHs and elucidates the properties of SCPHs that are closely associated with wearable sensors. Moreover, the review discusses the fabrication methods and classification of SCPH sensors, while also exploring the latest application scenarios for SCPH wearable sensors. Finally, it discusses the challenges of SCPH sensors and offers suggestions for future advancements.

## Graphical abstract

This review summarizes typical supramolecular strategies for developing high‐performance conducting polymer hydrogels (CPHs) and elucidates their properties relevant to wearable sensors. Additionally, it discusses the fabrication methods and classification of supramolecular CPH sensors and their latest applications in wearable devices. Finally, the review addresses these sensors’ challenges and provides suggestions for future developments.

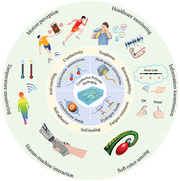

## 1 INTRODUCTION

In the Internet of Things era, wearable sensors have emerged as a focal point, capturing widespread attention and undergoing rapid development.^[^ ^1^, ^2^, ^3^, ^4^, ^5^, ^6^, ^7^, ^8^ ^]^ As lightweight and multifunctional devices, wearable sensors establish close connections with the human body and possess the ability to sense, record, and transmit a wide range of crucial data. From smartwatches^[^ ^9^, ^10^ ^]^ to health trackers^[^ ^11^, ^12^ ^]^ and smart glasses,^[^ ^13^, ^14^ ^]^ wearable sensors are finding applications in a wide range of fields, revolutionizing our lifestyles and professional environments. In order to cater to the demands of emerging applications, wearable sensors must possess excellent electrical conductivity and mechanical flexibility. At present, conventional sensors are primarily developed by incorporating conductive fillers, such as metallic nanomaterials,^[^ ^15^, ^16^ ^]^ liquid metals,^[^ ^17^, ^18^ ^]^ and carbon‐based materials,^[^ ^19^, ^20^ ^]^ into flexible substrates. However, the inherent rigidity and unfavorable dispersion of conductive fillers lead to the sensors’ restricted stretchability and subpar conductivity, limiting their application in complex stress environments. Hence, pursuing inherently stretchable conductive materials has emerged as a significant and ongoing challenge in wearable sensors.

To address this issue, researchers have made significant strides in developing a novel class of conductive soft materials known as conductive polymer hydrogel (CPH).^[^ ^21^, ^22^ ^]^ By combining the advantageous properties of conductive polymer (CP) and hydrogel, these materials exhibit exceptional conductivity and highly adjustable mechanical characteristics. As an intrinsically stretchable and conductive material, CPH emerges as a novel platform for wearable sensors, offering enhanced reliability, comfort, and precise signal monitoring.^[^ ^23^, ^24^, ^25^, ^26^ ^]^ Among the various CPs utilized in the fabrication of CPHs, polyaniline (PANI),^[^ ^27^, ^28^ ^]^ polypyrrole (PPy),^[^ ^29^, ^30^ ^]^ and poly(3,4‐ethylene‐dioxythiophene) (PEDOT)^[^ ^31^, ^32^, ^33^ ^]^ are the most commonly employed due to their tunable electrical properties and straightforward synthesis methods. These CPs possess a conjugated structure that facilitates the retention and transfer of electrons within their backbones.^[^ ^34^, ^35^ ^]^ Consequently, the incorporation of CPs establishes a highly continuous electron transport pathway within hydrogels, granting CPHs exceptional electronic conductivity. This characteristic is the foundation for sensors to achieve high‐precision acquisition and transmission of electrical signals. Moreover, CPHs possess the pliable characteristics of hydrogels, thereby minimizing mechanical discrepancies with biological tissues, making them highly suitable for wearable electronic devices. At present, wearable sensors founded on CPHs are widely employed in motion monitoring,^[^ ^36^, ^37^ ^]^ healthcare,^[^ ^38^, ^39^ ^]^ human–computer interaction systems,^[^ ^40^, ^41^ ^]^ and other domains associated with human activities.^[^ ^42^, ^43^ ^]^

Although considerable progress has been achieved in the field of wearable sensors by CPHs, numerous formidable challenges still require attention. One of these challenges is the thermodynamic incompatibility between hydrophobic CPs and hydrophilic hydrogel networks, resulting in a weak combination between the two components.^[^ ^28^, ^44^ ^]^ The weak bonding hampers the effective dispersion of CPs within the hydrogel network, making them prone to agglomeration. Consequently, this agglomeration gives rise to the formation of fragmented conductive pathways and the emergence of stress‐defective regions, ultimately compromising the conductivity and mechanical integrity of CPHs. Currently, in most reported CPHs, CPs are incorporated into hydrogel networks through physical entanglement in the form of interpenetrating networks (IPNs). However, the absence of efficient interaction between CP and hydrophilic polymers in these IPN‐based CPHs results in CP aggregation and subsequent phase separation. This aggregation phenomenon further leads to the degradation of CPH properties. On the one hand, the continuous conductive paths in the CPH network are disrupted, significantly reducing its electrical conductivity. On the other hand, agglomerated CPs in CPHs cause stress concentration, leading to a reduction in their ability to withstand stress. Furthermore, with the continuous advancement of technology, wearable sensors are being widely applied in daily life and healthcare, with increasingly complex scenarios. Sensors have more functional requirements for materials, including sensitivity, reliability, biocompatibility, and lifespan. Therefore, it is imperative to find effective strategies to establish robust interactions within CPH, thus developing high‐performance and multifunctional CPH to meet the demands of wearable sensors.

In the crosslinked network of CPH, in addition to covalent bonds, there exist non‐covalent interactions among the polymerized networks. These non‐covalent interactions, also called supramolecular interactions, include hydrogen bonding, metal coordination, host‐guest interactions, *π–π* stacking, hydrophobic interactions, and van der Waals interactions.^[^ ^45^, ^46^, ^47^ ^]^ By incorporating supramolecular interactions into CPH, the bonding between CP and the hydrogel network is enhanced, forming a stable gel structure. In addition, the physicochemical properties and functions of hydrogels, such as self‐healing, self‐adhesion, anti‐swelling, and fatigue resistance, are modified by tuning the type, strength and abundance of supramolecular interactions. Introducing these properties equips wearable sensors with more functions, further improving the sensors’ sensitivity, stability, and reliability. Therefore, supramolecular interactions are important for developing high‐performance and multifunctional sensors. Through a comprehensive understanding of the principles of supramolecular interactions, precise control of the structure and properties of hydrogels can be achieved, thus providing new perspectives and methods for the innovation and utilization of CPH sensors.

**FIGURE 1:**
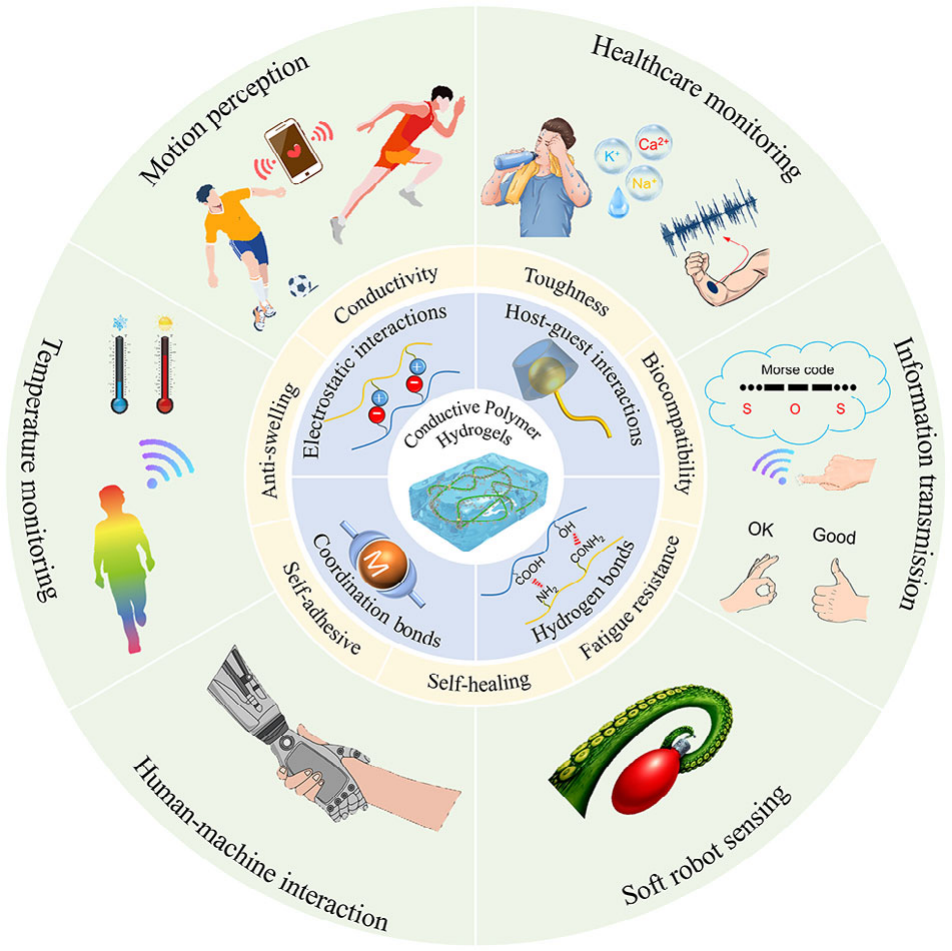
Introduce the common supramolecular strategies for preparing SCPHs, the characteristics of SCPHs, and their related applications in wearable sensing.

So far, several preliminary reviews have been reported on CPH due to their increasing prominence.^[^ ^48^, ^49^, ^50^ ^]^ However, more comprehensive summary and understanding are urgently required to thoroughly explore the interaction forces present in CPH from the perspective of supramolecular chemistry. This review aims to systematically summarize the various strategies employed in CPH. The objective is to gain a comprehensive understanding of the interactions in CPH through the lens of supramolecular chemistry, providing valuable theoretical guidance for developing high‐performance and multifunctional CPH, particularly for their application in wearable sensors (Figure 1):

1. This review provides an in‐depth summary of several representative supramolecular strategies commonly employed in developing CPHs in recent years. These strategies include hydrogen bonds, electrostatic interactions, host‐guest interactions, and coordination bonds.
2. The review comprehensively discusses the key sensor‐related properties of supramolecular CPHs (SCPHs), such as conductivity, mechanical toughness, biocompatibility, self‐healing, self‐adhesion, and resistance to swelling.
3. This review summarizes the common preparation methods and classifications of SCPHs, shedding light on their distinctive implementation in wearable sensors. These scenarios encompass motion perception, sweat analysis, temperature sensing, electrophysiological signal monitoring, human–machine interaction, soft robot sensing, and information transmission.
4. This review addresses the current challenges and future perspectives in the field, particularly proposing directions for further development of SCPHs for wearable sensor applications.

## 2 DESIGN OF CPHS BASED ON SUPRAMOLECULAR INTERACTIONS

CPH is a remarkable composite material that combines the advantageous properties of conductive polymers and hydrogels.^[^ ^31^, ^51^ ^]^ It is synthesized by incorporating CP into a hydrophilic hydrogel network. However, it is crucial to emphasize that when CP is introduced into the hydrogel network without effective interactions, it merely becomes embedded within the hydrogel structure as an interpenetrating network. This means insufficient interaction between CP and the hydrophilic polymer network, resulting in CP aggregation and consequent performance degradation. To address this issue, it becomes imperative to introduce additional interaction forces into the CPH structure, thereby enhancing the overall performance of CPs.

Supramolecular interaction forces refer to those that arise between molecules through non‐covalent bonds. While non‐covalent bonds are relatively weak compared to covalent bonds, they play a crucial role in regulating material properties. Incorporating non‐covalent bonds into covalently crosslinked CPHs, can harness additional interaction forces, resulting in the emergence of new properties and functionalities in these materials. For instance, introducing dynamic non‐covalent bonds imparts reversibility to CPHs, enabling the network to reorganize and achieve self‐repair dynamically in the event of damage. Additionally, the incorporation of non‐covalent bonds enhances the controllability and selectivity of CPHs. By carefully regulating the type and strength of these bonds, the physical and chemical properties of hydrogel can be precisely tailored, facilitating the design and realization of specific functions.

**FIGURE 2:**
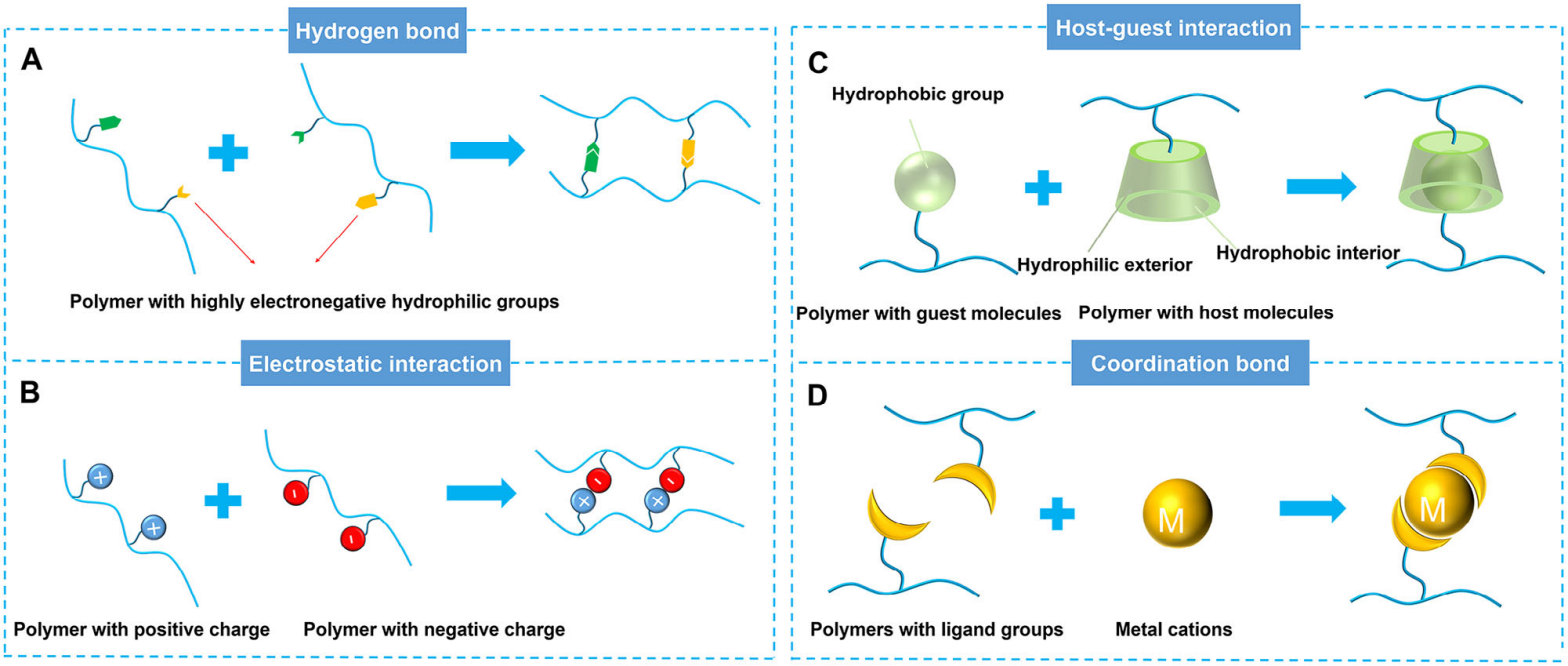
The mechanism diagram of supramolecular interactions. (A) Hydrogen bond. (B) Electrostatic interactions. (C) Host‐guest interactions. (D) Coordination bond.

Several common supramolecular strategies employed in preparing CPHs include hydrogen bonding, electrostatic interactions, host‐guest interactions, and Coordination bonding. Hydrogen bonding, a prevalent supramolecular interaction mode, involves the interaction between hydrogen atoms and atoms with high electronegativity. Given numerous hydrophilic groups with high electronegativity, hydrogen bonding readily occurs within the polymer network of CPHs (Figure 2A). By introducing functional groups with hydrogen bond donors and acceptors, stable hydrogen bonding can be established within the hydrogel network. Electrostatic interactions arise from the mutual attraction between positively and negatively charged components, which can be achieved by incorporating positively and negatively charged components into the CPH structure (Figure 2B). Host‐guest interactions rely on the interactions between host and guest molecules. By introducing host molecules with specific functional groups, selective recognition and immobilization of guest molecules can be achieved, thereby regulating the structure and properties of CPHs (Figure 2C). Coordination bonding involves the coordination interaction between metal ions and ligands. Stable ligand bonds can be formed by introducing molecules containing ligand groups into the CPHs network (Figure 2D).

**FIGURE 3:**
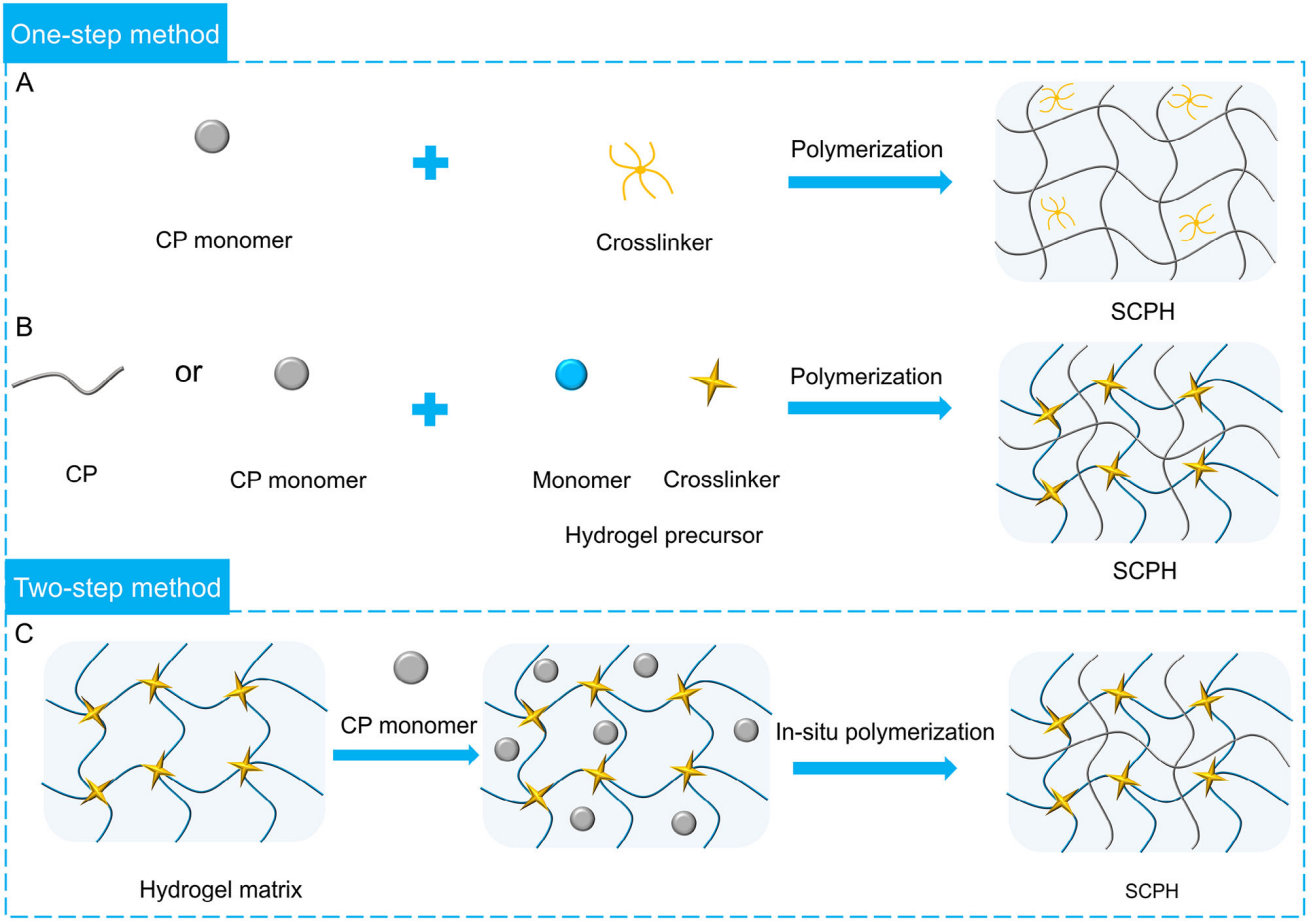
Schematic representation of commonly used preparation techniques for SCPHs. (A) SCPHs are obtained through the direct crosslinking of CP with crosslinking agents. (B) SCPHs are synthesized via a one‐step polymerization process, wherein CP monomer and a hydrogel precursor liquid are blended and polymerized to yield SCPHs. (C) SCPHs are synthesized by utilizing a pre‐prepared hydrogel matrix as a template, wherein CP monomer is initially introduced and subsequently polymerized in situ within the hydrogel matrix to generate SCPHs.

Common preparation methods for SCPHs can be categorized into two main types: one‐step and two‐step methods. In the one‐step method, a crosslinked structure is formed by initiating the polymerization of CP monomers in the presence of a crosslinking agent (Figure 3A). Since most conductive polymers contain positively charged groups, introducing substances with opposite charges can crosslink the polymer chains of CP through electrostatic interactions. Examples of such negatively charged crosslinking agents include phytic acid,^[^ ^52^, ^53^, ^54^ ^]^ amino trimethylene phosphonic acid,^[^ ^55^, ^56^ ^]^ sodium dodecyl benzene sulfonate,^[^ ^54^ ^]^ and sulfonated multi‐walled carbon nanotubes.^[^ ^57^ ^]^ Additionally, commercially available conductive polymer PEDOT: PSS can also be used as a crosslinking agent to crosslink other conductive polymers like PANI and PPy. Another method involves the one‐pot polymerization of CP monomers or CP mixed with a hydrogel precursor liquid (Figure 3B). The hydrogel precursor liquid contains a hydrophilic monomer, a crosslinking agent, and an initiator, while the conductive component can be CP or a CP monomer. In this method, the CP or CP monomer is dispersed into the hydrogel precursor liquid, and the polymerization is initiated to form SCPHs. The one‐pot method offers a simple preparation process and easy operation. The properties of SCPHs can be regulated by adjusting the ratio of CP or CP monomer to the hydrogel precursor liquid, the conditions of the polymerization reaction, and other factors.

In the two‐step method, the primary hydrogel matrix is first obtained through free radical polymerization. Then, the CP monomer is introduced into the hydrogel matrix through adsorption, followed by in situ polymerization to form the CP network within the hydrogel matrix (Figure 3C). This method allows for the design and regulation of the hydrogel primary network, and specific supramolecular chemical bonds can be pre‐introduced into the hydrogel network. During the in situ polymerization reaction, the CP utilizes the hydrogel network as a template and interacts with it through supramolecular forces, forming a uniform and stable conductive network.

Here, we summarize several common supramolecular strategies for preparing SCPHs, discussing and analyzing the design and construction of these hydrogels. Through the rational selection of supramolecular strategies and optimization of hydrogel structures, precise control over the properties of SCPHs can be achieved, offering new ideas and methods for their applications in electronic devices, sensors, biomedicine, and energy storage.

### 2.1 Hydrogen bonds

The hydrogen bond is a widely recognized and dominant interaction in supramolecular chemistry. It embodies a directed electronic interaction between a donor and an acceptor molecule. Hydrogen bonds are weak interaction forces with strengths in the range of 10–65 kJ mol^−1^, which are typically weaker than covalent and electrostatic interactions but stronger than van der Waals forces.^[^ ^45^ ^]^ Notably, the strength of hydrogen bonds can be adjusted by manipulating the solvent or the number and arrangement of hydrogen bond donors and acceptors. Furthermore, hydrogen bonds exhibit dynamic reversibility, undergoing reversible fracture and reorganization in response to external stimuli.^[^ ^58^ ^]^ By leveraging these properties, hydrogen bonds have found extensive applications in the design of SCPHs, offering enhanced mechanical properties, improved processability, and self‐healing capabilities.

**FIGURE 4:**
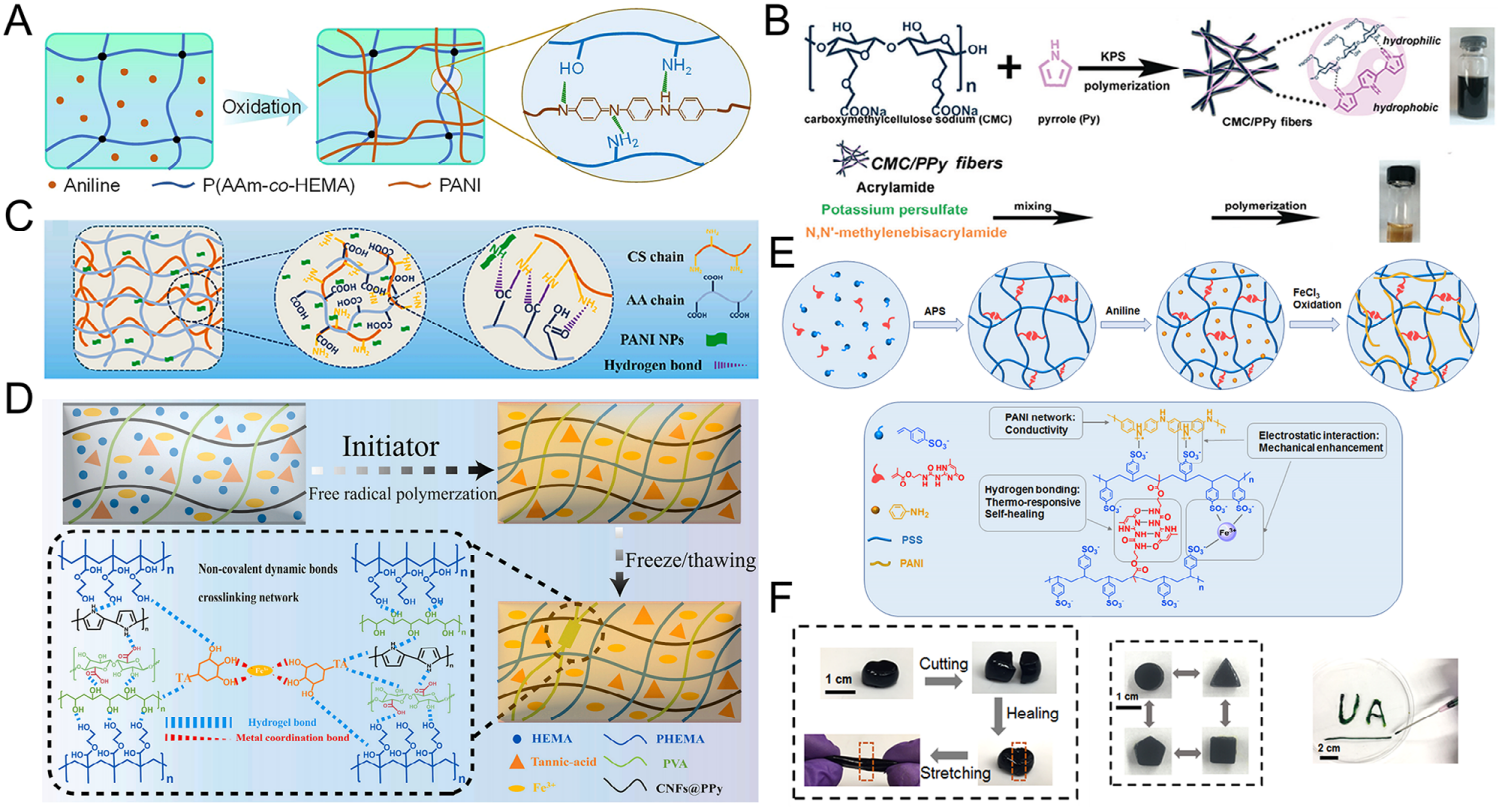
Construction of SCPHs based on hydrogen bonds. (A) Preparation of P(AAm‐*co*‐HEMA)/PANI interpenetrating network hydrogels. Reproduced with permission.^[^ ^59^ ^]^ Copyright 2018, American Chemical Society. (B) The preparation process of PAAm/CMC/PPy hydrogel. Reproduced with permission. Reproduced with permission.^[^ ^60^ ^]^ Copyright 2020, Royal Society of Chemistry. (C) Preparation and schematic illustration of the structure of PANI NPs@(CS‐PAA) hydrogels.^[^ ^61^ ^]^ Copyright 2023, Elsevier. (D) Schematic representation of the synthesis mechanism of PHEMA/PVA/CNFs@PPy hydrogels and the interactions between the polymer networks. Reproduced with permission.^[^ ^62^ ^]^ Copyright 2023, Elsevier. (E) Schematic of the formation mechanism of supramolecular PANI/PSS‐UPy hydrogels, and (F) their self‐healing, freely moldable, and injectable properties. Reproduced with permission.^[^ ^63^ ^]^ Copyright 2019, American Chemical Society.

The hydrogel network composed of hydrophilic polymers is abundant in hydrophilic functional groups, including hydroxyl, amine, carboxyl, and amide, which can serve as acceptors and donors of hydrogen bonds. Consequently, the integration of hydrogen bonds into SCPHs can be achieved through a reasonable design of the hydrophilic polymer network. For example, Fu et al. prepared an interpenetrating network hydrogel based on hydrogen bonding by in situ polymerization of polyaniline (PANI) in a copolymer of acrylamide and hydroxyethyl acrylate.^[^ ^59^ ^]^ The aniline groups on PANI and the amide and hydroxyl groups on the side chains of poly(acrylamide‐*co*‐hydroxyethyl acrylate) formed a strong bond through hydrogen bonding (Figure 4A). The hydrogels exhibit excellent strength, toughness, and a linear resistance change upon stretching due to the reliable interactions between the conductive and flexible polymers.

Natural polymers with many hydrophilic groups in their structure can serve as a bridge between CPs and hydrophilic polymers. They have a good affinity for CPs, and can effectively bind to hydrophilic polymers through hydrogen bonding. As a result, incorporating natural polymers into SCPHs substantially enhances the compatibility between hydrophobic CPs and hydrophilic polymer networks. For instance, Gao et al. proposed the utilization of sodium carboxymethyl cellulose (CMC) as a stabilizer for hydrophobic polypyrrole (PPy) to fabricate high‐performance SCPHs (Figure 4B).^[^ ^60^ ^]^ The incorporation of CMC enhanced the hydrophilicity of PPy, owing to its remarkable emulsifying capability. Simultaneously, PPy spontaneously assembled into fibrous structures with CMC through hydrophobic interactions, hydrogen bonding, and electrostatic interactions. The incorporation of CMC/PPy fibers into PAM hydrogels resulted in the development of SCPHs exhibiting remarkable conductivity, transparency, and stretchability. The exceptional characteristics of SCPHs were attributed to the homogeneous dispersion of CMC/PPy within the hydrophilic PAM network facilitated by various physical interactions. Similarly, Gu et al. obtained SCPHs with enhanced mechanical and electrical conductivity properties by in situ generation of PANI in chitosan‐polyacrylic acid (CS‐PAA) hydrogel matrix (Figure 4C).^[^ ^61^ ^]^ The PANI structure comprises a hydrophilic imide group, which establishes a hydrogen bond with the amide group of CS‐PAA. Meanwhile, the structurally hydrophobic benzene ring forms a hydrophobic bond through π‐π conjugation. The synergistic effect of these two non‐covalent interactions was pivotal in augmenting the properties of the hydrogel. Particularly, the hydrogen bonds contributed to the amplification of physical crosslinking points within the hydrogel network, thereby conferring exceptional toughness and stretchability to the hydrogel. Likewise, Wang and colleagues fabricated versatile SCPHs through the integration of PPy‐doped modified cellulose nanofibers, poly(vinyl alcohol), and poly(2‐hydroxyethyl methacrylate) (Figure 4D).^[^ ^62^ ^]^ Strong connections between all the polymer networks were established through hydrogen bonding, resulting in SCPHs with excellent mechanical properties, rapid self‐repairing properties, and strong adhesion.

Interestingly, the number and strength of hydrogen bonds are tunable. While single hydrogen bond is relatively weak, multiple hydrogen bonds with strong interactions can significantly modulate the properties of hydrogels and achieve multifunctionality. For example, 2‐ureido‐4[1H] pyrimidinone (UPy), a group containing multiple hydrogen bonds, is dynamically reversible. Zeng et al. prepared a multifunctional SCPH by incorporating multiple hydrogen‐bonded UPy groups as crosslinking points into a PANI/poly(4‐styrenesulfonate) network (Figure 4E).^[^ ^63^ ^]^ The dynamic and reversible dissociation/conjugation of multiple hydrogen bonds promoted network reconstruction of damaged hydrogels for rapid self‐healing. Moreover, quadruple hydrogen bonding groups with thermo‐responsive kinetics conferred thermoplasticity and fluidity to the SCPHs, making them freely moldable and injectable. (Figure 4F).

Hydrogen bonds are characterized by high strength, orientation, and dynamic reversibility. These properties allow for precise control over the structure and properties of hydrogels, enabling the realization of diverse functions. The incorporation of hydrogen bonding not only enhances the structural stability of SCPHs but also offers supplementary physical crosslinking sites, thereby enhancing the mechanical properties of SCPHs. Moreover, the dynamic reversibility of hydrogen bonding provides the self‐healing properties of hydrogel materials, enabling them to repair themselves after damage and extending the material's service life. Additionally, hydrogen bonding can be employed to construct SCPHs with specific shapes and pore structures to meet the needs of different fields. In conclusion, hydrogen bonding provides a flexible and controllable material design strategy, which offers new possibilities for scientific research and engineering applications of SCPHs in various fields.

### 2.2 Electrostatic interactions

Electrostatic interactions involve the bonding of molecules with opposite charges through electrostatic attraction. These electrostatic interactions can exert strong bonding forces during the formation of hydrogels. By incorporating oppositely charged groups, the introduction of electrostatic interactions into hydrogels becomes feasible, forming tight bonds between polymer chains and resulting in a stable hydrogel structure. Given that most conductive polymers contain positively charged groups within their polymer chains, the linkage between hydrophobic conductive polymers and hydrophilic materials can be effectively achieved by introducing negatively charged groups to the hydrophilic materials. Notably, the utilization of electrostatic interactions in the preparation of SCPHs has emerged as a rapidly advancing research field.

**FIGURE 5:**
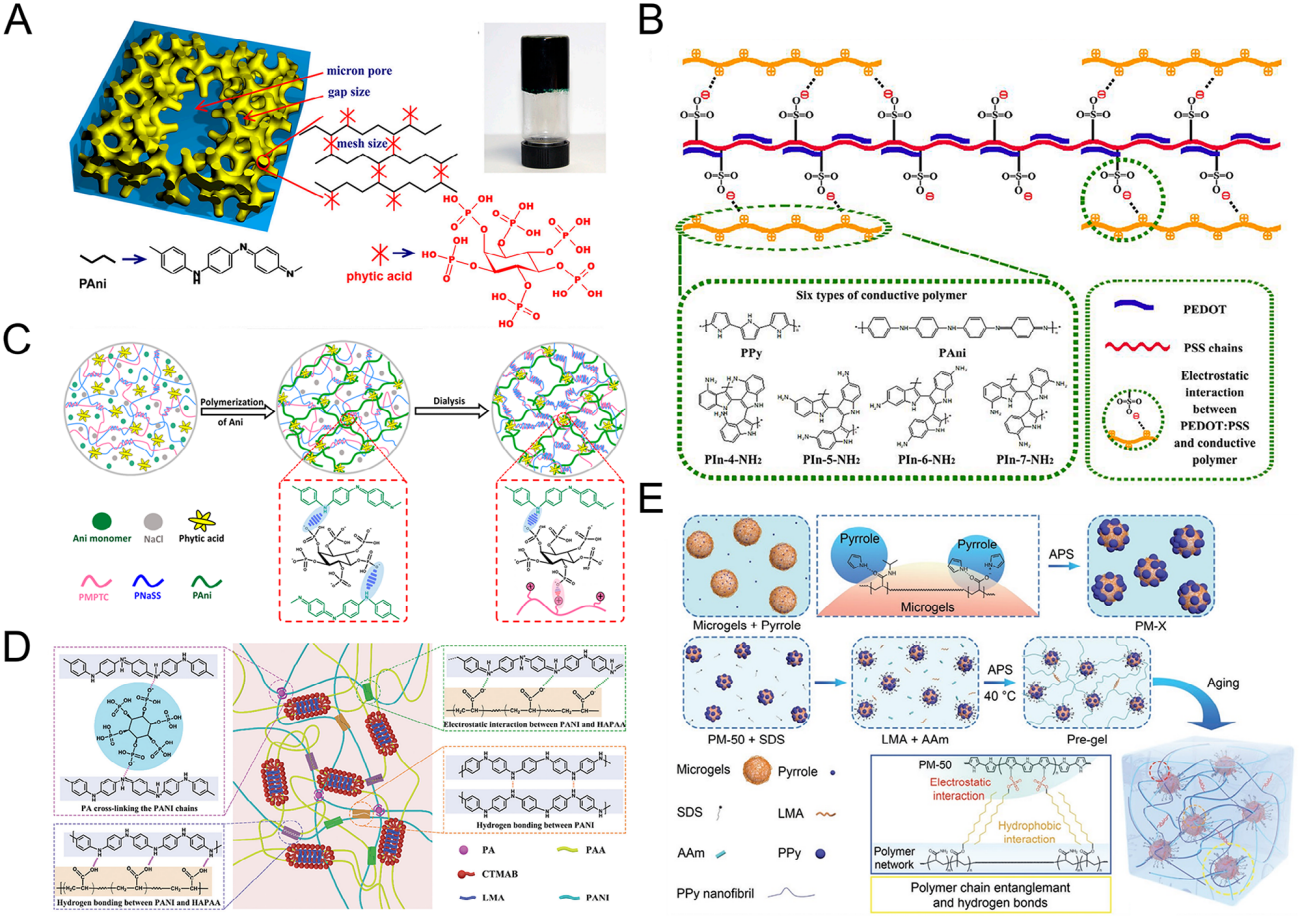
The SCPHs are prepared by electrostatic interactions. (A) The gelation process of PA crosslinked PANI. Reproduced with permission.^[^ ^52^ ^]^ Copyright 2012, National Academy of Sciences. (B) Schematic diagram of the gelation process of PEDOT: PSS as a crosslinker for crosslinking six conductive polymers. Reproduced with permission.^[^ ^64^ ^]^ Copyright 2022, Elsevier. (C) Mechanism diagram of the formation of PIC/PANI composite hydrogel, where PANI is crosslinked to the PIC matrix by PA. Reproduced with permission.^[^ ^65^ ^]^ Copyright 2018, American Chemical Society. (D) Schematic structure of HAPAA/PANI hydrogel and multiple interactions between polymer networks. Reproduced with permission.^[^ ^66^ ^]^ Copyright 2021, Royal Society of Chemistry. (E) Schematic mechanism of synthesis of composite SCPHs loaded with PPy microgel. Reproduced with permission.^[^ ^67^ ^]^ Copyright 2023, Wiley‐VCH.

Hydrophilic materials with negatively charged structures have been proven effective in crosslinking conducting polymers through electrostatic interactions, resulting in the direct formation of 3D network‐structured SCPHs. For instance, Bao et al. pioneered a method for preparing SCPHs utilizing phytic acid (PA) as a crosslinker for polyaniline (Figure 5A).^[^ ^52^ ^]^ PA reacted with PANI by protonating the nitrogen groups on PANI, forming SCPHs with a high surface area and 3D porous nanostructures. These hydrogels showed excellent conductivity and electrochemical properties. Additionally, commercially available CP can be employed as a crosslinking agent to prepare SCPHs. For example, Wang et al. introduced the use of the commercial conductive polymer PEDOT: PSS as a conductive dopant for crosslinking a range of conductive polymers (Figure 5B).^[^ ^64^ ^]^ Among these, SCPHs were formed through electrostatic interactions between the negatively charged SO_3_^−^ groups in PEDOT: PSS and the positively charged groups on PANI, PPy, and polyaminoindole molecular chains. These SCPHs, consisting of pure conductive polymers, possessed excellent electrical conductivity.

Although SCPHs without insulating polymer networks exhibit satisfactory electrical properties, the inherent rigidity of CP limits its development for flexible sensing applications with large strain windows. Researchers have prepared stretchable SCPHs by combining flexible polymers and rigid CP to improve stretchable SCPHs for sensing and detecting large deformations. Zheng et al. utilized PA to connect PANI and polyionic polymers through electrostatic interactions (Figure 5C).^[^ ^65^ ^]^ Due to the formation of strong interactions between the rigid PANI and the flexible hydrogel matrix, the composite hydrogel displayed high electrical conductivity while maintaining viscoelasticity and toughness. Similarly, Yu et al. prepared supramolecular hydrogels HAPAA/PANI by in situ polymerization of pre‐permeabilized aniline (ANI)/phytanic acid (PA) solution in hydrophobically associative polyacrylic acid (HAPAA) hydrogel matrix (Figure 5D).^[^ ^66^ ^]^ The prepared hydrogel matrix possessed excellent stretching and good self‐healing ability due to the gradual recombination of the reversible hydrophobic micelles in HAPAA after breakage. The PANI obtained by the in‐situ polymerization formed a crosslinked supramolecular structure through electrostatic interactions of PA. Moreover, the abundant‐NH on the PANI chains and ‐COOH groups on the HAPAA chains formed intermolecular hydrogen bonds and electrostatic interactions, enabling the conductive PANI to be uniformly dispersed in the hydrogel matrix. These dynamic interfacial interactions could serve as an energy dissipation mechanism to effectively enhance the mechanical properties of HAPAA/PANI hydrogels.

However, the compatibility between highly hydrophobic conductive polymers and hydrophilic hydrogel matrices needs to be improved, resulting in substantial impairment to the mechanical properties of the hydrogel upon the introduction of excessive quantities of aggregating chlorinated paraffin. To address this issue, Zhang et al. proposed a novel approach for incorporating PPy‐containing microgels into copolymers of acrylamide and lauryl methacrylate (Figure 5E).^[^ ^67^ ^]^ The connection between the PPy‐loaded microgel and the hydrogel network was established through the interaction between sodium dodecyl sulfate (SDS) and the polymer chains. Specifically, the PPy on the microgel bound to the sulfate groups of SDS via electrostatic interactions. At the same time, the hydrophobic units in the hydrogel structure formed a strong connection with the dodecyl groups of SDS through hydrophobic linkages. By utilizing microgels as carriers for PPy within the hydrogel matrix, this method not only enhances the stability of PPy, but also preserves the mechanical properties of the hydrogel by introducing CPs without compromising its integrity.

In conclusion, electrostatic interactions play a pivotal role in the fabrication of SCPHs. An effective binding between the highly hydrophobic conducting polymer and the hydrophilic hydrogel matrix was realized by introducing electrostatic interactions, forming a stable network structure. The electrostatic interaction effectively improves the compatibility between CP and hydrogel, thereby substantially enhancing the mechanical and electrical properties of SCPHs, which provides a solid foundation for the application of SCPHs in electronic devices.

### 2.3 Host‐guest interactions

Host‐guest interactions refer to chemical reactions or substance interactions wherein one molecule assumes the role of the primary host, while another molecule serves as the guest.^[^ ^68^, ^69^ ^]^ By interacting with guest molecules, the host molecule affects the guest molecule's properties, structure, or activity. Host molecules have higher binding strength and more specific recognition between host and guest molecules compared to other interactions. It is a highly efficient non‐covalent interaction closer to covalent interactions. Cyclodextrins, cucurbit[n]urils, and calix[n]arenes are widely employed host molecules for fabricating supramolecular hydrogels featuring tailored hydrophilic and hydrophobic architectures through polymer chain modifications.^[^ ^70^, ^71^ ^]^ These hydrogels exhibit unique properties and functionalities and find extensive applications across diverse fields.

**FIGURE 6:**
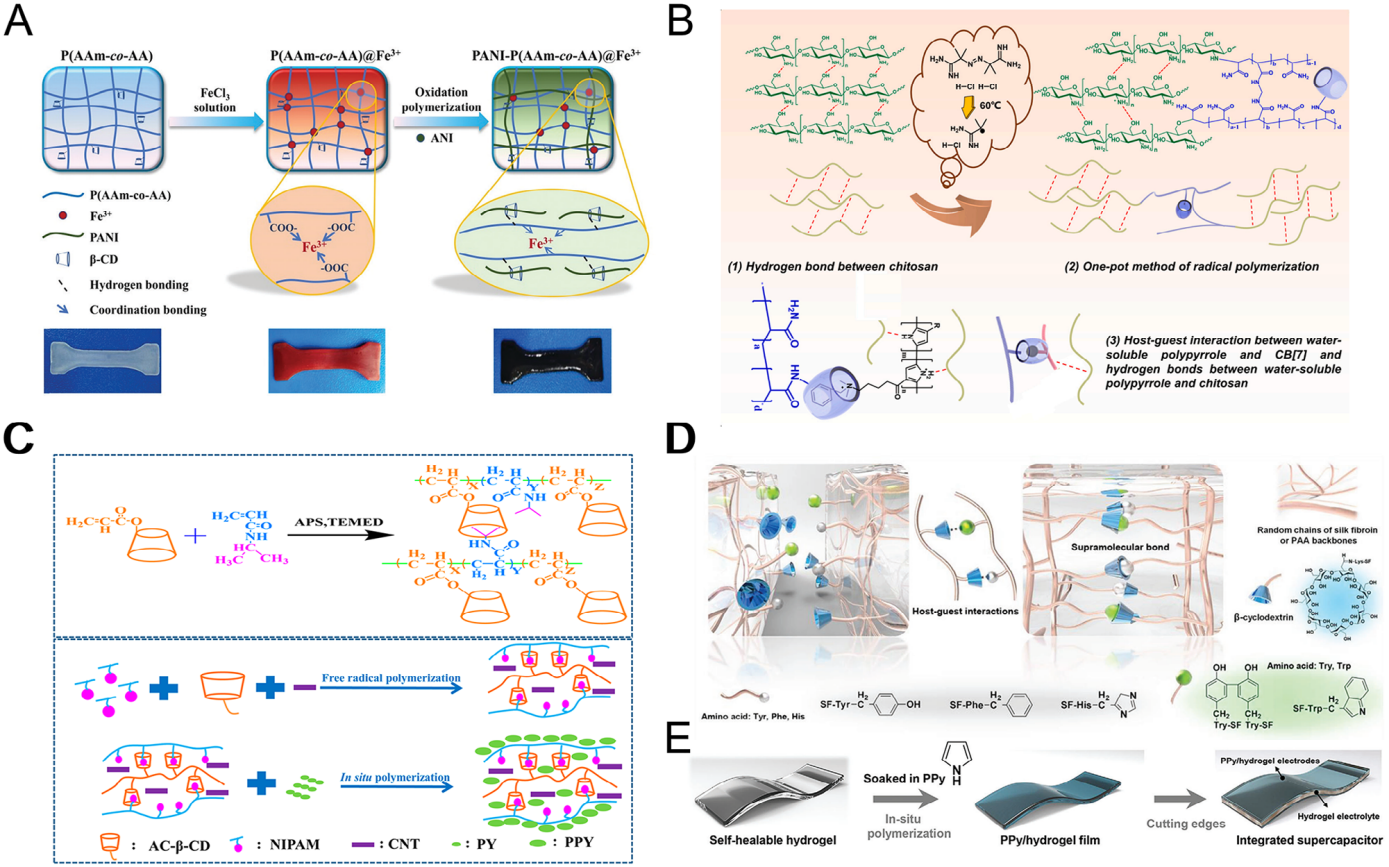
The SCPHs are prepared by the host‐guest interaction. (A) Schematic synthesis of PANI‐P(AAm‐*co*‐AA) @Fe^3+^ hydrogel. Reproduced with permission.^[^ ^74^ ^]^ Copyright 2022, Royal Society of Chemistry. (B) Schematic illustration of the preparation of CxPy hydrogels and interactions between the polymers and water‐soluble PPy. Reproduced with permission.^[^ ^76^ ^]^ Copyright 2022, Elsevier. (C) Schematic synthesis of poly (NIPAM‐co‐β‐CD). Reproduced with permission.^[^ ^77^ ^]^ Copyright 2018, American Chemical Society. (D) Preparation of supramolecular hydrogels based on host‐guest interaction and (E) their application to supercapacitor assembly. Reproduced with permission.^[^ ^78^ ^]^ Copyright 2021, Wiley‐VCH.

Cyclodextrins (CD) are macrocyclic molecules possessing an amphiphilic hollow structure with a hydrophobic inner cavity and a hydrophilic outer cavity, vital in linking hydrophilic and hydrophobic polymers.^[^ ^72^, ^73^ ^]^ Tang et al. successfully prepared dual‐network synergistic hydrogels with tunable mechanical properties and high sensitivity by introducing CD into iron ion‐crosslinked poly(acrylamide‐co‐acrylic acid) networks and conductive PANI networks (Figure 6A).^[^ ^74^ ^]^ During the reaction, the hydrophobic PANI polymerized in the cavity of the CD through host‐guest interaction. At the same time, the hydrophilic polymer is bound to the outer cavity of the CD through hydrogen bonding. The host‐guest interaction between amphiphilic CD and PANI improved the compatibility of PANI in the hydrogel matrix, effectively improving the uneven or aggregation of PANI in the hydrogel matrix.

Cucurbit[n]ureas, a novel class of macrocyclic host molecules, have garnered significant attention among scientists, owing to their remarkable affinity for a diverse range of guest molecules.^[^ ^75^ ^]^ Tan et al. presented a series of SCPHs by introducing chitosan and water‐soluble polypyrrole into cucurbit[7]uril‐modified polyacrylamide matrices (Figure 6B).^[^ ^76^ ^]^ Water‐soluble polypyrrole (PPy) was found to establish numerous hydrogen bonds with chitosan, and engage in host‐guest interactions with cucurbit[7]uril moieties in the polyacrylamide side chains. These interactions played a crucial role in augmenting the mechanical properties of the hydrogels. Furthermore, the incorporation of cucurbit[7]urea moiety not only enhanced the adhesion of the composite hydrogels, but also significantly improved their biocompatibility by binding to the benzyl quaternary ammonium salt of the water‐soluble PPy side chain.

As an efficient non‐covalent interaction, the host‐guest interaction exhibits high specific binding strength and dynamic reversibility, enabling the design of hydrogels with exceptional self‐healing efficiency. Guo's team developed a SCPH based on host‐guest interaction by introducing multi‐walled carbon nanotubes and PPy into a copolymer of vinyl‐modified β‐CD and isopropyl acrylamide (NIPAM) (Figure 6C).^[^ ^77^ ^]^ During the formation of SCPHs, the isopropyl hydrophobic aliphatic group on the structure of the guest molecule NIPAM was embedded within the cavity of the host molecule β‐CD. The prepared SCPHs exhibited excellent self‐healing ability and achieved high self‐healing efficiency, owing to the dynamic host‐guest interaction between β‐CD and NIPAM. In addition, Zhi et al. successfully synthesized a supramolecular hydrogel electrolyte by exploiting the host‐guest interactions between β‐CD grafted onto filipin proteins and poly (acrylic acid) molecular chains, as well as the neighboring amino acid molecules on filipin proteins. (Figure 6D).^[^ ^78^ ^]^ Moreover, PPy was incorporated into a supramolecular hydrogel electrolyte to create an elastic electrode material, facilitated by hydrogen bonding and covalent interactions of aromatic amino acid residues within the filament chains. By leveraging the dynamic reversibility of the host‐guest interaction, the electrode material and electrolyte formed a robust bond, showcasing remarkable self‐healing properties (Figure 6E).

The compatibility between hydrophobic CPs and the hydrogel network is significantly enhanced through host‐guest interactions. Furthermore, the interactions between the host and guest molecules are reversible, allowing the hydrogels to reassemble and self‐heal in the event of damage. In summary, through the deliberate selection and modification of host and guest molecules, it becomes feasible to fabricate SCPHs with distinctive functionalities and structures, thereby opening up new avenues for their application across diverse domains.

### 2.4 Coordination bonds

Coordination bonds represent a distinctive category of chemical bonding that forms between one or more ligands and a central metal ion.^[^ ^79^, ^80^ ^]^ Compared to other supramolecular interactions, coordination bonds exhibit a notably higher bond energy, ranging from 50 to 400 kJ mol^−1^.^[^ ^81^ ^]^ The heightened bonding energy endows coordination bonds with a pivotal role in the regulation of material properties and functionalities. By manipulating the type of coordination bonds, selecting appropriate ligands, and arranging coordination groups in specific configurations, it becomes feasible to modulate the structure and enhance the stability of materials.^[^ ^82^, ^83^ ^]^ Therefore, numerous researchers have incorporated coordination bonds into SCPHs to augment their properties and realize a wide array of functions.

**FIGURE 7:**
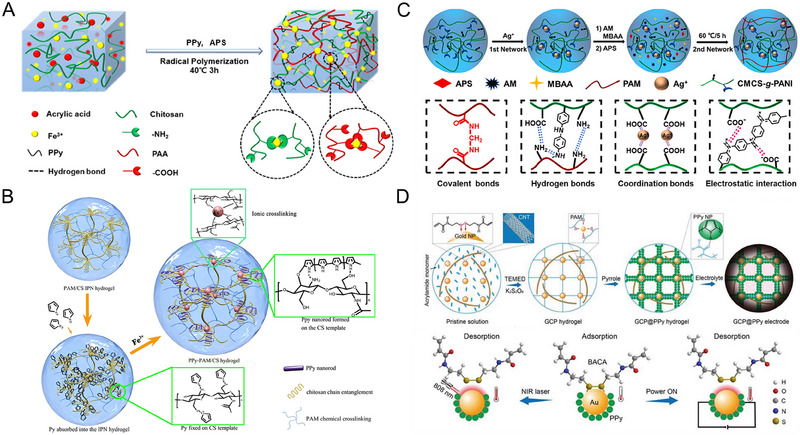
The SCPHs are fabricated by coordination bonds. (A) Diagram of the formation mechanism of CS/PAA/PPy/Fe (III) hydrogels. Reproduced with permission.^[^ ^87^ ^]^ Copyright 2021, American Chemical Society. (B) Preparation procedure and schematic of the internal structure of the PAM/CS/PPy hydrogels. Reproduced with permission.^[^ ^88^ ^]^ Copyright 2018, American Chemical Society. (C) Schematic diagram of the synthesis process of PAM/CMCS‐*g*‐PANI/Ag hydrogels and the multiple interactions within the SCPHs network. Reproduced with permission.^[^ ^89^ ^]^ Copyright 2022, Elsevier. (D) Schematic illustration of the preparation of GCP@PPy hydrogel and the healing mechanism of dynamic Au‐SR bonds in GCP@PPy hydrogel stimulated by NIR laser and electricity. Reproduced with permission.^[^ ^93^ ^]^ Copyright 2019, Wiley‐VCH.

The formation of coordination bonds leads to the establishment of crosslinking points,^[^ ^84^, ^85^, ^86^ ^]^ thereby enhancing the stability and mechanical toughness of the hydrogels. Ren et al. successfully integrated PPy particles into Fe^3+^ crosslinked polyacrylic acid and chitosan hydrogels, developing hydrogels with exceptional conductivity and desirable mechanical properties (Figure 7A).^[^ ^87^ ^]^ The hydrogel networks contained a substantial number of dynamic bonds, including hydrogen bonding interactions between the ‐NH group of PPy and the ─COOH group of PAA, as well as a strong coordination bond between Fe^3+^ and the ─COOH group of PAA and the ─NH_2_ group of chitosan. When subjected to external forces, the dynamic hydrogen and coordination bonds effectively dissipate energy through fracture and reconstruction processes, conferring excellent mechanical properties upon the hydrogel.

Furthermore, by selecting different ligands and central metal ions, the strength and stability of coordination bonds can be adjusted, thereby influencing the mechanical properties, anti‐swelling ability, and other particular functionalities of hydrogels. Lu et al. developed tough, electrically conductive, and biocompatible hydrogels based on coordination bonds by immersing PAM/CS hydrogels adsorbed with pyrrole monomers in Fe^3+^ solution (Figure 7B).^[^ ^88^ ^]^ In this reaction, Fe^3+^ acted as an initiator to trigger pyrrole polymerization and served as a crosslinking agent that formed coordination bonds with CS. These coordination bonds played a crucial role in enhancing the mechanical properties of the hydrogel. Additionally, the formation of coordination bonds increased the crosslinking density, forming a strong polymer network. As a result, the hydrogel maintained a low swelling rate in a wet environment. Zhou et al. prepared a multifunctional dual network SCPH using PAM and carboxymethyl chitosan grafted with PANI as the main materials (Figure 7C).^[^ ^89^ ^]^ The ionic crosslinked network formed by Ag^+^ and CMCS‐g‐PANI was the first network, imparting strength and toughness to the SCPHs. On the other hand, the covalently crosslinked PAM acted as the second network, providing flexibility to the SCPHs. Furthermore, the dual network SCPHs exhibited many supramolecular interactions, encompassing hydrogen bonding, coordination bonding, and electrostatic interactions. In conjunction with covalent and dynamic coordination crosslinks, these interactions conferred upon the SCPHs exceptional tensile strength, reparability, and self‐adhesive properties. Notably, the introduction of Ag^+^ served not only as an ionic crosslinker to bolster the mechanical characteristics of the SCPHs, but also imparted it with antimicrobial properties.

Certain coordination bonds exhibit dynamic reversibility, wherein they undergo dynamic dissociation and reorganization in response to external stimuli, thereby enabling self‐healing.^[^ ^90^, ^91^, ^92^ ^]^ Taking advantage of this property, Yu et al. prepared a stretchable and self‐healable hydrogel electrode material by introducing *N*,*N*‐bis(acryloyl)hemiamine containing disulfide bonds as a functional crosslinking agent into gold nanoparticles/carbon nanotubes/poly(acrylamide@PPy) hydrogels (Figure 7D).^[^ ^93^ ^]^ The hydrogels displayed remarkable healing properties under infrared light and current stimulation, owing to the abundance of dynamic Au‐SR coordination bonds within the polymer network. This highly efficient healing mechanism, triggered by dynamic coordination interactions with multi‐reactivity, exhibited remarkable flexibility and held tremendous potential for the fabrication of multilayer supercapacitors and intelligent responsive materials.

Due to the exceptional strength of coordination bonds, SCPHs incorporating such bonds possess remarkable resistance to both tensile and compressive forces. Simultaneously, the reversible nature of these coordination bonds enables hydrogels to undergo structural changes in response to external stimuli, thus showcasing their inherent self‐healing properties. Moreover, coordination bonds with high bond energies within the hydrogel structure serve as sacrificial bonds, akin to “zippers”, effectively dissipating energy during mechanical deformation, leading to enhanced mechanical properties.^[^ ^94^, ^95^ ^]^ In summary, the metal‐ligand coordination bond emerges as a versatile and robust chemical bond, offering many possibilities for exploring and applying SCPHs.

## 3 PROPERTIES OF SCPHS

SCPHs possess many distinctive attributes and functionalities that unlock novel prospects in modern sensing technology. This material exhibits diverse traits, including exceptional electrical conductivity, remarkable toughness and plasticity, outstanding biocompatibility, adhesive properties, and self‐healing capabilities. These unique features render SCPHs an exceedingly dependable and discerning sensing material. Additionally, its anti‐expansion and anti‐fatigue properties confer the advantage of maintaining stable sensing performance over extended durations, even in demanding environmental conditions. This section presents a comprehensive overview and analysis of SCPHs’ properties that are relevant to sensor applications.

### 3.1 Conductivity

Conductivity plays a pivotal role in the functionality of SCPH sensors. The conductivity of SCPHs originates from the conductive components present in the polymer network, such as PANI, PPy, and PEDOT (Table 1). These components possess a conjugated structure that enables efficient electron transport and conductivity through the conjugated electron system. The exceptional conductivity of SCPHs allows for efficient current conduction, thus facilitating the sensors’ rapid and precise response to external signals. SCPHs integrate the conductor properties of CPs with the porous structure of hydrogels. The conjugated structure of CPs ensures the retention and smooth transfer of electrons within their backbone,^[^ ^96^, ^97^ ^]^ while the porous structure enhances electron transport pathways and contact area within the material. Consequently, SCPHs outperform conventional hydrogels regarding conductivity and charge transfer rate.

**TABLE 1:** Properties of CPs.

CP	Conductivity (S cm^−1^)	Polymerization method	Properties	Limitations	Ref.
PPy	10–7.5 × 10^3^	Chemical oxidative Vapor‐phase polymerization	High electrical conductivity Biocompatibility Easy synthesis Environmental stability	Rigid Brittle Insoluble Non‐transparent	[210, 211, 212]
PANI	27–320	Chemical oxidative Electrochemical	Low cost Easy synthesis Diverse structural forms Environmental stability	Non‐biodegradable Poor solubility Low stretchability Limited processability	[213, 214]
PEDOT	0.4–1000	Vapor‐phase polymerization Chemical oxidative	High conductivity Biocompatibility Good transparency Thermal stability	Poor solubility Low stretchability	[215, 216, 217]

**FIGURE 8:**
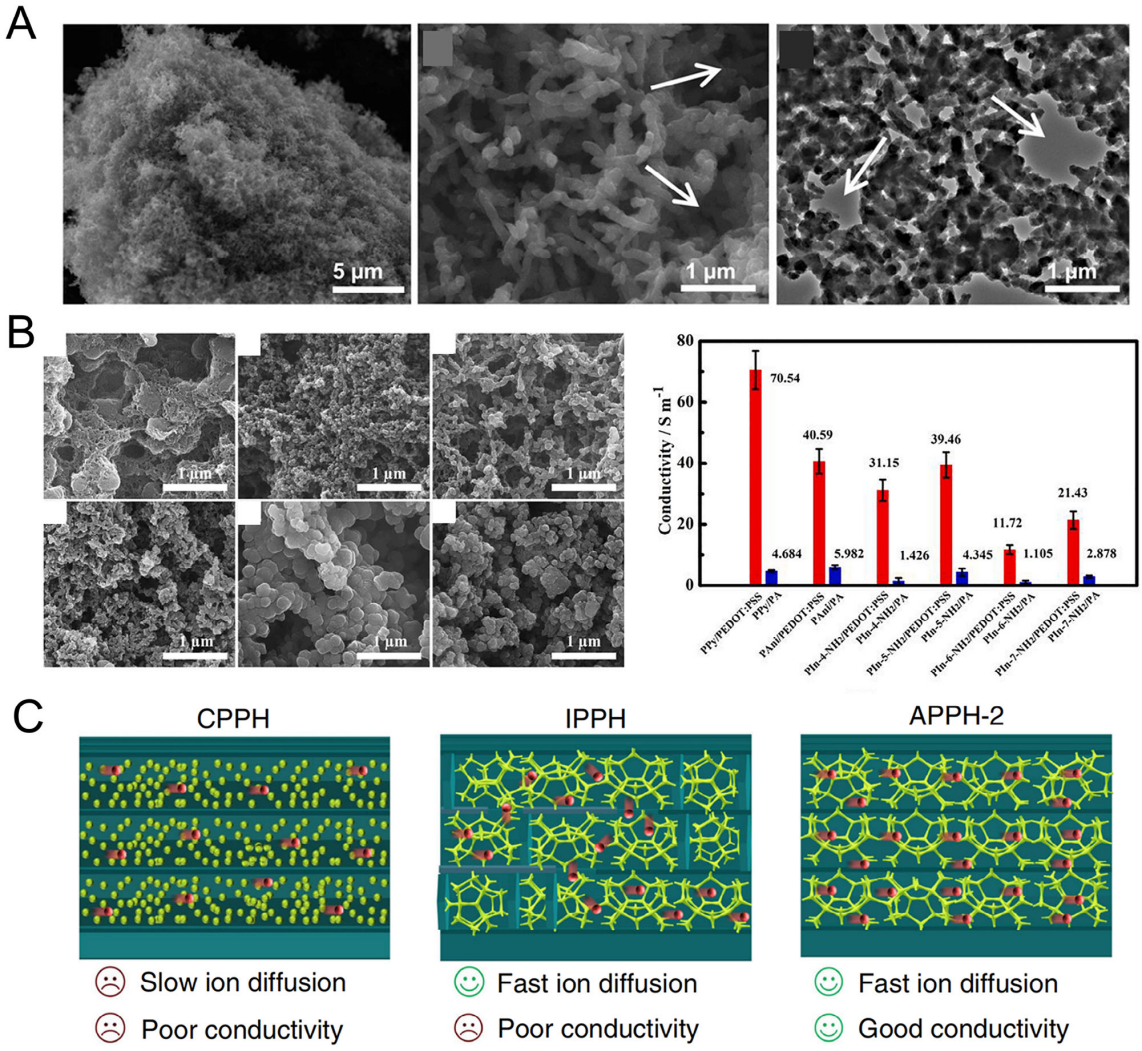
The conductivity of SCPHs. (A) Highly interconnected network structure of PA crosslinked PANI hydrogels. Reproduced with permission.^[^ ^52^ ^]^ Copyright 2012, National Academy of Sciences. (B) Microstructure and conductivity of six PEDOT: PSS crosslinked SCPHs. Reproduced with permission.^[^ ^64^ ^]^ Copyright 2022, Elsevier. (C) Schematic illustration of the effect of microstructure onion and electron transport in SCPHs. Reproduced with permission.^[^ ^98^ ^]^ Copyright 2020, Springer Nature.

When SCPHs are formed through direct crosslinking of pure CPs, they exhibit an ultra‐high conductivity comparable to pure CPs, thanks to the absence of insulating polymer networks. By utilizing supramolecular interactions, it is possible to prepare SCPHs with different conductivities by modulating the structure of the SCPHs network. As discussed in the preceding section, it has been noted that PA can serve as a dopant and crosslinker for crosslinking PANI.^[^ ^52^ ^]^ Through electrostatic interactions between PA and PANI, PANI crosslinks to form a nanoporous structure with a three‐dimensional interconnected network (Figure 8A). This unique structure possesses a larger effective surface area, facilitating the efficient transport of electrons and ions. However, the conductivity of PA itself is limited, consequently restricting the overall conductivity of the SCPHs. To overcome this limitation and achieve higher electrical conductivity in SCPHs, Luo's team proposed using the commercially available conductive polymer PEDOT: PSS as a crosslinking agent instead of PA.^[^ ^64^ ^]^ By leveraging the electrostatic interactions between the negatively charged PEDOT: PSS and the positively charged PANI, PPy, and Poly‐aminoindole, these CPs could be rapidly gelated. As a result, a series of SCPHs with highly interconnected nanoscale conductive networks were formed, providing a significantly larger surface area and porosity, which in turn facilitates charge transfer (Figure 8B). Compared to the PA‐crosslinked SCPHs, the conductivity of SCPHs crosslinked by CPs increased 10–15 times.

Although SCPHs prepared by direct crosslinking of CPs have demonstrated satisfactory electrical conductivity, SCPHs exclusively derived from rigid polymers exhibit limited mechanical strength and lack flexibility, making them unsuitable for applications in complex strain environments. Recently, numerous studies have been on combining CPs and hydrogel matrices to obtain hydrogels with excellent mechanical and conductive properties. In this particular class of SCPHs, the content and dispersion of CPs play crucial roles in governing electrical conductivity. A higher concentration of conductive polymers enhances the establishment of conductive pathways and facilitates efficient charge transfer, thereby increasing the overall conductivity. Additionally, the uniform dispersion of CPs within the SCPHs significantly enhances the interconnectivity of the conductive pathways, enabling unimpeded current conduction throughout the entire material.

For example, Liu et al. adopted a freeze polymerization strategy to fabricate SCPHs comprising PVA and PANI.^[^ ^98^ ^]^ Taking advantage of the directional growth of ice crystals, PANI and PVA chains, which were tightly linked through hydrogen bonding, were assembled into a 3D ordered porous structure using ice as a template. This homogeneous and regular structure greatly facilitated the transport of charges and ions. Contrarily, the non‐uniform distribution of PANI resulted in agglomeration or aggregation phenomena, which led to the interruption or blockage of conductive channels. Moreover, SCPHs with randomly distributed pore structures proved unfavorable for fast charge transfer as the disordered microchannels prolonged the electron transfer pathway. (Figure 8C).

Supramolecular interactions play an essential role in modulating the conductivity of SCPHs. On the one hand, supramolecular interactions enable the crosslinking of CPs, forming a three‐dimensional interconnected mesh structure that facilitates charge transfer. On the other hand, introducing supramolecular interactions reinforces the interaction between the CPs and the hydrogel matrix, effectively improving CPs dispersion and enhancing the electrical conductivity of the SCPHs. Notably, achieving a balance between the mechanical and conductive properties of SCPHs remains a significant concern. To facilitate the comparison of performance disparities among SCPHs fabricated via distinct supramolecular approaches, Table 2 presents a comprehensive overview of the electrical and mechanical characteristics of selected SCPHs. This compilation offers valuable insights into the rational design of SCPHs with diverse performance attributes.

**TABLE 2:** Comparison of electrical conductivity and mechanical properties of SCPHs developed by different supramolecular strategies.

Supramolecular interaction	SCPHs	Conductivity (S m^−1^)	Tensile strength (MPa)	Stretchability	Ref.
HB	PAM/PEDOT: PSS/CNF/LiCl	≈1.75	0.19	400%	[163]
HB	PANI/P(AAm‐co‐HEMA)	8.14	7.27	220%	[59]
HB	PANI NPs@(CS‐PAA))	9.30	0.0484	1144%	[61]
HB	TOCNF/PANI‐PVAB	0.6	0.0156	1530%	[175]
CB	PPy−PAM/CS	0.3	0.8	260%	[88]
CB	PPy/Au/CNT/PAM	≈3	1.25	2380%	[93]
HB/CB	PHEMA/PVA/CNFs@PPy	0.26	≈0.140	2625%	[62]
HB/CB	CS/PAA/PPy/Fe^3+^	2.61	0.33	628%	[87]
HB/CB	PPy‐*g*‐CS/PAA/Fe^3+^	4000	≈0.006	1500%	[120]
HGI	P(NIPAM‐*co*‐β‐CD)/CNT/PPy	34.93	≈0.18	≈515%	[77]
HB/HGI	CB [7]‐PAM/CS/PPy	0.534	0.215	2149.17%	[76]
HB/CB/HGI	PANI/P(AAm‐*co*‐AA) @Fe^3+^	2.78	0.21	442%	[74]
EI	PANI/PA	11			[52]
EI	PPy/PEDOT: PSS	70.54			[64]
EI	PANI/PEDOT: PSS	40.59			
EI	PIn‐5‐NH2/PEDOT: PSS	39.46			
EI	PPy/PA	≈11			[57]
EI	PPy/s‐MWCNTs	30			
EI	HPAA/PANI	≈3.35	0.9	2590%	[66]
HB/EI	Polyion complex/PANI/PA	0.7	1.15	395%	[65]
HB/EI	PPy microgels/P(AAm‐*co*‐LMA)/SDS	1.40	0.196	1950%	[67]
HB/EI	P(AM‐*co*‐SMBA)/TEMPO‐OC/PANI	0.095	0.082	1095%	[139]
HB/CB/EI	PAM/CMCS‐PANI/Ag^+^	0.045	0.185	1100%	[89]

Abbreviations: HB, hydrogen bond; CB, coordination bond; HGI, host‐guest interaction; EI, electrostatic interaction; LMA, lauryl methacrylate; OC, oxidized cellulose.

### 3.2 Toughness

Tough hydrogels have a tensile strength ranging from 0.1 to 1.0 MPa and a fracture energy of 10^2^–10^3^ J m^−1^. These hydrogels play a critical role in wearable sensors, ensuring their reliability and stability without experiencing fractures or damage even when subjected to intricate stress conditions. Nevertheless, most conventional hydrogels’ mechanical strength and stretchability are insufficient, primarily due to the absence of efficient energy dissipation mechanisms. Researchers are committed to investigating and developing new energy dissipation mechanisms and enhancement strategies to overcome these limitations.

**FIGURE 9:**
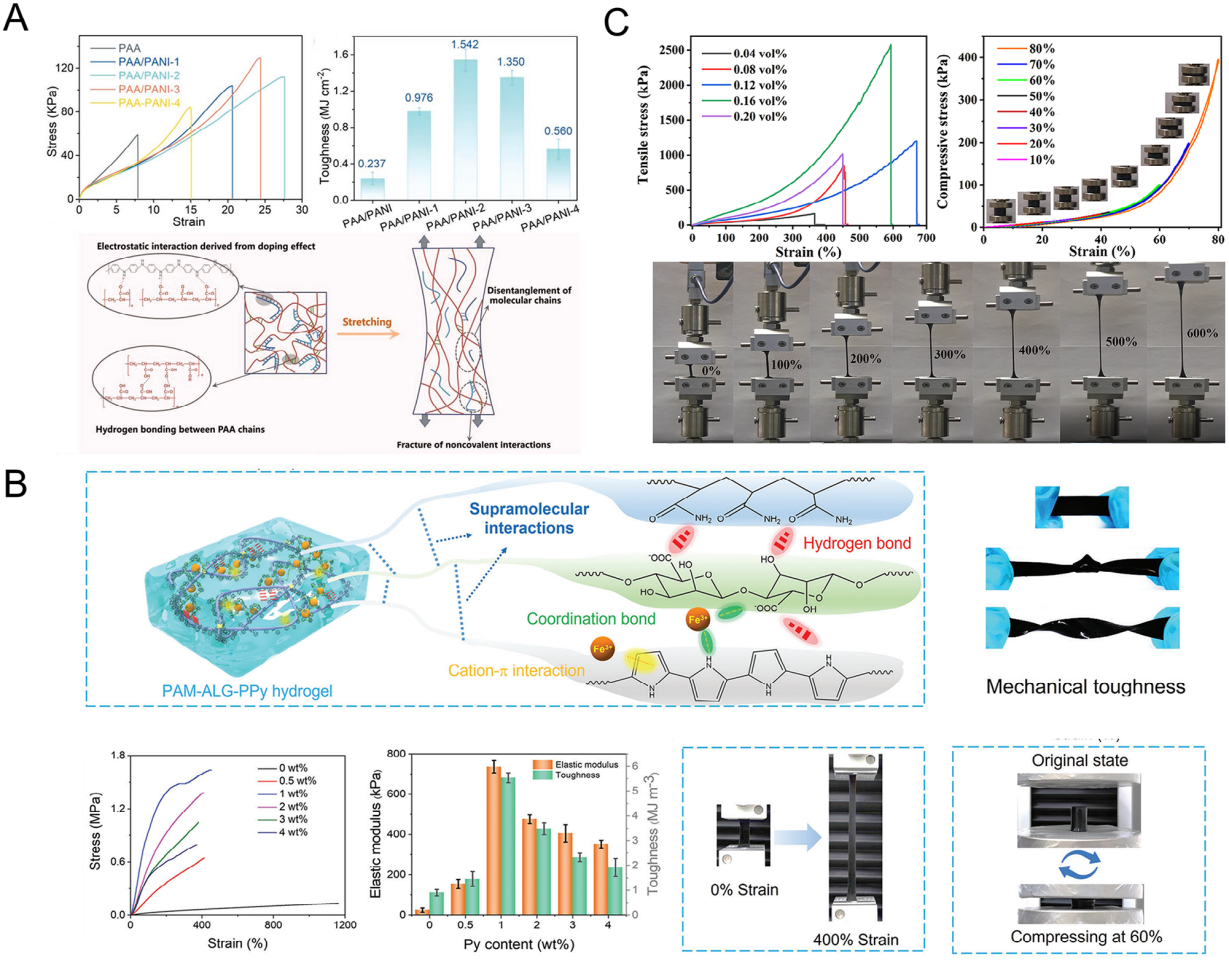
The toughness of SCPHs. (A) Schematic strain mechanism and tensile properties of PAA/PANI hydrogels. Reproduced with permission.^[^ ^99^ ^]^ Copyright 2022, Wiley‐VCH. (B) Schematic diagram of the multiple supramolecular interactions in the hydrogel network and the excellent mechanical properties of PAM‐ALG‐PPy hydrogels. Reproduced with permission.^[^ ^100^ ^]^ Copyright 2023, Wiley‐VCH. (C) Mechanical performance of PVA/PA/PANI/GA hydrogels and their tensile photographs. Reproduced with permission.^[^ ^102^ ^]^ Copyright 2021, American Chemical Society.

Introducing supramolecular interactions into the SCPHs network can significantly enhance the mechanical properties of hydrogels. Under external forces, these supramolecular interactions consume substantial energy through dissociation, thereby enabling SCPHs to exhibit enhanced resistance against external pressures. In a recent study by Chen et al., a supramolecular hydrogel was synthesized using polyacrylic acid (PAA) and polyaniline (PANI) as the main components.^[^ ^99^ ^]^ The formation of high‐density electrostatic interactions occurred between the negatively charged carboxyl groups on PAA and the positively charged imine groups on PANI. This dynamic electrostatic interaction underwent slippage or rupture when subjected to external forces, effectively impeding crack propagation and resulting in the hydrogels’ remarkable tensile strength (2830%) and toughness (1.54 MJ m^−1^) (Figure 9A).

Multiple supramolecular interactions have a synergistic effect. Initially, these interactions reinforce the bonding between polymer networks, enabling uniform transfer of loads to the SCPHs network. These energy dissipation mechanisms effectively allow the hydrogel to withstand higher stresses. Huang's team developed a multinetwork SCPH by initiating PPy polymerization in situ within covalently crosslinked PAM/sodium alginate interpenetrating network hydrogels, utilizing Fe^3+^ as an initiator.^[^ ^100^ ^]^ Fe^3+^ not only engaged in cation‐π interactions and coordination with the in situ generated PPy, but also formed coordination bonds with the carboxyl groups of sodium alginate. Furthermore, the copious hydroxyl and carboxyl groups in sodium alginate facilitated the formation of abundant hydrogen bonds between PAM and PPy, thereby promoting the establishment of strong bonds between the hydrophobic PPy and the hydrophilic polymer. When subjected to external forces, the numerous hydrogen bonds, coordination bonds, and cation‐π interactions with substantial binding energies between the polymers functioned as energy dissipation mechanisms, effectively dissipating energy. Due to the efficient interactions between the polymer networks, the resulting supramolecular hydrogels exhibited uniform structural integrity and stability, displaying remarkable tensile strength (1.63 MPa), elongation at break (453%), and exceptional toughness (5.5 MJ m^−1^) (Figure 9B). Notably, the external force diffuses uniformly throughout the polymer network owing to the effective connections established via supramolecular interactions, ensuring uniform deformation of the hydrogel during stretching and exhibiting a linear change in the electrical signal. Similarly, Wang and colleagues designed an interpenetrating synergistic bi‐network SCPH comprising polyurethane and PANI.^[^ ^101^ ^]^ These SCPHs incorporate various dynamic interactions, including hydrogen bonding, ionic interactions, and diols‐alder covalent bonding. These dynamic bonds contribute to the hydrogels’ excellent tensile strength (1.1 MPa), ductility (500%), and self‐healing properties following fracture.

Yang et al. prepared a biocompatible, flexible, and robust SCPH by introducing phytate‐doped PANI into glutaraldehyde‐crosslinked polyvinyl alcohol (PVA).^[^ ^102^ ^]^ The introduction of phytic acid facilitated the formation of ester bonds with PVA, promoting crosslinking of the polymer chains. Additionally, electrostatic interactions occurred between PANI and PVA, further enhancing the stability of the hydrogel structure. Moreover, abundant hydrogen bonding between PANI and PVA polymers contributed to the unique multiple synergistic networks. These SCPHs comprised strong chemical covalent bonds and numerous weak physical crosslinks, resulting in excellent mechanical properties of the hydrogel (tensile strength = 1.2 MPa, elongation at break = 670%) (Figure 9C). Interestingly, PANI also exhibited a plasticizing effect, rendering the hydrogel more flexible. Furthermore, the weak physical interactions between PANI and PVA enhanced mechanical strength and elasticity. Notably, the rapid dissociation and crosslinking of the relatively weak physical crosslinks between the polymer chains effectively dissipated energy, enabling the hydrogel network to recover during the loading‐unloading process.

By introducing supramolecular interactions in SCPHs, the interaction force and crosslinking density between polymer molecular chains can be effectively increased, thereby substantially improving the materials’ mechanical strength and tensile properties. These interactions serve as an effective energy dissipation mechanism, efficiently absorbing and dispersing external stresses to safeguard the polymer chains against fracture. By conducting comprehensive investigations into supramolecular interactions, the performance of SCPHs can be further enhanced, enabling the fulfillment of a broader range of functional demands.

### 3.3 Biocompatibility

Biocompatibility is one of the most critical characteristics of wearable sensors.^[^ ^103^, ^104^ ^]^ Given their prolonged contact with the human body, any shortcomings in material biocompatibility can potentially trigger allergic reactions, tissue damage, and other undesirable consequences, compromising user comfort and impeding sensor functionality. Therefore, developing sensor materials with good biocompatibility is essential to ensure the long‐term stability and safety of the sensors. In recent years, more investigators have tried improving the compatibility between sensors and organisms by optimizing the composition and structural design of SCPHs.

**FIGURE 10:**
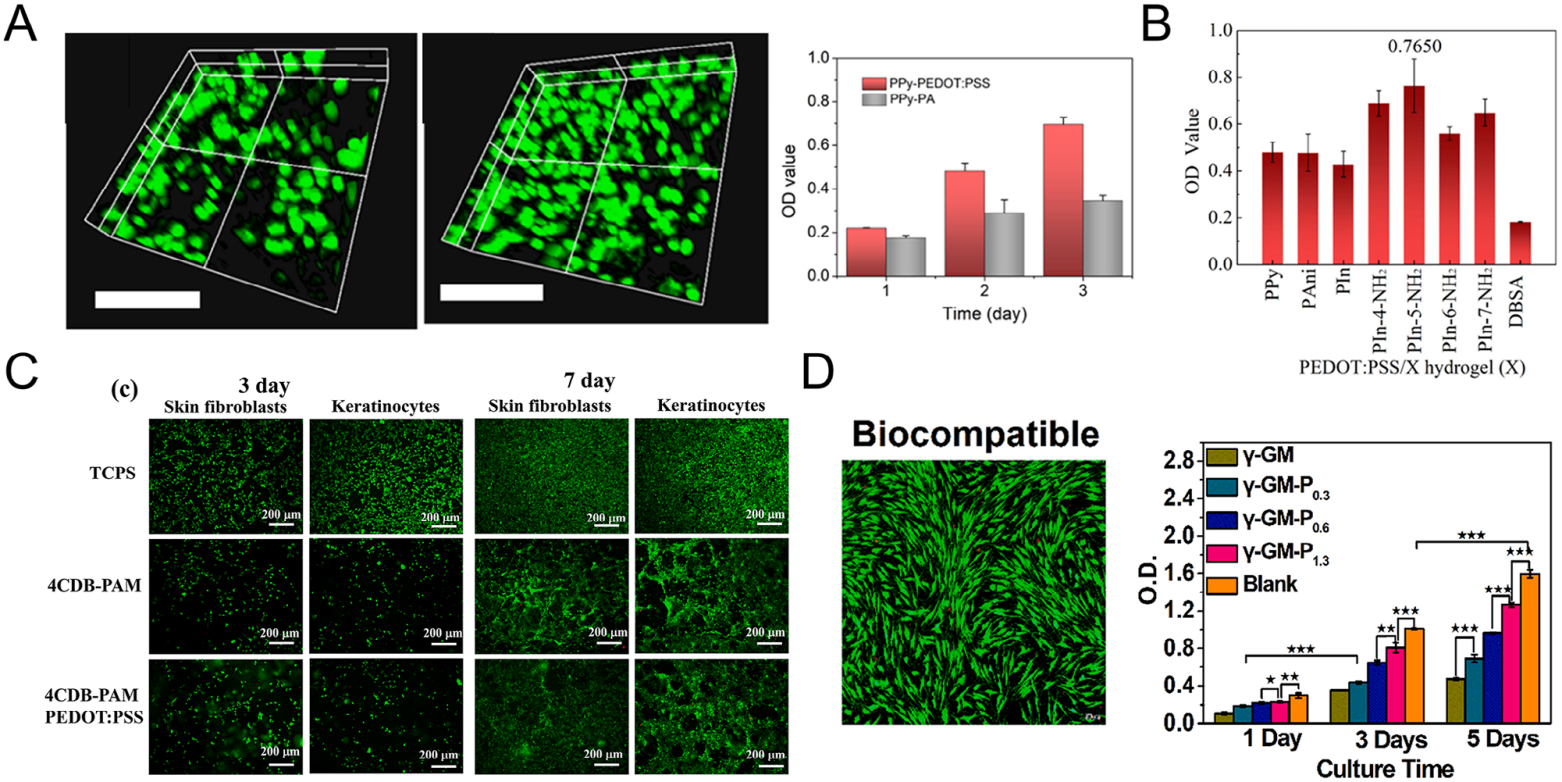
The biocompatibility of SCPHs. (A) Biocompatibility of PEDOT: PSS hydrogels crosslinked with PPy. Reproduced with permission.^[^ ^105^ ^]^ Copyright 2021, American Chemical Society. (B) Viability of PC12 cells cultured on six PEDOT: PSS crosslinked hydrogels after 48 h. Reproduced with permission.^[^ ^106^ ^]^ Copyright 2023, Royal Society of Chemistry. (C) The SCPHs based on carboxymethyl CMC‐DA/PEDOT: PSS exhibit excellent biocompatibility, facilitating the proliferation of skin fibroblasts and keratinogenic cells. Reproduced with permission.^[^ ^107^ ^]^ Copyright 2021, Elsevier. (D) The γ‐GM‐P hydrogel is highly biocompatible and friendly to human skin fibroblasts. Reproduced with permission.^[^ ^108^ ^]^ Copyright 2022, Elsevier.

The preparation process of SCPHs is often overly complex and involves the addition of toxic substances, resulting in poor biocompatibility. A recent study by Luo et al. investigated the incorporation of PPy into a commercially available conductive polymer, PEDOT: PSS, to develop a SCPH with electrical conductivity and biocompatibility.^[^ ^105^ ^]^ By leveraging the electrostatic interactions between negatively charged PSS and positively charged PPy, PEDOT: PSS served as a conductive component and a biocompatible crosslinking agent, facilitating rapid gel formation with PPy without additional crosslinking agents. Cytotoxicity tests demonstrated the high biocompatibility of the PPy‐PEDOT: PSS hydrogel, with its 3D porous structure promoting cell growth and 3D culture (Figure 10A). Similarly, Luo et al. developed a method to crosslink positively charged PPy using negatively charged groups on sulfonated carbon nanotubes, resulting in a highly biocompatible SCPH for cellular sensing.^[^ ^57^ ^]^ In their most recent work, Luo proposed a novel approach for preparing SCPHs by crosslinking PEDOT: PSS with a positively charged polymer. They investigated the gelation of various positively charged conducting polymers, such as polyindoles and their derivatives, and PPy and PANI, through electrostatic interactions with PEDOT: PSS molecular chains.^[^ ^106^ ^]^ The resulting SCPHs exhibited excellent electrical conductivity and biocompatibility, as they did not require the introduction of insulating polymers or toxic substances (Figure 10B). These findings highlight the high biocompatibility of hydrogels composed solely of conducting polymers, demonstrating their immense potential for bioelectronic device applications.

Natural polymers, renowned for their abundant sources and exceptional biocompatibility, have widely been used to fabricate bio‐friendly materials. In a recent study by Han et al. a composite SCPH was developed for bioelectronic applications, employing a combination of dopamine‐modified carboxymethylcellulose (CMC‐DA), PAM, and PEDOT: PSS.^[^ ^107^ ^]^ Biocompatibility experiments demonstrated that the SCPHs exhibited remarkable compatibility with skin cells, with the incorporation of CMC‐DA proving particularly advantageous for enhancing the value‐added properties of skin cells (Figure 10C). In a separate study by Liu et al., a SCPH was fabricated using γ‐polyglutamic acid (PGA) and PEDOT: PSS.^[^ ^108^ ^]^ The exceptional biocompatibility of *γ*‐polyglutamic acid rendered the composite hydrogel highly cytocompatibility, thereby promoting the proliferation of human skin fibroblasts and ensuring the safety of the hydrogel sensor upon contact with human skin (Figure 10D).

The biocompatibility of the sensor can be improved by selecting functional molecules and polymers with favorable biocompatibility as the primary materials for constructing the SCPHs. Firstly, good biocompatibility can minimize the adverse reactions between the sensor and the organism, thus reducing the damage and irritation to the organism and improving the safety of the sensor. Additionally, exceptional biocompatibility facilitates optimal contact between the sensor and the organism's surface, enabling the sensor to perceive and record changes occurring within the organism more effectively. The biocompatibility investigation provides novel insights for the advancement of SCPH sensors and holds immense potential for future breakthroughs in biomedical engineering.

### 3.4 Self‐adhesive

Adhesion performance refers to the ability of the material surface to adhere to other substances, constituting a pivotal determinant in the detection efficacy of hydrogel sensors.^[^ ^109^, ^110^ ^]^ Primarily, superior adhesion guarantees the efficient affixation of the sensor onto the surface of the intended object, thereby facilitating the conversion of mechanical deformation into electrical signals while minimizing signal attenuation. Furthermore, resilient adhesion between the sensor and the target object thwarts detachment or displacement arising from motion or shear forces, consequently heightening the stability and dependability of the sensor.

**FIGURE 11:**
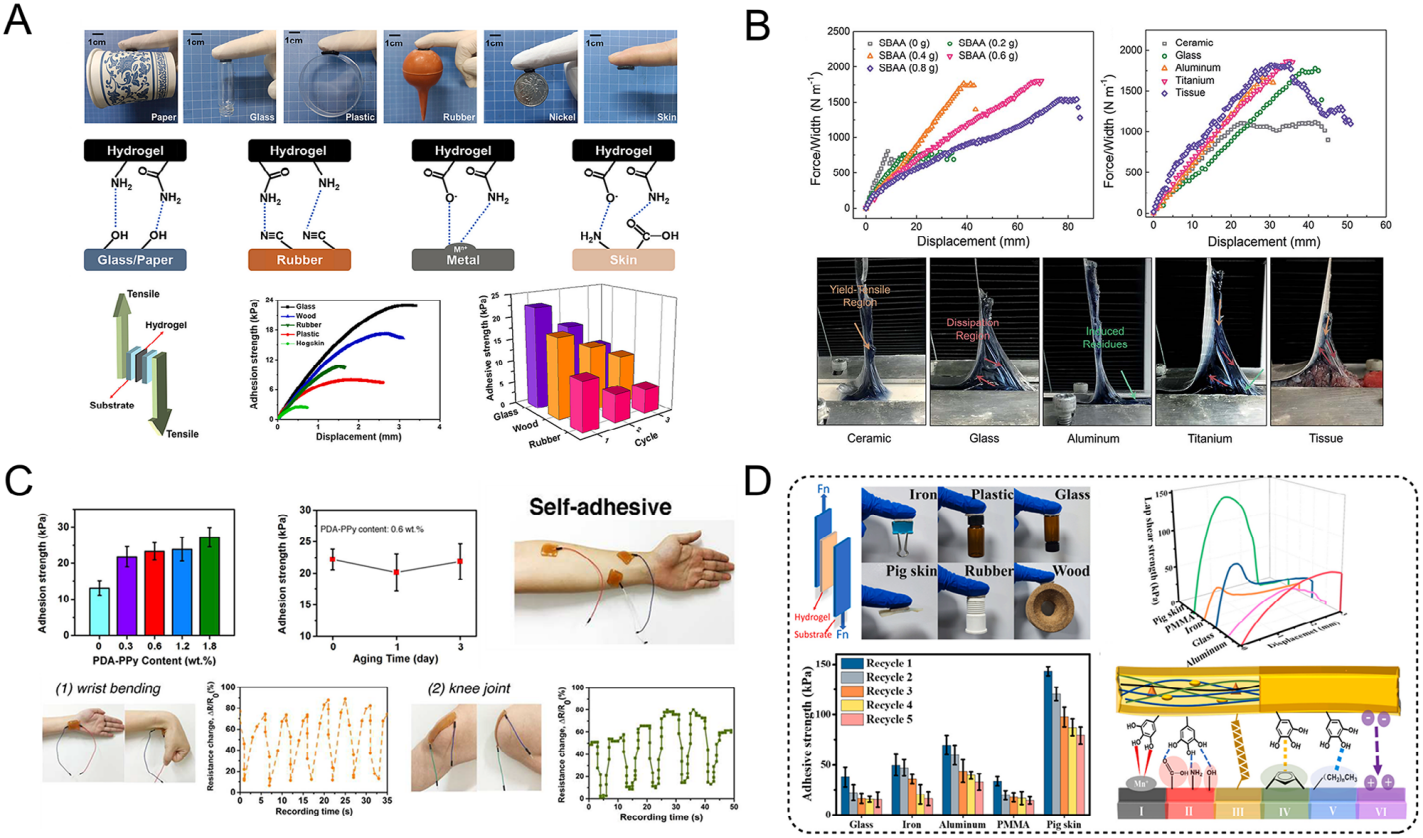
The self‐adhesive properties of SCPHs. (A) Adhesive performance and adhesive mechanism of PAM/CMCS‐*g*‐PANI/Ag DN hydrogels. Reproduced with permission.^[^ ^89^ ^]^ Copyright 2022, Elsevier. (B) Adhesion performance and adhesion instances of poly (HEAA‐co‐SBAA)/PEDOT: PSS hydrogels with various substrates. Reproduced with permission.^[^ ^111^ ^]^ Copyright 2020, Royal Society of Chemistry. (C) Self‐adhesion of PDA‐PPy‐PAM and sensing applications of the hydrogels adhering to the human body. Reproduced with permission.^[^ ^115^ ^]^ Copyright 2018, American Chemical Society. (D) The adhesion strength and adhesion mechanism of PHEMA/PVA/CNFs@PPy to different substrates. Reproduced with permission.^[^ ^62^ ^]^ Copyright 2023, Elsevier.

It has been shown that when constructing SCPHs networks, designing some groups that easily form strong interactions with different substrates can effectively enhance the adhesion between the hydrogel and the target surface. For example, Zhou et al. combined covalently crosslinked PAM with physically crosslinked carboxymethyl chitosan grafted polyaniline (CMCS‐g‐PANI) network to prepare ionic/electronic conducting dual network hydrogels.^[^ ^89^ ^]^ The double network hydrogels, containing abundant hydroxyl, carboxyl, and amide groups, exhibited reversible physical interactions with diverse substrates, leading to robust, long‐lasting, and reproducible adhesion to a wide range of materials such as paper, glass, plastics, rubber, and metals (Figure 11A). In another study, Zheng et al. synthesized a SCPH by interpenetrating a PEDOT: PSS conducting polymer into an amphiphilic ionic poly(HEAA‐*co*‐SBAA) network, which relied on hydrogen and electrostatic interactions.^[^ ^111^ ^]^ The hydrogel exhibited strong surface adhesion on different nonporous solid surfaces, including glass, titanium, ceramics, aluminum, and beef tissue (Figure 11B). The researchers have deduced that the pivotal determinant impacting surface adhesion lies in the interplay between the hydrogel and diverse substrates, contingent upon the surface's chemical and physical lattice structure.

The mussel, a mollusk inhabiting marine environments, exhibits remarkable adhesive properties that allow it to firmly attach to various surfaces in wet conditions. This adhesive ability stems primarily from the secretion of mussel adhesion proteins rich in catechol functional groups (catechol moieties). These groups play a pivotal role in adhesion by engaging in diverse physical and chemical interactions with substrate surfaces.^[^ ^112^, ^113^, ^114^ ^]^ The adhesive mechanisms inspired by mussels have extensive applications in fabricating diverse adhesive hydrogels. For example, Lu et al. prepared a transparent, stretchable and self‐adhesive SCPH by introducing polydopamine‐doped polypyrrole (PDA—PPy) nanofibers into an elastic PAM matrix.^[^ ^115^ ^]^ PDA enhanced the hydrophilicity of PPy through π‐π stacking and hydrogen bonding, facilitating the uniform dispersion of PPy within the hydrogel network to establish a complete conductive pathway. Including catechol moieties from PDA‐PPy nanofibers imparted strong self‐adhesion to the SCPHs, mimicking the mussel‐inspired adhesion mechanism (Figure 11C). Similarly, tannic acid, a natural polyphenolic compound abundant in catechol moieties, has been widely employed in the development of self‐adhesive hydrogels.^[^ ^116^ ^]^ Wang's team proposed a gelation strategy based on a polyphenol (TA)‐metal ion (Fe^3+^) catalytic system to prepare PPy‐doped modified cellulose nanofibers (CNFs@PPy) in PVA/PHEMA hydrogels.^[^ ^62^ ^]^ Leveraging the abundance of catechol groups on the TA surface, the resulting hydrogel formed numerous bonds with different solid substrates, enabling easy adhesion to diverse substrates and irregular epidermis (Figure 11D).

Adhesive properties can be achieved by adding binder molecules to enhance the interaction of SCPHs with different substrate surfaces. This characteristic endows SCPHs with immense potential for utilization in wearable sensors, biomedical devices, and tissue engineering applications.

### 3.5 Self‐healing

Self‐healing performance refers to the ability of a material or device to autonomously restore its original function or structure after being damaged.^[^ ^117^, ^118^, ^119^ ^]^ In practical applications, wearable sensors necessitate attachment to the human body's surface, rendering them susceptible to friction and mechanical stress, thereby leading to damage or rupture. Introducing self‐healing properties empowers wearable sensors to autonomously mend and reinstate their innate functionality and performance after damage. This capability prolongs the sensor's lifespan, curtails maintenance and replacement expenses, and enhances its stability and dependability in intricate environments. Consequently, investigating the self‐healing performance holds immense importance in advancing the development and utilization of wearable sensors. There have been numerous reports on obtaining sensor materials with self‐healing properties by introducing dynamic supramolecular interactions in SCPHs to achieve long‐term stable operation of sensors.

**FIGURE 12:**
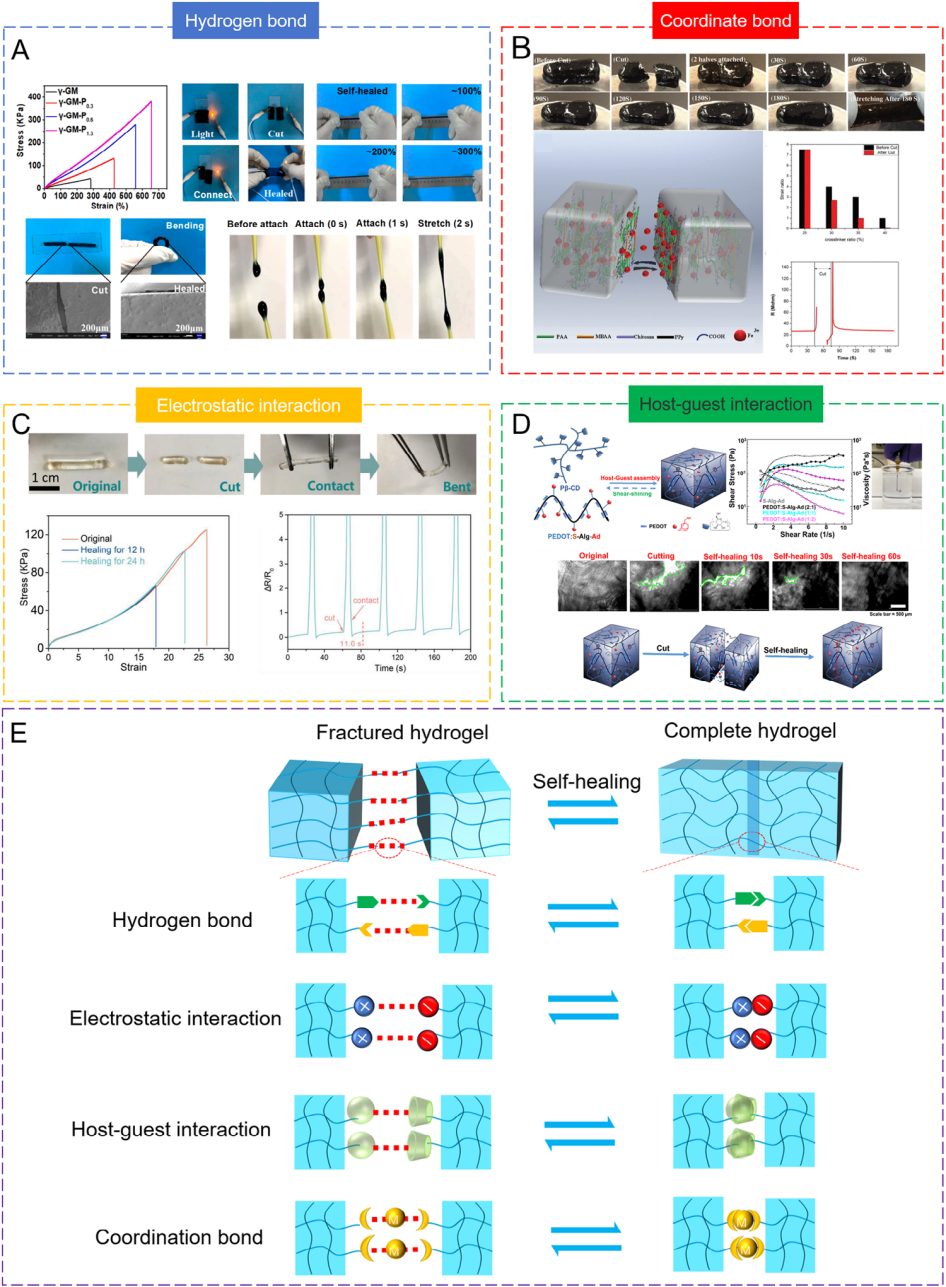
The self‐healing properties of SCPHs. (A) Self‐healing properties of γ‐GM‐P hydrogels formed by multiple hydrogen bonding and demonstration of their self‐healing behavior. Reproduced with permission.^[^ ^108^ ^]^ Copyright 2022, Elsevier. (B) Schematic mechanism of self‐healing and self‐healing performance of SCPHs based on coordination bonds. Reproduced with permission.^[^ ^120^ ^]^ Copyright 2017, Wiley‐VCH. (C) Photographs of self‐healing behavior of PAA/PANI hydrogels based on electrostatic interactions and tensile properties and conductivity of the hydrogels after self‐healing. Reproduced with permission.^[^ ^99^ ^]^ Copyright 2022, Wiley‐VCH. (D) Schematic illustration of the formation of dynamically crosslinked PEDOT: S‐Alg‐Ad/Pβ‐CD hydrogels through host‐guest interactions and the scheme of the hydrogel self‐healing process. Reproduced with permission.^[^ ^121^ ^]^ Copyright 2019, American Chemical Society. (E) The mechanism diagram of self‐healing by different supramolecular interactions.

Multiple hydrogen bonds are highly dynamic, reversible and strongly attractive. When a material is fractured or damaged, multiple hydrogen bonds can quickly attract and guide molecules to rearrange themselves, restoring intermolecular connections. Gao's team introduced multiple hydrogen bonds into γ‐polyglutamic acid and PEDOT: PSS polymer networks to develop a SCPH with good adhesion and self‐healing capabilities.^[^ ^108^ ^]^ The SCPHs exhibited excellent self‐healing properties due to the strong interaction of multiple hydrogen bonds. Notably, when two separated pieces of the SCPHs were reconnected, they swiftly regained their mechanical properties without requiring any external stimuli. (Figure 12A). Moreover, the damaged hydrogel recovered its electrical conductivity and tensile properties following repair.

Because breaking and reorganizing coordination bonds is a dynamic process, the SCPHs based on coordination bonds also possess self‐healing properties. Xing and colleagues successfully synthesized self‐healing SCPHs by chemically polymerizing precursors of acrylic acid, iron ions, and PPy‐grafted chitosan, thereby establishing physical and chemical crosslinking networks.^[^ ^120^ ^]^ The chemically crosslinked PAA chains played a crucial role in enhancing the mechanical properties of the hydrogels, while the dynamic coordination between the PAA and PANI chains endowed the hydrogels with rapid self‐healing capabilities. When two cut pieces of hydrogels came into contact, the iron ions migrated from one side to the other, driven by the reversible coordination interactions between the carboxyl group of PAA, the NH group of PPy, and the iron ions, leading to healing (Figure 12B). Furthermore, the hydrogen bonding between CS and PAA contributed to stabilizing the formed hydrogels and improving their self‐healing properties. This combination of chemical and physical crosslinking established a delicate balance among the PPy, CS, and PAA molecular chains, resulting in a dual‐network hydrogel with remarkable stretchability, and excellent mechanical and electrical self‐healing properties.

When a SCPH comprised of polymer chains with opposite charges undergoes fracture, it relies on electrostatic interactions to facilitate the movement and recombination of the polymer chains at the site of damage. Li et al. devised a SCPH by employing lipoic acid, sodium methacrylate sulfonic acid, and PANI as constituent materials, which capitalizes on the combined effects of hydrogen and electrostatic interactions.^[^ ^99^ ^]^ This synergistic interplay between the rigid CP and the flexible polymer engendered robust interactions, endowing the hydrogel with exceptional toughness, resistance to notches, and puncture resistance. Moreover, the SCPHs exhibited a glass transition temperature lower than room temperature, affording it favorable flow properties at ambient conditions. This attribute enabled rapid self‐healing via dynamic hydrogen and electrostatic interactions (Figure 12C).

Host‐guest interactions represent a class of highly efficient non‐covalent interactions that exhibit characteristics akin to covalent interactions. These interactions possess a remarkable binding strength and demonstrate dynamic reversibility. By capitalizing on the host‐guest design, a supramolecular hydrogel with efficient self‐healing properties was developed by Zhang et al.^[^ ^121^ ^]^ By capitalizing on the host‐guest design, a supramolecular hydrogel with efficient self‐healing properties was developed by Zhang et al. This study blended a composite material comprising PEDOT: adamantane‐modified sulfonated alginate with poly(β‐cyclodextrin), utilizing the host‐guest interaction between adamantane and cyclodextrin. Owing to the dynamic nature of the host‐guest interaction, the resulting hydrogel exhibited non‐Newtonian shear thinning behavior, injectability and self‐healing properties. The hydrogel demonstrated rapid self‐healing upon contact without external stimuli (Figure 12D). The self‐healing properties of these SCPHs could be attributed to the highly dynamic host‐guest interactions, which facilitated the rearrangement and stacking of the PEDOT polymer network. The *π–π* stacking between the PEDOT polymers and the anion‐π interactions with sulfonated alginate further stabilized the repaired composite hydrogel.

Self‐healing properties can be obtained by introducing supramolecular interactions, including hydrogen bonds, coordination bonds, host‐guest interactions, and electrostatic interactions, into the SCPHs network. This has been attributed to the reversible nature of these supramolecular interactions, which allows the damaged hydrogel network to undergo reassembly and reconnection, restoring its original structure and properties upon the dissipation or attenuation of external stimuli (Figure 12E). For SCPHs wearable sensors, integrating self‐healing properties enhances the device's reliability and stability, prolongs its lifespan and mitigates maintenance costs.

### 3.6 Anti‐swelling

Anti‐swelling is the ability of a material to maintain its structural and performance stability when subjected to conditions such as water absorption or wetting. In sensor applications, SCPHs serve as an essential component of the sensing element, whose performance stability is critical to the accuracy and reliability of the sensor. However, SCPHs that contain many hydrophilic polymers are prone to swelling upon water absorption. This swelling phenomenon leads to the deterioration of mechanical and conductive properties, consequently impacting the sensor's accuracy and stability.^[^ ^122^, ^123^, ^124^ ^]^ Improving the anti‐swelling property of SCPHs can effectively solve this problem. SCPHs with good anti‐swelling properties can absorb water while maintaining their structural stability, preventing excessive expansion, thus reducing the structural changes in sensor interference.

**FIGURE 13:**
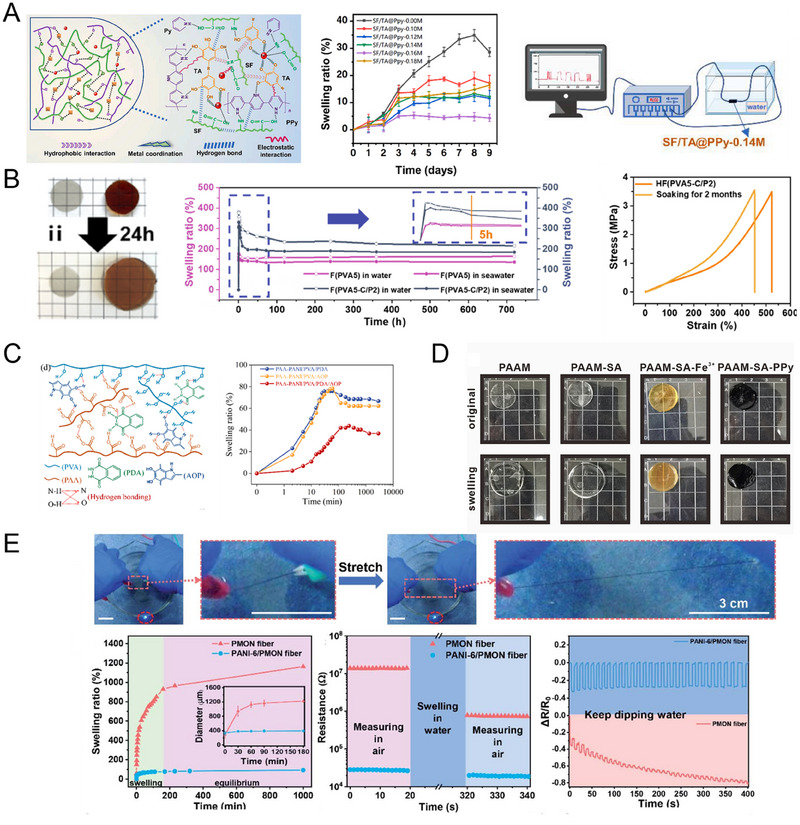
The anti‐swelling properties of SCPHs. (A) Schematic diagram of multiple interactions within SF/TA@PPy hydrogel networks and their swelling curves and underwater sensing applications. Reproduced with permission.^[^ ^125^ ^]^ Copyright 2022, Elsevier. (B) Demonstration of swelling resistance of HF(PVA‐C/P) hydrogels and their swelling curves and mechanical properties after soaking in water. Reproduced with permission.^[^ ^126^ ^]^ Copyright 2021, American Chemical Society. (C) Schematic illustration of multiple hydrogen bonds within the PAA‐PANI/PVA/PDA/AOP hydrogel network and their anti‐swelling properties. Reproduced with permission.^[^ ^127^ ^]^ Copyright 2023, Elsevier. (D) Demonstration of anti‐swelling behaviors of PAAm‐SA‐PPy hydrogels. Reproduced with permission.^[^ ^128^ ^]^ Copyright 2023, Elsevier. (E) Photographs of PANI‐6/PMON hydrogel working underwater and characterization of its resistance to swelling. Reproduced with permission.^[^ ^129^ ^]^ Copyright 2021, Royal Society of Chemistry.

The strong interaction between water molecules and the hydrophilic polymer chains in SCPHs weakens the interactions between the polymer chains. As a result, the polymer chains of SCPHs become more loosely arranged and undergo rapid swelling upon water absorption. To address this issue, researchers have explored methods to enhance the interactions between the SCPHs polymer chains, thereby inhibiting the penetration of water molecules and significantly improving the swelling resistance. For example, Zou et al. developed multifunctional SCPHs by incorporating multiple supramolecular interactions through in situ polymerization of PPy in the system comprising natural polymeric filipin (SF) and natural polyphenolic compound tannic acid (TA).^[^ ^125^ ^]^ Introducing PPy into the hydrogel decreased the swelling rate as the PPy content increased. This swelling resistance improvement was attributed to various forms of supramolecular interactions between PPy and SF/TA, including hydrophobic interactions, hydrogen bonding, and electrostatic interactions (Figure 13A). These interactions effectively bound the polymer chains, stabilizing the SCPHs structure even when submerged in water. This anti‐swelling hydrogel found applications in the air and demonstrated potential for underwater motion monitoring and information transmission.

The incorporation of hydrogen bonding serves to enhance the physical crosslinking points of SCPHs, consequently leading to heightened interaction forces between the polymer chains. As a result, the resistance against water molecule penetration is effectively increased. For instance, Gao et al. devised SCPHs membranes with well‐ordered crystal structures using sodium carboxymethyl cellulose‐modified PPy (C/P) and poly(vinyl alcohol) (PVA) as the primary constituents.^[^ ^126^ ^]^ The homogeneously dispersed C/P and PVA polymer networks formed hydrogen bonds, yielding electrically conductive films with dense crystal structures. Furthermore, the infiltration of glycerol and water molecules into the hydrogel network establishes hydrogen bonds with the molecular chains of the polymers. Due to the organized crystal structure and robust hydrogen bonding within the internal architecture of the hydrogel membrane, exceptional resistance to swelling is maintained across a broad range of liquid environments. Even after prolonged submersion underwater, the hydrogel membranes exhibit outstanding mechanical strength, significantly expanding the potential applications of hydrogel sensors (Figure 13B). Similarly, Yan's team prepared multifunctional SCPHs by introducing multiple hydrogen bonding interactions into PAA‐PANI and PVA interpenetrating polymer networks.^[^ ^127^ ^]^ The introduction of multiple hydrogen bonds between the polymer networks intensified the degree of crosslinking within the hydrogel, resulting in reduced water absorption and a low swelling rate (Figure 13C). It was also demonstrated that more hydrogen bonds effectively diminished the swelling of the hydrogel. In contrast, the recombination of hydrogen bonds facilitated the removal of excess water from the hydrogel once it reached the swelling equilibrium.

By incorporating coordination bonds into the structure of SCPHs, the crosslinking density can be enhanced, resulting in a more compact network structure. This modification has been demonstrated to significantly improve the stability of SCPHs in humid environments. Huang's team successfully synthesized dual‐network SCPHs through in situ polymerization of pyrrole in the presence of iron ions, utilizing a covalently crosslinked polyacrylamide/sodium alginate hydrogel matrix as a template.^[^ ^128^ ^]^ The inclusion of Fe^3+^ ions played a pivotal role in creating the ionic crosslinked SCPHs network. Not only did Fe^3+^ act as an oxidant to initiate pyrrole polymerization, but it also formed coordination bonds with the carboxyl groups present on the sodium alginate that interpenetrated the covalently crosslinked PAM network. The formation of coordination bonds effectively suppressed the swelling of hydrogels in water. This was attributed to the coordination between Fe^3+^ and sodium alginate, increasing the crosslinking density and forming a more robust network (Figure 13D).

Moreover, the CP's inherent hydrophobicity serves as a diffusion barrier, effectively impeding the continuous diffusion of water molecules into the hydrogel network. As a result, the swelling behavior of the SCPHs in an aqueous environment is significantly suppressed. In a recent study by Zhu et al., a resilient hybrid hydrogel fiber with a heterogeneous network was developed by incorporating hydrophobic polyaniline (PANI) into a nanocomposite gel fiber.^[^ ^129^ ^]^ The hydrophobic characteristic of PANI conferred the composite hydrogel with exceptional resistance to swelling. Compared to PANI‐free hydrogels, incorporating PANI significantly augmented the hydrogel's stability in aqueous surroundings, effectively counteracting the deterioration of hydrogel performance triggered by structural alterations arising from swelling. This pivotal advance demonstrated the feasibility of anti‐swelling SCPHs to operate in aqueous environments (Figure 13E).

By introducing hydrogen bonding, coordination bonding etc., into the SCPHs network, the interaction and crosslinking density of the polymer network can be enhanced, thereby effectively impeding the continuous infiltration of water molecules into the polymer network. This improved resistance to swelling minimizes the interference caused by water molecules in hydrogel sensors, thereby ensuring the accuracy and stability of sensor output and broadening the sensor's applicability in humid environments.

### 3.7 Fatigue resistance

Fatigue resistance refers to the capacity of a material to sustain its structural integrity and performance stability when exposed to long‐term or repeated cyclic stress loading.^[^ ^130^, ^131^ ^]^ In the context of sensors, fatigue resistance plays a pivotal role. This is because sensors often undergo frequent stress loading and deformation during their operational lifespan, such as tensile, compression, and twisting forces. The sensor material may experience fatigue‐induced damage without robust fatigue resistance, resulting in performance deterioration or even complete failure. As the pivotal constituent of the sensor, endowing SCPHs with exceptional fatigue resistance undoubtedly emerge as a critical facet in enhancing the performance and reliability of the sensor.

**FIGURE 14:**
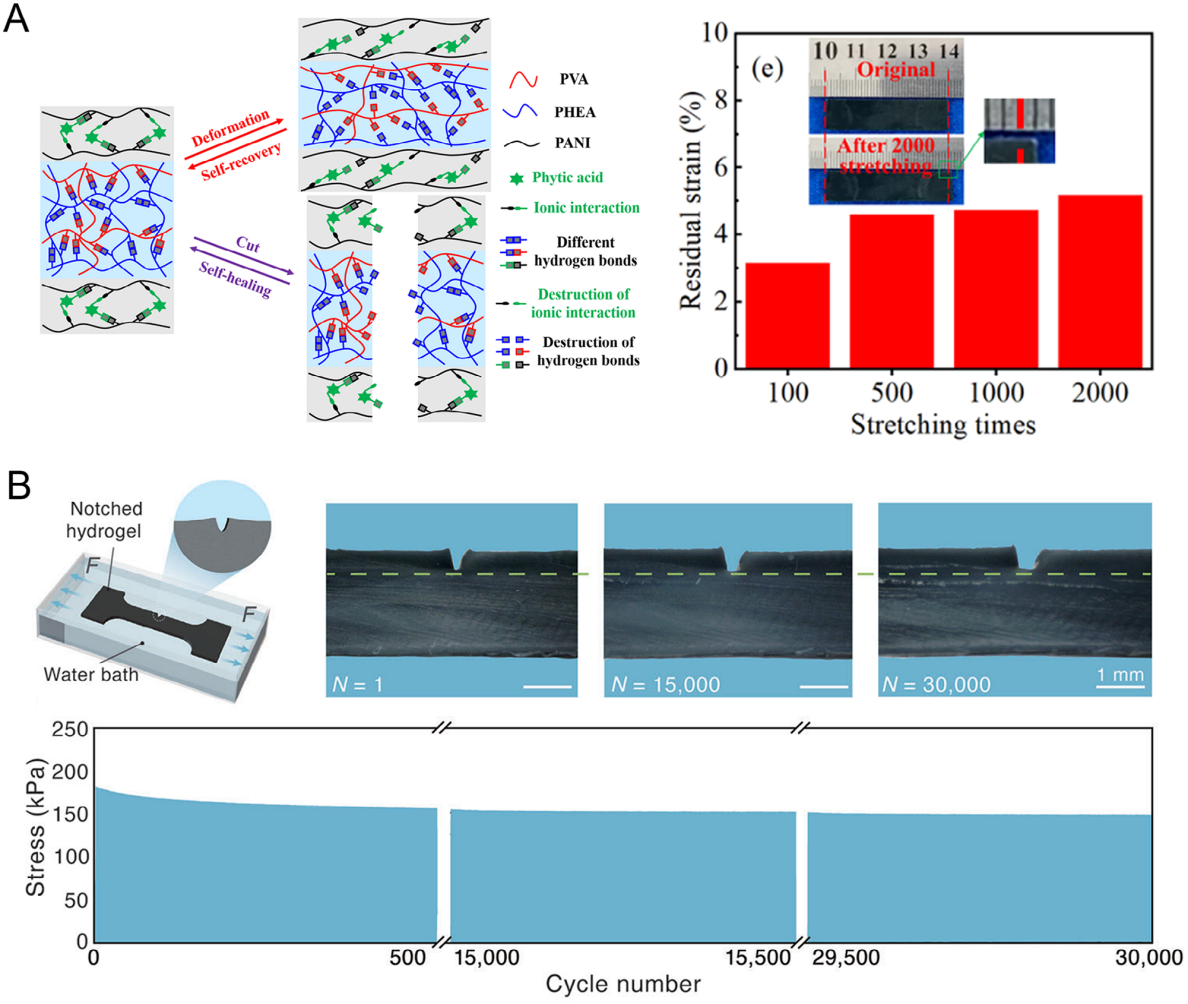
The fatigue resistance of SCPHs. (A) Schematic mechanism of anti‐fatigue and self‐repair of PANI‐modified PVA/PHEA hydrogels and results of anti‐fatigue experiments. Reproduced with permission.^[^ ^132^ ^]^ Copyright 2023, Wiley‐VCH. Copyright 2020, American Chemical Society. (B) Single notch tensile test of PVA/PEDOT: PSS and tensile photos after 30,000 cycles. Reproduced with permission.^[^ ^24^ ^]^ Copyright 2023, Wiley‐VCH.

In recent years, several studies have been reported proposing the introduction of supramolecular interactions into hydrogel networks to prepare hydrogels with excellent fatigue resistance. Similarly, introducing supramolecular interactions into SCPHs networks is a well‐established method to improve fatigue resistance. For example, Sun et al. used in situ polymerization to introduce polyaniline onto the surface of poly (vinyl alcohol)/poly (n ‐hydroxyethyl acrylamide) (PVA/PHEA) hydrogels to form supramolecular hydrogels with an integrated sandwich structure.^[^ ^132^ ^]^ In the supramolecular hydrogel structure, PANI was crosslinked through hydrogen and ionic bonds formed between PANI and phytic acid, and PVA/PHEA was closely linked through hydrogen bonding. The PANI‐modified PVA/PHEA hydrogels exhibited exceptional fatigue resistance due to the multiple effective interactions within the polymer networks. Under cyclic stretching, the supramolecular interactions within the hydrogel experienced reversible fracture and reorganization, leading to a hydrogel that exhibited negligible changes in residual strain and length after stretching (Figure 14A). Furthermore, designing hydrogels with preferentially aligned micro/nanostructures can effectively enhance fatigue resistance. For example, Liu et al. prepared a PVA/PEDOT: PSS hydrogel with a directionally aligned microstructure, combining directional freezing and salting‐out treatment.^[^ ^24^ ^]^ By utilizing ice crystals as templates, the hydrogel formed a directionally aligned structure by extending the growth direction of ice. Salt precipitation modulated the hydrogen bonding between PVA and the electrostatic interactions between PEDOT: PSS, inducing phase separation and crystallization of SCPHs, and providing a toughening effect. Thanks to its unique ordered nanofiber structure and high crystallinity, the PEDOT: PSS‐PVA conductive polymer hydrogel exhibited excellent mechanical properties and could reach a fatigue threshold of more than 300 J m^−1^ in parallel orientation. The absence of crack expansion during 30,000 consecutive cyclic tensile cycles demonstrated excellent fatigue resistance (Figure 14B).

In conclusion, SCPHs with good fatigue resistance are crucial for the reliability and stability of sensors. By introducing and modulating supramolecular interactions in the polymer network, the fatigue resistance of SCPHs can be effectively improved. Furthermore, it is important to acknowledge that certain hurdles and unresolved matters about fatigue resistance research exist. For instance, sensors often operate in intricate and ever‐changing environments, wherein factors such as temperature, humidity, and chemical substances may influence them. Consequently, it becomes imperative to address the challenge of preserving the fatigue resistance of SCPHs under diverse environmental conditions.

## 4 SCPH WEARABLE SENSOR

### 4.1 Preparation of SCPH sensor

The SCPHs sensor typically comprises three main components: the SCPHs, sensor assembly, and signal detection device. Among these, the SCPHs serve as the primary signal‐sensing element, making it the central component of the sensor. The preparation and molding methods employed play a crucial role in developing hydrogel sensors. Various molding techniques impact the structure and performance of these sensors. For instance, by carefully controlling the molding process, one can regulate the microstructure and surface morphology of the hydrogel, thereby achieving desired levels of sensitivity, selectivity, and stability in the sensor. Moreover, fabricating the hydrogel sensors into specific patterns makes it possible to monitor different spatial distribution regions and enable multi‐channel detection, thereby enhancing the efficiency and accuracy of detection. This review focuses on three commonly employed methods for fabricating SCPH sensors: casting molding, 3D printing, and in situ molding.

#### 4.1.1 Casting molding

**FIGURE 15:**
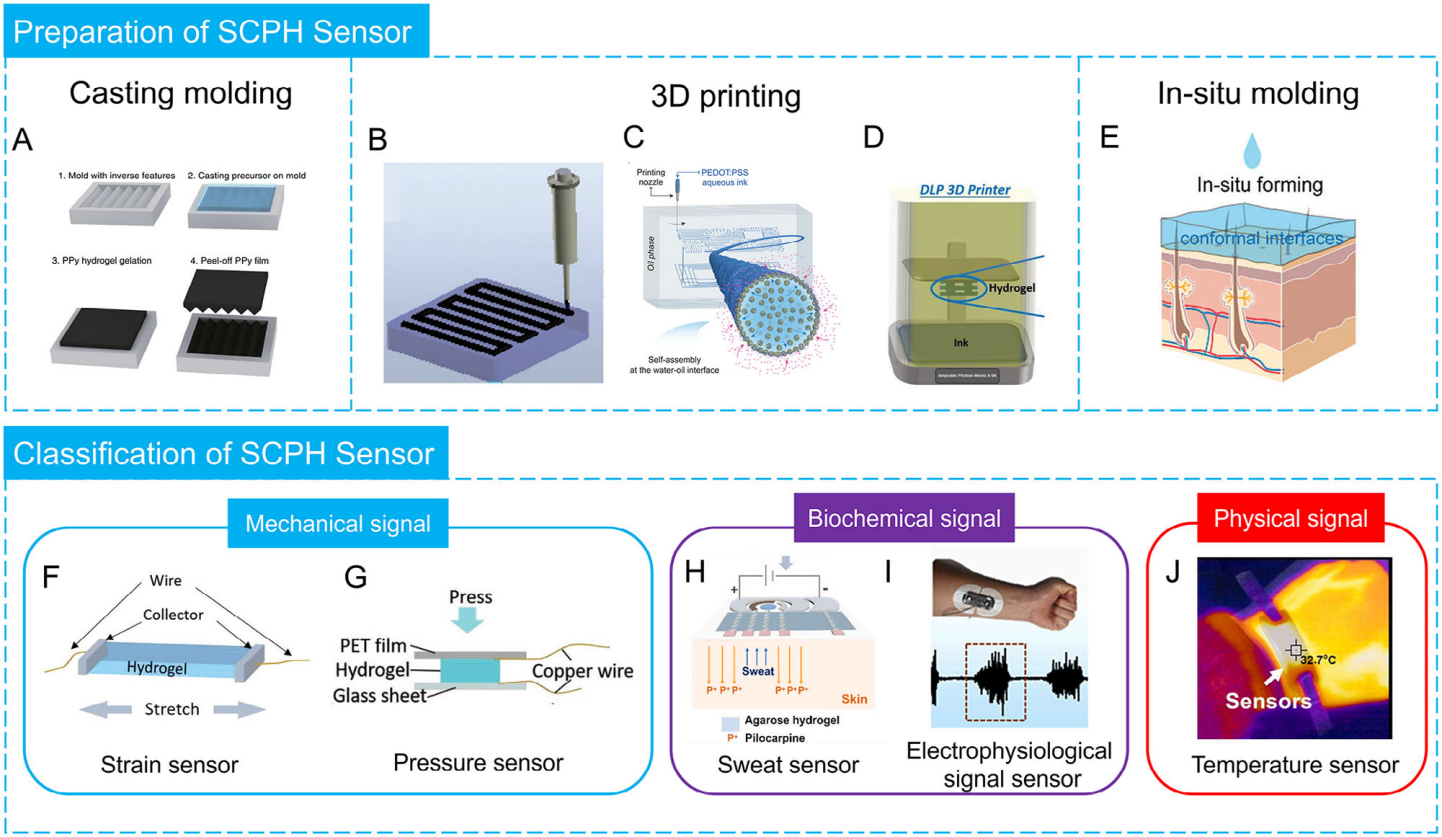
Common fabrication methods for SCPH sensors: (A) Cast molding, Reproduced with permission.^[^ ^133^ ^]^ Copyright 2014, Springer Nature. (B) Direct‐write 3D printing. Reproduced with permission.^[^ ^134^ ^]^ Copyright 2017, Wiley‐VCH. (C) Liquid‐in‐liquid 3D printing. Reproduced with permission.^[^ ^135^ ^]^ Copyright 2023, Wiley‐VCH. (D) light‐cured 3D printing. Reproduced with permission.^[^ ^136^ ^]^ Copyright 2023, Wiley‐VCH. (E) In situ molding. Reproduced with permission.^[^ ^137^ ^]^ Copyright 2023, Wiley‐VCH. Classification of SCPHs: (F) Strain Sensors. (G) Pressure sensors. Reproduced with permission.^[^ ^99^ ^]^ Copyright 2022, Wiley‐VCH. (H) Sweat sensor. Reproduced with permission.^[^ ^138^ ^]^ Copyright 2023, Elsevier. (I) Electrophysiologic signal sensors. Reproduced with permission.^[^ ^76^ ^]^ Copyright 2022, Elsevier. (J) Temperature sensors. Reproduced with permission.^[^ ^139^ ^]^ Copyright 2022, Elsevier.

Casting molding, a widely employed method in SCPH molding, offers numerous advantages in the fabrication of SCPH sensors. This technique involves pouring a precursor liquid of SCPHs into a mold, followed by appropriate curing conditions, yielding SCPHs with distinct shapes and sizes (Figure 15A).^[^ ^133^ ^]^ The versatility of casting molding enables the customization of intricate SCPH shapes, allowing for the design of hydrogels with varying dimensions, such as thin films, fibers,^[^ ^129^ ^]^ and blocks, catering to specific requirements. Furthermore, this method facilitates the preparation of hydrogels with different structures and patterns.^[^ ^65^ ^]^ One notable advantage of casting molding is the consistent shape and size of the resulting hydrogels, ensuring sensor stability and repeatability. Moreover, the SCPHs produced through this technique can undergo subsequent processing, such as removing unreacted by‐products, thereby enhancing their purity. Additionally, casting and molding enable large‐scale production, making it suitable for mass manufacturing. For successful implementation of this molding approach, the SCPHs precursor liquid should possess adequate fluidity and moldability, allowing for easy mold filling and desired shaping. Simultaneously, the precursor fluid must exhibit sufficient curability to ensure the formation of a stable hydrogel structure upon curing within the mold.

#### 4.1.2 3D printing

In recent years, the remarkable advancements in 3D printing technology have sparked a surge of interest in its application to prepare SCPHs.^[^ ^140^ ^]^ Various 3D printing methods have been explored for fabricating SCPH sensors, including direct‐write 3D printing (Figure 15B),^[^ ^134^ ^]^ liquid‐in‐liquid 3D printing (Figure 15C),^[^ ^135^ ^]^ and light‐curing 3D printing (Figure 15D).^[^ ^136^ ^]^ Among these methods, extrusion 3D printing has emerged as a popular choice due to its ease of operation and adjustability.^[^ ^141^ ^]^

Extrusion 3D printing involves the controlled layer‐by‐layer extrusion of SCPH ink to construct the desired sensor structure.^[^ ^142^ ^]^ This technique offers several advantages in the preparation of SCPH sensors. Firstly, it enables the flexibility to design and fabricate sensors of diverse shapes and sizes, thanks to its precise control and customizable manufacturing capabilities. Secondly, extrusion 3D printing allows for creating complex structures, including hollow and multilayer configurations. Moreover, this method facilitates the integration of different functional materials through multi‐material printing, thereby enhancing the performance and functionality of SCPH sensors. Nevertheless, the successful implementation of extrusion 3D printing for SCPH sensors relies on certain requirements for the SCPH inks. Firstly, the ink must possess appropriate viscosity and rheological properties to ensure smooth flow within the extrusion head and maintain shape stability during printing. Secondly, the conductive fillers within the ink need to be uniformly dispersed to guarantee good conductivity in the printed conductive polymer hydrogel. Additionally, the ink's curing speed should be moderate to ensure the stability of the printing process and the quality of the final printed structure.

#### 4.1.3 In situ molding

In situ molding represents a groundbreaking technique for fabricating conductive hydrogel sensors, which can be seamlessly shaped directly on the skin or various sensing substrates (Figure 15E).^[^ ^137^ ^]^ This method utilizes a hydrogel precursor liquid as a reaction feedstock to generate conductive hydrogel structures by in situ polymerization. The inherent advantage of in‐situ molding lies in its ability to shape the hydrogel precisely at designated locations and materials, tailoring it to the sensor's specific requirements. Moreover, this approach facilitates a robust bond between the hydrogel and the substrate, thereby enhancing the stability and reliability of the sensor. To meet the demands of hydrogel ink formulation for in situ molding, it is imperative to employ inks possessing a specific viscosity and fluidity, ensuring their uniform coating or injection onto the substrate during the polymerization process, ultimately yielding a homogeneous hydrogel structure. Furthermore, the ink must possess an appropriate polymerization rate and stability to ensure the timely and optimal completion of the polymerization reaction. Additionally, the ink composition necessitates careful consideration of the material's biocompatibility and stability, guaranteeing the safety and reliability of the hydrogel sensor during usage. Consequently, meticulous selection and design of suitable ink formulations are indispensable for successfully preparing conductive hydrogel sensors via in situ molding techniques.

Various preparation and molding methods, and patterning techniques, have greatly expanded the application potential of conductive hydrogel sensors, offering a wider range of choices and enhanced flexibility for the design and fabrication of such sensors. Additional components such as the collector, encapsulation material, and signal detection device are incorporated into the prepared SCPHs to create a fully functional sensor. The collector directs the current transmission path, ensuring precise current delivery to the sensing element and detection device. Moreover, the encapsulation material is crucial in shielding the conductive hydrogel sensor from external environmental disturbances while providing mechanical support and immobilization.

### 4.2 Classification of SCPH sensor

Recent studies have demonstrated the immense potential of SCPH sensors across a broad spectrum of applications. These sensors can sense diverse signals, enabling them to perform various functions. To facilitate a better understanding of these sensors, we have categorized conductive hydrogel sensors into three distinct groups based on the nature of the signals they detect: mechanical, physical, and biochemical signals. Mechanical signals pertain to deformations or pressures exerted on objects. Among these, strain/pressure sensors are the most prevalent, capable of sensing and converting mechanical strain or force into electrical signals. Such sensors find applications in electronic skin, medical devices, and motion tracking. Physical signals encompass parameters like temperature and humidity in the environment. Lastly, biochemical signals involve signals associated with living organisms, including gases,^[^ ^143^ ^]^ sweat,^[^ ^144^ ^]^ and electrophysiological signals.^[^ ^145^ ^]^ Sweat sensors can monitor sweat composition and secretion in the human body, which is vital for health monitoring and exercise analysis. Electrophysiological signal sensors, such as electrocardiograms (ECG) and electroencephalograms (EEG), can detect the electrical activity of the human body, and are widely employed in medical diagnosis and brain‐computer interface applications.

#### 4.2.1 Strain/pressure sensors

SCPH strain/pressure sensors employ the versatile SCPHs as electrode materials affixed to the surface of living organisms, enabling the susceptible detection of strain and pressure (Figure 15 F,G).^[^ ^99^, ^146^ ^]^ These sensors accomplish this by perceiving the strain and pressure signals and converting them into discernible electrical signals, such as electric current, resistance, or capacitance. SCPH strain/pressure sensors are typically fabricated by encapsulating a SCPH with two electrode materials. The electrode materials connect an external power supply's positive and negative terminal, while the encapsulation materials encase the sensing element, preventing water loss and dehydration of the SCPHs. In this process, the electrical conductivity, mechanical adaptability, and adhesion of SCPHs play a pivotal role. It can conform to the contour of the organism's surface, ensuring a snug fit between the sensor and the organism, thereby providing more accurate strain and pressure signals.

In the case of strain sensors, when an external force is applied to the sensor, the conductive polymer chains of the SCPHs exhibit rapid response and undergo a change in alignment, subsequently influencing the movement path of carriers within the gel network. Specifically, the polymer network elongates along the stretching direction when the sensor is stretched, resulting in longer transport paths for carriers, including electrons and ions, within the gel. Consequently, this increases the resistance of the hydrogel. For SCPHs, the rigid conductive polymer establishes a stable connection with the pliable hydrogel matrix under slight strain. At this stage, the conductive pathways within the gel remain intact and continuous, leading to a smaller change in resistance. However, under significant strains, as the hydrogel network elongates, the conductive polymer slips, and the hydrogel polymer network separates, causing an interrupted conductive path and a substantial change in resistance.^[^ ^147^ ^]^ Regarding pressure sensors, the internal microstructure of the SCPHs undergoes alteration when external pressure is applied, resulting in modifications to the charge transfer path and impedance.^[^ ^148^ ^]^ At low pressures, the reduction in contact resistance leads to a sharp increase in current, reflected in the high sensitivity observed in the early relative current versus pressure curve. Conversely, at high‐intensity pressures, achieving complete contact with the conducting polymer inside the SCPHs no longer contributes to sensitivity, leading decreased sensitivity.

Evaluating the performance metrics of conductive hydrogel strain sensors is of utmost importance in ensuring their accurate, reliable, and stable operation.^[^ ^149^, ^150^, ^151^ ^]^ The key factors are sensitivity, linearity, and stability. Sensitivity, often quantified by the gauge factor (GF), reflects the sensor's ability to respond to strain and pressure, establishing the relationship between changes in the sensor's output signal and strain variations.^[^ ^152^, ^153^ ^]^ A high sensitivity indicates the sensor's capability to detect the slightest alterations in strain and pressure, enhancing measurement precision and accuracy. Linearity measures the degree of correlation between the sensor's output signal and strain/pressure. A sensor with excellent linearity delivers accurate output signals regardless of strain and pressure levels. Stability refers to the sensor's consistency and reliability in providing output signals over prolonged periods of use. A well‐stabilized sensor exhibits minimal drift and fluctuations, ensuring reliable, and consistent measurement results.

Leveraging the sensitivity of SCPHs to strain and pressure, these sensors can be tailored to capture strain and pressure conditions within various contexts, generating precise electrical signals. SCPH sensors possess notable advantages, including high sensitivity, rapid response, and robust reliability, making them versatile across diverse fields such as medical and healthcare, sports training, robotics, and virtual reality.

#### 4.2.2 Sweat sensor

A sweat sensor, an innovative device, is a remarkable tool for measuring and monitoring the human sweat's intricate composition and distinctive characteristics (Figure 15H).^[^ ^138^ ^]^ By analyzing the intricate chemical compounds, ions, and other biomolecules present in sweat, this technology enables the extraction of valuable insights regarding an individual's physical well‐being, exercise regime, and metabolic levels. At the core of sweat sensors lies the principle of electrochemical sensing, harnessing electrochemical reactions to facilitate the detection and analysis of specific target substances. In this context, electrodes, commonly composed of the highly versatile material SCPHs, play a pivotal role as the sensing elements. SCPHs, with exceptional electrical conductivity and biocompatibility, ensure seamless contact with sweat and facilitate the stable transmission of electrical signals. Furthermore, the integration of conductive hydrogels, either as electrode materials or as carriers for immobilizing reactants, serves to augment the electrochemical reaction efficacy, thereby enhancing the sensitivity and stability of sweat sensors. In practice, SCP sweat sensors are typically fabricated by integrating SCPHs into a three‐electrode system, alongside a microfluidic device, while the non‐contacting side of the sensor is encapsulated with a specialized material. Incorporating a microfluidic device, meticulously designed to regulate the flow of minute liquids, precisely directs the sweat into the designated working area of the sensor, ensuring optimal performance and accuracy.^[^ ^154^ ^]^

The SCPH sweat sensor operates through two primary mechanisms: collection and analysis. Initially, sweat is gathered using a microfluidic array or absorbent material seamlessly integrated into the sensor. Subsequently, the collected sweat sample interfaces with the sensor's working electrode for the subsequent analysis step. An electrochemical reaction ensues upon interaction between the sweat's analytes and the electrode surface, generating distinct current or potential signals. These signals exhibit a positive correlation with the concentration of the targeted substance. The concentration of the desired substance can be ascertained by gauging the alteration in the current or potential signal. Several pivotal metrics warrant consideration when evaluating conductive SCPH sweat sensors. Firstly, the SCPH sweat sensor should exhibit heightened sensitivity, accurately discerning minute changes in sweat's trace components. Secondly, the SCPH sensor should selectively detect specific components while remaining unaffected by other interfering substances. Moreover, response time assumes significance as a key metric, with a swift response time ensuring the sensor's ability to promptly capture changes in sweat composition. In conclusion, sweat sensors, employing electrochemical sensing technology and SCPHs as crucial constituents, can monitor sweat composition in real‐time and non‐invasively.

#### 4.2.3 Electrophysiological signal sensor

An electrophysiological signal sensor is a remarkable tool for detecting and recording the electrical activity of living organisms (Figure 15I).^[^ ^76^ ^]^ It captures and registers electrical signals generated within tissues such as neurons, muscles, and the heart, subsequently transforming them into comprehensible electrical outputs. Electrophysiological signaling sensors for various application scenarios are commonly produced using distinct fabrication methods. For instance, sensors designed for electromyography (EMG) and ECG detection typically involve connecting the electrophysiological signaling sensor to an electrode patch. On the other hand, EEG sensors are affixed to an EEG cap, while ophthalmology sensors are fabricated as a headband, enabling efficient detection. The underlying principle of electrophysiological signal sensors lies in their ability to detect the subtle currents and potential fluctuations arising from electrical activity in living organisms. In the case of electrically active cells and tissues, such as neurons, muscle cells, and cardiomyocytes, the generation and transmission of bioelectrical signals heavily rely on the selective transport of ions across cell membranes, accompanied by corresponding changes in membrane potential.^[^ ^155^ ^]^ Generally, the recording or modeling of electrical activity in excitable tissues involves the conversion of electrical signals between ions and electrons. At the interface between tissues and electrons, a state of equilibrium is maintained through electrolyte‐electrode interactions, encompassing ion diffusion, redox reactions, and bilayer effects. This equilibrium ensures a relatively stable potential. However, when there is a shift in ion flux, a changing potential arises and influences the movement of electrons within the circuit. By placing the sensor in contact with the tissues of a living organism, it becomes capable of capturing these electrical signals and converting them into voltage or current signals, which can then be amplified and processed.

Traditional sensor electrode materials, namely metals and semiconductors, fail to align with the mechanical properties of biological tissues due to their inherent rigidity, leading to inadequate electrode‐tissue surface interaction, elevated contact impedance, and suboptimal signal coupling. On the contrary, using SCPH presents numerous advantages for electrophysiological signal sensors. Firstly, these conductive polymer hydrogels exhibit exceptional biocompatibility, ensuring seamless integration with biological tissues devoid of significant irritation or damage. Secondly, they possess commendable electrical conductivity, effectively facilitating the transmission of weak electrical signals within the organism, thereby enhancing the sensor's sensitivity and precision. Additionally, the inherent flexibility and adaptability of conductive polymer hydrogels enable them to conform to biological tissues’ diverse shapes and curvatures, ensuring an impeccable fit and unwavering stability. Consequently, electrophysiological signal sensors based on SCPHs hold immense potential for a broad spectrum of applications in neuroscience, cardiology, biomedical engineering, and beyond, furnishing us with an invaluable tool and avenue for comprehending the intricate electrical activities intrinsic to living organisms.

#### 4.2.4 Temperature sensor

A temperature sensor, an essential tool for monitoring temperature fluctuations in living organisms and the surrounding environment, operates by detecting alterations in electrical resistance (Figure 15J).^[^ ^139^ ^]^ The conventional approach employed for fabricating SCPH temperature sensors involves the integration of the SCPHs with the collector, forming a two‐electrode sensor through the utilization of an encapsulating material. This encapsulating material exhibits a low thermal conductivity and serves as an insulating layer, thereby safeguarding the temperature sensor against thermal energy dissipation. When the temperature rises, the sensor achieves temperature measurement by monitoring the resistance change of the SCPHs in real time. Compared to traditional temperature sensors, SCPH sensors exhibit many advantages. Conventional temperature sensors are primarily constructed on rigid substrates, restricting their stretchability and detection range. Conversely, SCPH sensors demonstrate exceptional flexibility and malleability, enabling effortless customization into diverse forms and sizes to cater to varying application needs. Additionally, SCPH sensors boast remarkable electrical conductivity, facilitating rapid and precise temperature measurements characterized by swift response times and exceptional accuracy.

In SCPH temperature sensors, the conductive polymer chain segments serve as conductive channels, facilitating the movement and conduction of carriers on their surfaces. As the temperature rises, the movability of the polymer chain segments increases, leading to enhanced carrier transportation and a subsequent decrease in resistance.^[^ ^139^, ^156^ ^]^ Furthermore, conductive ions within the conductive polymer hydrogel material can migrate through the hydrogel network. With increasing temperature, the molecular motion within the hydrogel accelerates, resulting in faster ion migration and increased carrier density, leading to a rapid decrease in resistance. Thus, SCPH temperature sensors effectively reflect temperature changes by measuring resistance variations.

An important performance indicator for evaluating temperature sensors is the temperature coefficient of resistance (TCR), which quantifies the resistance change rate in response to temperature variations.^[^ ^157^, ^158^, ^159^ ^]^ A higher TCR value indicates greater sensitivity to temperature changes, resulting in higher temperature resolution. In practical applications, SCPH temperature sensors demonstrate a high TCR value, making them ideal for temperature detection. They can be widely employed for sensing human body temperature, object surface temperature, and external temperature, providing a reliable solution for temperature monitoring and control across diverse fields.

## 5 APPLICATION OF SCPHS IN WEARABLE SENSORS

In the previous section, we categorized SCPHs based on the signals detected by the sensors and introduced the sensing mechanisms and evaluation metrics of the sensors. In this section, we further discuss the applications of these SCPH sensors from different application scenarios. Here, we summarize the main application scenarios of SCPH sensors, including motion sensing, sweat analysis, temperature sensing, electrophysiological signal monitoring, human–computer interaction, soft robotics sensing, and information transmission.

### 5.1 Motion perception

Motion sensing is a method that utilizes sensors and technical devices to record and analyze human movement processes. It involves monitoring various movement indicators, including heart rate, pulse, facial micro‐expression changes, vocal cords, and joint movements. These sensors provide accurate and reliable exercise data, which supports athletes and coaches in scientifically optimizing training and making adjustments to improve performance and prevent sports injuries. Moreover, motion sensing is crucial in assessing an individual's health status, enabling better health management and maintenance. SCPH sensors are particularly important in sports monitoring and health detection. These sensors leverage the properties of SCPHs to adhere closely to the surface of human skin, enabling non‐invasive motion monitoring. They can sense and analyze motion in real time in different environments and strain windows, making them valuable for rehabilitation training, motion assessment, and postural monitoring.

**FIGURE 16:**
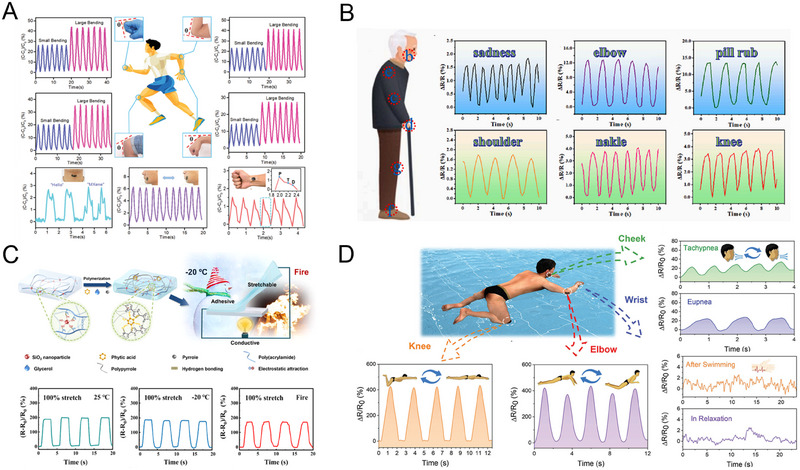
SCPH sensors for motion perception. (A) Examples of PVA@MXene@PPy hydrogel sensors for monitoring human motion. Reproduced with permission.^[^ ^160^ ^]^ Copyright 2023, Elsevier. (B) PANI NPs@(CS‐PAA) hydrogel sensor for monitoring Parkinsonian behavior. Reproduced with permission.^[^ ^61^ ^]^ Copyright 2023, Wiley‐VCH. (C) Schematic synthesis mechanism of SCPHs with anti‐freezing and flame‐retardant properties and their strain sensing properties at low and high temperatures. Reproduced with permission.^[^ ^161^ ^]^ Copyright 2022, American Chemical Society. (D) Application of PAM‐ALG‐PPy hydrogel sensor for underwater breaststroke detection. Reproduced with permission.^[^ ^100^ ^]^ Copyright 2023, Wiley‐VCH.

The skin undergoes stretching or contraction due to mechanical stimuli (e.g., pressure and strain) resulting from human movement. This in turn induces geometric deformation of the SCPH sensors attached to the skin, leading to the generation of real‐time electrical signals in response to human motion.^[^ ^149^, ^150^, ^151^ ^]^ Leveraging this mechanism, SCPH sensors can be attached to different body parts to monitor human activity. Xu's team prepared a PMP conductive hydrogel utilizing poly(vinyl alcohol) (PVA), Ti_3_C_2_T_x_ nanosheets, and PPy as constituent materials.^[^ ^160^ ^]^ By introducing the multifunctional crosslinkers iron ions and borax into the PMP hydrogel, a variety of dynamic interactions, including dynamic borate, hydrogen bonds, and coordination bonds, were constructed, giving the hydrogel excellent tensile properties, ultra‐high toughness, and self‐healing properties. The PMP hydrogel‐based sensors accurately detected physiological signals across a broad strain window. They were capable of capturing joint movements that involved substantial deformations, as well as weak deformations like facial expressions and pulses (Figure 16A).

Moreover, exercise monitoring holds the potential to facilitate early detection of certain disease indicators, thereby empowering individuals to take timely action. Notably, neurological disorders like Parkinson's Disease (PD) and Alzheimer's Disease can significantly impair an individual's motor functions, leading to symptoms such as muscle rigidity, tremors, and abnormal gait patterns. Gu et al. developed a SCPH sensor consisting of polyaniline (PANI) and polyacrylic acid‐chitosan (PAA‐CS) to monitor somatic tremors in PD patients.^[^ ^61^ ^]^ Due to the hydrogen bonding between the PANI and PAA‐CS hydrogel matrices, the hydrogel's mechanical properties and electrical conductivity were greatly enhanced. When utilized as a versatile sensor in a simulated PD patient, it demonstrated the capability to monitor electrical signals from multiple body parts, serving as a discerning factor in the early detection of PD symptoms (Figure 16B).

However, wearable sensors are often subjected to different usage scenarios in their daily operations, including extreme low and high temperatures. Hence, it is necessary to develop SCPH sensors capable of functioning effectively in such harsh environments. Tang and colleagues prepared a frost‐resistant and flame‐retardant SCPH sensor consisting of covalently crosslinked PAm and PA‐doped PPy (Figure 16C).^[^ ^161^ ^]^ The introduction of PA facilitated the crosslinking of PPy via hydrogen bonding or electrostatic interactions. Moreover, PA exhibited remarkable flame‐retardant properties, allowing the sensor to perform well even after burning. Meanwhile, due to introducing an antifreeze agent, glycerol, into the SCPHs system, the hydrogel possessed excellent freezing resistance, exhibiting excellent deformability and toughness maintained at −20°C. As a result, the hydrogel sensor exhibits stable and consistent sensing behavior for human motion in both hot and cold environments. Han et al. adopted a similar strategy to prepare a low‐temperature resistant hydrogel by introducing glycerol into the PVA and PEDOT: PSS hydrogel system.^[^ ^162^ ^]^ Incorporating glycerol resulted in hydrogen bonds between the PVA molecular chains and the water molecules within the hydrogel, thereby imparting water retention and anti‐freezing properties to the hydrogel. Alternatively, Du et al. presented PAM/PEDOT: PSS/CNF SCPHs with excellent electrical conductivity and antifreeze properties.^[^ ^163^ ^]^ Introducing LiCl into these SCPHs enhanced the interaction between the hydrogel and the water molecules, effectively inhibiting the formation of ice crystals at low temperatures. Such hydrogel sensors could sensitively monitor various human movements, including walking and running.

The sensors mentioned above function effectively in arid terrestrial conditions. However, wearable sensors are bound to encounter diverse moist environments, including human perspiration, natural precipitation, and underwater settings during swimming. Hence, it becomes crucial to devise a SCPH‐based sensor that can be deployed in humid surroundings. Huang's team prepared a multifunctional amphiphilic hydrogel sensor by introducing various supramolecular interactions, including coordination bonds, electrostatic interactions, and cation‐π into PPy‐PAM‐ALG hydrogel networks. Thanks to the formation of coordination bonds and the hydrophobicity of PPy, SCPHs effectively prevented continuous water infiltration, exhibiting remarkable anti‐swelling properties and exceptional stability underwater. In addition to monitoring complex human movements on land, the hydrogel sensor was successfully employed to monitor breaststroke and underwater communication (Figure 16D).^[^ ^100^ ^]^

The versatility of SCPHs makes them an ideal motion sensor platform for a wide range of applications in motion sensing and health monitoring. Their remarkable flexibility and stretchability enable them to conform to a wide range of wearable parts components and accommodate complex deformations. This feature not only enhances wearer comfort but also improves the monitoring accuracy. The high electrical conductivity of the sensors allows for highly sensitive strain detection, making them ideal for capturing subtle movements during physical activity. In addition, the SCPH sensors exhibit excellent environmental adaptability and remarkable stability, enabling them to function reliably in a wide range of complex environments, including high or low temperatures, on land or underwater. Therefore, developing SCPH sensors provides a more convenient and accurate tool for exercise monitoring technology, providing better support for health management and exercise training.

### 5.2 Healthcare monitoring

#### 5.2.1 Sweat analysis

Sweat is an important reflection of the body's internal environment, containing a wealth of physiological and metabolic information. By monitoring the biochemical components and parameters in sweat, such as electrolytes,^[^ ^164^, ^165^ ^]^ glucose,^[^ ^166^, ^167^ ^]^ lactate^[^ ^168^, ^169^ ^]^, etc., people can obtain critical information about their physical health and exercise status for health management, disease diagnosis and prevention. Sweat monitoring presents the advantages of being non‐invasive, real‐time and convenient.^[^ ^170^, ^171^ ^]^ Traditional sweat sensing materials often have problems such as low sensitivity, poor stability, and slow response time. As a new functional material with multifunctionality, SCPHs provide new ideas and solutions to solve these problems. In recent years, researchers have applied SCPHs in sweat sensing and achieved a series of impressive results.

**FIGURE 17:**
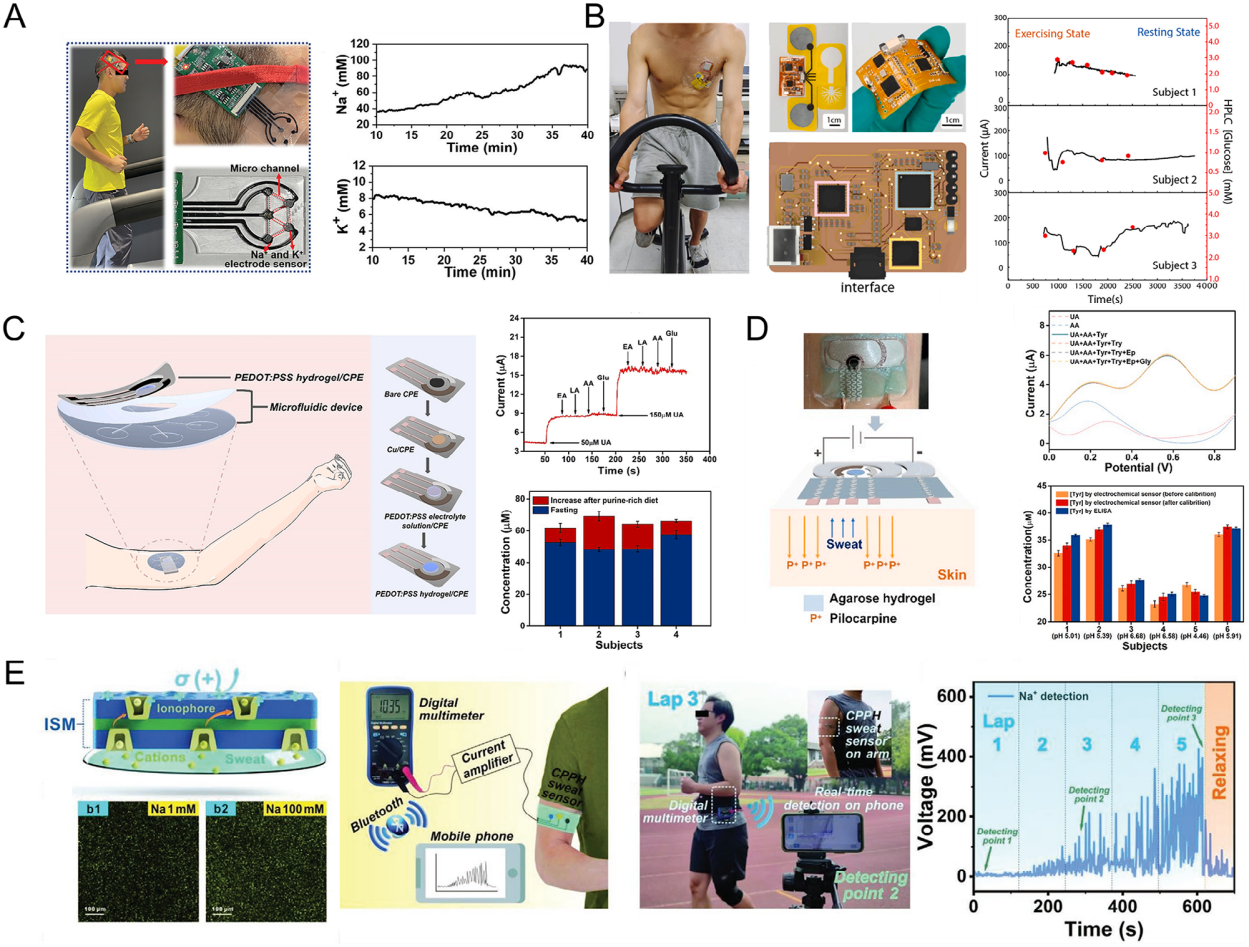
SCPH sensors for sweat analysis. (A) Real‐time analysis of K^+^ and Na^+^ in sweat by SCPH sensors during running. Reproduced with permission.^[^ ^172^ ^]^ Copyright 2021, Royal Society of Chemistry. (B) Photograph of the integrated SCPHs sweat sensor and real‐time on‐body sweat sensing. Reproduced with permission.^[^ ^173^ ^]^ Copyright 2022, Elsevier. (C) Schematic structure of a SCPH sensor integrated with a microfluidic device and its performance in detecting uric acid in sweat. Reproduced with permission.^[^ ^154^ ^]^ Copyright 2021, Elsevier. (D) Schematic diagram of the working principle of SCPHs sweat sensor and detection of tyrosine in human sweat by the sensor. Reproduced with permission.^[^ ^138^ ^]^ Copyright 2023, Elsevier. (E) Schematic diagram of the operation of a self‐powered sweat sensor and real‐time monitoring of ions in sweat by a wireless sensor worn on the human body. Reproduced with permission.^[^ ^175^ ^]^ Copyright 2022, Wiley‐VCH.

Ions are vital components in sweat, with the concentrations of potassium and sodium ions being significant for monitoring the body's hydration status during exercise. In order to achieve accurate detection of these two ions, Yu et al. fabricated a sweat sensor by integrating a PAA‐PEDOT hydrogel onto a flexible printed circuit board.^[^ ^172^ ^]^ By detecting the changes in open‐circuit voltage, the sensor selectively detected different potassium and sodium ion concentrations. Such sensors attached to the human body could monitor K^+^ and Na^+^ in sweat during exercise in real‐time (Figure 17A). Furthermore, the glucose level in sweat is an important indicator for assessing the fatigue level of athletes. Ye and coworkers designed a multifunctional flexible hydrogel‐paper patch (HPP) to detect glucose levels by self‐assembling PEDOT: PSS hydrogel on paper fibers.^[^ ^173^ ^]^ The spontaneous capillary effect allowed the paper patch to serve as a microfluidic channel for efficient collection and analysis of sweat. In practical applications, the HPP could be integrated into a multifunctional wearable device for real‐time detection of glucose metabolism as well as electrocardiographic data during exercise in different volunteers (Figure 17B).

Apart from the health state during exercise, specific components of human metabolic sweat can offer valuable insights into disease conditions. For instance, uric acid in human sweat is the end product of purine metabolism and is associated with cardiovascular and renal disease. It has also been utilized as a diagnostic marker for gout.^[^ ^174^ ^]^ Wang et al. designed a microfluidic‐based electrochemical sensor by integrating a PEDOT: PSS hydrogel onto a microfluidic electrode.^[^ ^154^ ^]^ Based on capillary action, microfluidic sensors on human skin collected sweat produced by the epidermis in real time. The PEDOT: PSS hydrogel electrode with a large surface area selectively recognized and detected the electrochemical signal of uric acid without interference from other components of sweat. Furthermore, the sensor detected uric acid levels in the real sweat of volunteers after a purine‐containing diet (Figure 17C). This sensor could track uric acid in human sweat in real time, with important implications for reducing the risk of gout and hyperuricemia. Moreover, acid tyrosine in human sweat is a marker associated with several diseases, such as tyrosinemia and bulimia nervosa. Luo et al. developed a wearable sensor system integrating a sweat‐promoting IP system and an electrochemical sensor based on a tannic acid‐silver‐carbon nanotube‐polyaniline hydrogel.^[^ ^138^ ^]^ The composite hydrogel was very sensitive to pH. When pH changes, PANI undergoes deprotonation of H^+^, resulting in a change in open‐circuit voltage. In addition, the composite hydrogel possessed excellent electrocatalytic activity for the oxidation of complexine, which could selectively monitor the electrochemical oxidation peaks of complexine (Figure 17D). Thus, sensor patches based on this hydrogel could simultaneously detect the pH range of human sweat as well as the concentration of complexine in sweat.

While SCPHs have made significant progress in sweat sensing, they still encounter several challenges and issues. One challenge is the need for self‐healing properties in the electrode material, leading to a decrease in its lifespan. Furthermore, the power supply method employed for sweat sensors is unreliable and necessitates prolonged reliance on an external power source. These limitations further restrict the potential application of SCPHs in wireless sweat sensing. Nie and colleagues prepared a wearable self‐powered sweat sensor consisting of cellulose‐based SCPH electrodes for real‐time monitoring of ion concentration in sweat during exercise.^[^ ^175^ ^]^ In order to meet the complex deformations encountered during human movement, they introduced the borax quadruple hydrogen bonding system into SCPHs, producing SCPHs with highly stretchable, self‐healing properties. Self‐powered flexible sweat sensors can be created by assembling a PDMS‐encapsulated flexible SCPH electrode as a negative friction material and an ion‐selective membrane as a positive friction material (Figure 17E). Through ion permeability induced changes in charge density on the surface of an ion‐selective membrane, a self‐powered sweat sensor based on the friction electric effect can quantify and analyze sodium, potassium, and calcium ions in sweat in real time, transmitting the signals wirelessly to a user interface. This versatile sweat sensor offers new ideas for health monitoring and wearable AI devices.

SCPHs, as a significant electrode material, present a promising solution for sweat monitoring. Its large specific surface area, excellent electrical conductivity and high structural stability enable effective and consistent contact with perspiration, ensuring a stable current transfer path over time. Combining conductive hydrogels with advanced sensing technologies makes it possible to realize highly sensitive and selective monitoring of various components and parameters in sweat. The research outcomes can be widely applied in individualized health management and exercise monitoring, disease diagnosis, drug efficacy assessment, and physiological state monitoring.

#### 5.2.2 Electrophysiological signal monitoring

Electrophysiologic signals are electrical signals that originate from different physiological activities within the body. These signals serve a crucial role in the functioning of the nervous system, heart, muscles, and other tissues.^[^ ^176^, ^177^, ^178^, ^179^ ^]^ By deeply understanding the electrical activities inside the human body, people can better understand the physiological functions of the human body and disease mechanisms, providing a scientific basis for health management and disease treatment.^[^ ^180^, ^181^, ^182^, ^183^ ^]^ Recently, to realize the monitoring of electrophysiological signals, researchers have successfully developed a series of wearable devices for electrophysiological signal monitoring using SCPHs. These devices integrate several properties of SCPHs, including high electrical conductivity, flexibility, and biocompatibility. As a result, they can be conveniently attached to the body's surface to enable real‐time monitoring of changes in electrophysiological signals.

**FIGURE 18:**
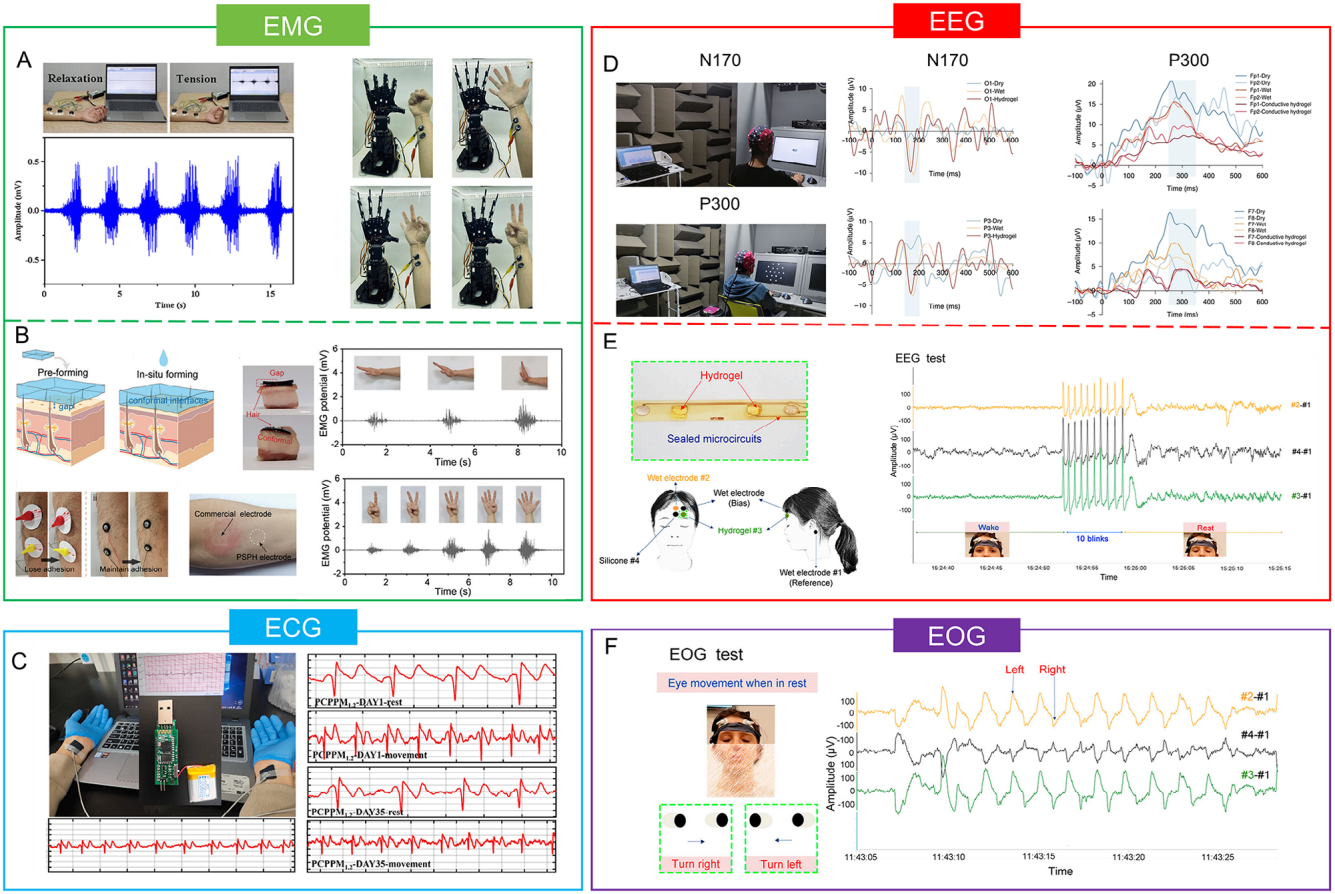
SCPH sensors for electrophysiological signal monitoring. (A) The Gel/PPy/rGO sensors are used to control robotic arms by capturing and recognizing human EMG signals. Reproduced with permission.^[^ ^184^ ^]^ Copyright 2022, Elsevier. (B) In situ formed PSP hydrogel sensors detect EMG signals on complex body surfaces (hairy areas). Reproduced with permission.^[^ ^137^ ^]^ Copyright 2023, Wiley‐VCH. (C) Photographs of PCPPM hydrogel sensors and real‐time monitoring of ECG signals by the sensors. Reproduced with permission.^[^ ^185^ ^]^ Copyright 2021, Elsevier. (D) N170 and P300 testing with dry, wet and SCPH electrodes via EEG caps. Reproduced with permission.^[^ ^139^ ^]^ Copyright 2023, Springer Nature. (E‐F) Hydrogel‐integrated wearable BMI for EEG (E) and EOG monitoring (F). Reproduced with permission.^[^ ^186^ ^]^ Copyright 2023, Wiley‐VCH.

Electrophysiological signals generated by muscles, such as electromyography (EMG), provide valuable information to assess muscle contraction and relaxation. This information is crucial for research in rehabilitation medicine, sports training, and human–computer interaction. For instance, Wang's team successfully developed a multifunctional organic hydrogel based on gelatin/polypyrrole/reduced graphene oxide (Gel/PPy/rGO).^[^ ^184^ ^]^ The organic hydrogel was capable of accurately and stably collecting and recognizing the characteristic signals of myoelectricity and cardiac electricity and served as an interface for human–computer interaction to manipulate the manipulator by recording the myoelectric signals (Figure 18A).

However, conventional hydrogel electrodes face challenges when fitting tightly onto the skin due to factors such as skin folds and hair. This can result in a high contact resistance, negatively impacting signal transmission quality. To address this, Jia et al. proposed a strategy to prepare SCPHs using PEDOT: PSS to promote spontaneous polymerization of SBMA.^[^ ^137^ ^]^ These SCPHs were spontaneously polymerized in situ on the skin to obtain the ISF‐PSPH electrode and formed a tight contact with the skin unaffected by hair (Figure 18B). Compared to commercially available electrodes, the ISF‐PSPH electrodes exhibited superior performance in capturing EMG signals, boasting higher signal‐to‐noise ratios, and demonstrating the ability to accurately recognize EMG signals associated with various gestures. Importantly, the ISF‐PSPH electrodes fitted well to the skin and accurately detected EMG signals even in areas with hair. Additionally, the ISF‐PSPH electrodes were antimicrobial and biocompatible, ensuring that volunteers did not experience any harm to their skin from prolonged contact with the hydrogel electrodes.

Electrophysiologic signals originating from the heart, such as the electrocardiogram (ECG), reveal the rhythm and function of the heart. These signals are critical for diagnosing and treating various heart diseases. Yu et al. produced a hydrogel PCPPM based on hydrogen and coordination bonds by homogeneously dispersing PEDOT: PSS into PVA and CMC hydrogel matrices.^[^ ^185^ ^]^ The hydrogel had excellent electrochemical properties, outstanding biocompatibility, and soft mechanical properties, ensuring that the PCPPM seamlessly contacts the skin with significantly reduced contact impedance. By integrating PCPPM hydrogel electrodes with a commercially available ECG development module to fabricate a sensor, they accurately responded to the human heart beating under relaxation and exercise. They detected the characteristic waveform of the heart beating (Figure 18C). Tan et al. prepared a C10P5 hydrogel based on hydrogen bonding and host‐guest interaction using chitosan, water‐soluble PPy, and cucurbit as materials.^[^ ^76^ ^]^ Compared to commercially available electrodes, the C10P5 hydrogel electrode, with lower impedance, was more favorable for ECG signal monitoring, exhibiting higher sensitivity for ECG detection. Under high‐speed running conditions, commercial gel electrodes were unable to obtain accurate ECG signals, whereas the C1095 hydrogel could still accurately monitor ECG signals.

Brain‐machine interfaces (BMI) enable direct interaction between the human brain and artificial devices by connecting the human brain to external devices. BMI is available in noninvasive and implantable types and can detect a wide range of brain signals. For example, Lin et al. prepared a noninvasive brain‐machine interface electrode for electroencephalogram (EEG) monitoring by introducing a conductive polymer into a covalent‐ionic double crosslinked PAM‐SA hydrogel system.^[^ ^139^ ^]^ Multiple interactions (ligand bonding, hydrogen bonding, electrostatic interactions) present in the network of SCPHs endowed the SCPHs electrode with good robustness. In addition, SCPHs are characterized by low impedance and high biocompatibility, ensuring that the hydrogel electrodes can obtain high quality EEG signals. In both N170 and P300 tests, it was demonstrated that hydrogel electrodes successfully detected EEG signals at the microvolt level and effectively captured event‐related units, showing negative peaks at 170 and 300 ms (Figure 18D). In addition, the recorded signals exhibited power spectra similar to those obtained using wet electrodes.

Similarly, Lu et al. prepared ultrasoft BMI with mechanical properties matching those of brain tissues by introducing hydrophilic PEDOT nanoparticles into a carrageenan‐polydopamine‐polyacrylamide interpenetrating network.^[^ ^186^ ^]^ In addition, since SCPHs were rich in catechol molecules that could form various noncovalent and covalent bonds with brain tissues, such SCPHs could conformally adhere to skin tissues and brain tissues with folded structures. As a result, BMIs integrated by SCPHs can be seamlessly attached to the surface of the cerebral cortex to obtain high‐fidelity intracranial electrocorticography (ECoG). Furthermore, SCPHs and headbands can be combined to capture EEG in both awake and resting states (Figure 18E). The BMI captures EEG signals superior to dry electrodes and similar to commercially available wet electrodes but with better stability than commercially available wet electrodes. In addition, this BMI can be monitored with electrooculography (EOG) (Figure 18F). Due to the BMI's excellent adhesion and conductivity, it accurately captures eye movement signals with better signal quality than commercially available electrodes, which effectively reduces interface resistance.

SCPHs play a vital role in monitoring electrophysiologic signals. Its high degree of plasticity, sensitivity, mechanical properties similar to human tissues, excellent biocompatibility, and superior adhesion enable it to reliably and accurately capture and transmit electrical signals from within living organisms. This capability provides valuable data and information to physicians and researchers. By monitoring and analyzing these electrophysiological signals, people can better understand the body's physiological state and detect and prevent potential health problems.

### 5.3 Temperature monitoring

Temperature is a ubiquitous physical quantity that significantly impacts human life. Monitoring temperature allows us to comprehend and regulate environmental conditions, ensuring a comfortable and safe living environment.^[^ ^187^, ^188^, ^189^ ^]^ Moreover, temperature monitoring holds significant importance in the medical field, as it allows healthcare professionals to monitor changes in a patient's body temperature, detect temperature abnormalities, and promptly address them to protect their health.^[^ ^190^, ^191^, ^192^ ^]^ With fast response, high sensitivity, and reusability advantages, SCPHs have been widely used in environmental and human body temperature monitoring.

**FIGURE 19:**
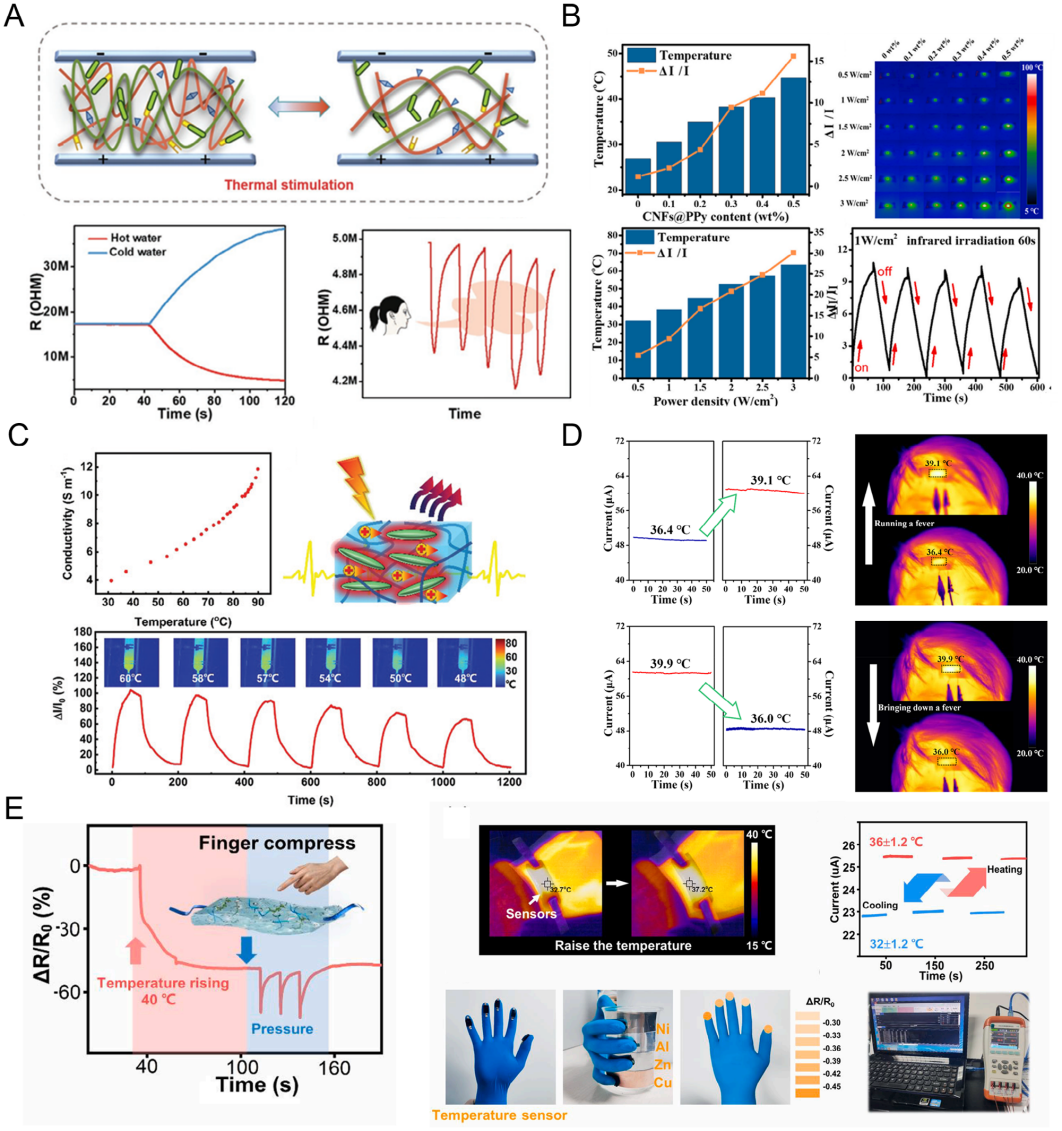
SCPH sensors for temperature monitoring. (A) Schematic diagram of the temperature response principle of SCPH sensors and sensing of the temperature of cold and hot water and breathing gases. Reproduced with permission.^[^ ^156^ ^]^ Copyright 2021, Wiley‐VCH. (B) Near‐infrared absorption and photothermal response of PHEMA/PVA/CNFs@PPy hydrogel. Reproduced with permission.^[^ ^62^ ^]^ Copyright 2023, Elsevier. (C) The relationship between conductivity and temperature of PPFe4 hydrogels with photothermal conversion properties and the hydrogel response to water at different temperatures. Reproduced with permission.^[^ ^193^ ^]^ Copyright 2020, Royal Society of Chemistry. (D) PANI NFs temperature sensor serves as a “fever indicator” to monitor a human forehead's temperature. Reproduced with permission.^[^ ^194^ ^]^ Copyright 2020, American Chemical Society. (E) The PBA/CPA/Gly hydrogel sensors possess the ability to simultaneously monitor strain and temperature, and there are examples of utilizing the sensors to recognize temperature. Reproduced with permission.^[^ ^139^ ^]^ Copyright 2022, Elsevier.

Regarding temperature sensors, SCPHs can be employed as a sensing element to monitor and measure temperature through changes in its structure or properties. For instance, Li and coworkers designed a versatile hydrogel sensor based on electrostatic interactions.^[^ ^156^ ^]^ Since the degree of movement of the polymer chains and the mode of ion transfer changed when the temperature was altered, this hydrogel sensor could sensitively capture changes in different temperatures, transforming the temperature change into a linear electrical signal. The hydrogel sensor detected electrical signals for both hot and cold water, and exhibited repetitive and stable electrical signal changes in response to changes in circulating temperature. In addition, utilizing this temperature sensitivity of the device, the sensor detected temperature changes in the gas produced when the body exhales (Figure 19A).

There is growing evidence that conductive polymers, including PEDOT, PPy, and PANI, have significant coefficients of thermal expansion. By introducing these conductive polymers, the temperature resolution of the material can be significantly improved. For example, Wang et al. developed a strain/photothermal multi‐responsive PHEMA/PVA/ CNFs@PPy hydrogel sensor based on the synergistic effect of hydrogen and coordination bonds.^[^ ^62^ ^]^ Owing to the photothermal properties of CNFs@PPy nanofibers, the hydrogel was heated to a higher temperature under the stimulation of near‐infrared light, which accelerated the migration of conductive ions and facilitated the rapid carrier migration (Figure 19B). Thus, the hydrogel sensor received an increased current as the temperature increased. Moreover, hydrogel sensors have demonstrated successful applications in monitoring real‐time temperature changes on the human body and the surface of objects, such as a hot water cup. Alternatively, Zhang et al. prepared a SCPH sensor based on hydrogen and coordination bonding by mixing PVA grafted with phytic acid, pyrrole, and Fe^3+^.^[^ ^193^ ^]^ Benefiting from the extensive absorption of light by the black PPy, the sensor showed excellent photothermal conversion. When irradiated by near‐infrared lasers of different powers and different time durations, the sensor's surface temperature and conductivity increased. The hydrogel, with a linear response to temperature, could be employed as a thermal sensor to monitor the real‐time temperature of hot water at different temperatures by attaching it to the outer wall of a glass tube (Figure 19C). The temperature coefficient of resistance (TCR) is the rate of change in the resistance value of a sensing material as the temperature changes. Higher TCR values indicate that the temperature sensor responds more sensitively to temperature changes, allowing higher temperature resolution. Fast thermal responsiveness and high accuracy temperature resolution are critical for temperature detection in practical applications. Dong et al. developed a multifunctional hydrogel sensor by introducing reversible coordination bonds, hydrogen bonds and electrostatic interactions in PAA and PANI nanofibers (PANI NFs) binary mesh hydrogels.^[^ ^194^ ^]^ Benefiting from the thermal sensitivity of PANI NFs, the sensor possessed excellent thermal responsiveness (TCR = 1.64%/°C), demonstrating outstanding temperature discrimination. The hydrogel sensor has been successfully used as a “heat indicator” for human forehead temperature detection, displaying a highly recognizable temperature resolution (2.7°C) (Figure 19D).

Although most SCPHs exhibit excellent thermal responsiveness, the currently available thermal sensors face limitations in achieving multimodal detection, making it difficult to effectively recognize strain and temperature‐superimposed signals without interference. Yang et al. developed a SCPH sensor capable of simultaneously detecting strain and temperature using hydrogen bonding and electrostatic interactions.^[^ ^139^ ^]^ Synchronized monitoring of motion and temperature was achieved by coupling charge transfer induced by spatial position with tunneling currents activated by thermal vibrations. The obtained hydrogels exhibited excellent temperature responsiveness (TCR = 2.01%/°C) and strain sensitivity due to the superimposed signal recognition ability of CPs. In practical temperature detection, the sensor adhered to the wrist can monitor the body temperature in real time. Additionally, an array of temperature sensors assembled by SCPHs can detect and differentiate between different temperatures (Figure 19E).

The temperature sensors prepared by SCPHs possess highly sensitive and accurate temperature sensing capabilities in temperature sensing. As the conductivity of the SCPH material changes in response to temperature variations, generated electrical signals can be measured and analyzed to obtain precise temperature readings. In addition, its soft nature allows it to be in close contact with skin or other objects to monitor temperature changes in real time. CMSPH, as a new type of temperature sensing material, has broad application prospects in environmental and human body temperature monitoring, providing a more accurate and convenient solution for temperature monitoring.

### 5.4 Human–machine interaction

Human–machine interaction devices encompass information exchange and interaction between individuals and computer/machine systems.^[^ ^195^, ^196^ ^]^ As science and technology continue to advance and people increasingly seek enhanced intelligence and convenience, the significance of human–computer interaction grows ever more pronounced. Traditional sensors typically rely on electronic components or mechanical structures, often exhibiting limitations in their ability to perceive the environment and users. In contrast, SCPH sensors offer a range of advantages, including flexibility, deformability, sensitivity, accuracy, reliability, and durability. These attributes enable a more personalized, comfortable, and precise human–computer interaction experience, providing exceptional input and feedback. Consequently, incorporating SCPH sensors holds immense potential across numerous human–computer interaction scenarios, elevating both user experience and system performance to excellence.

**FIGURE 20:**
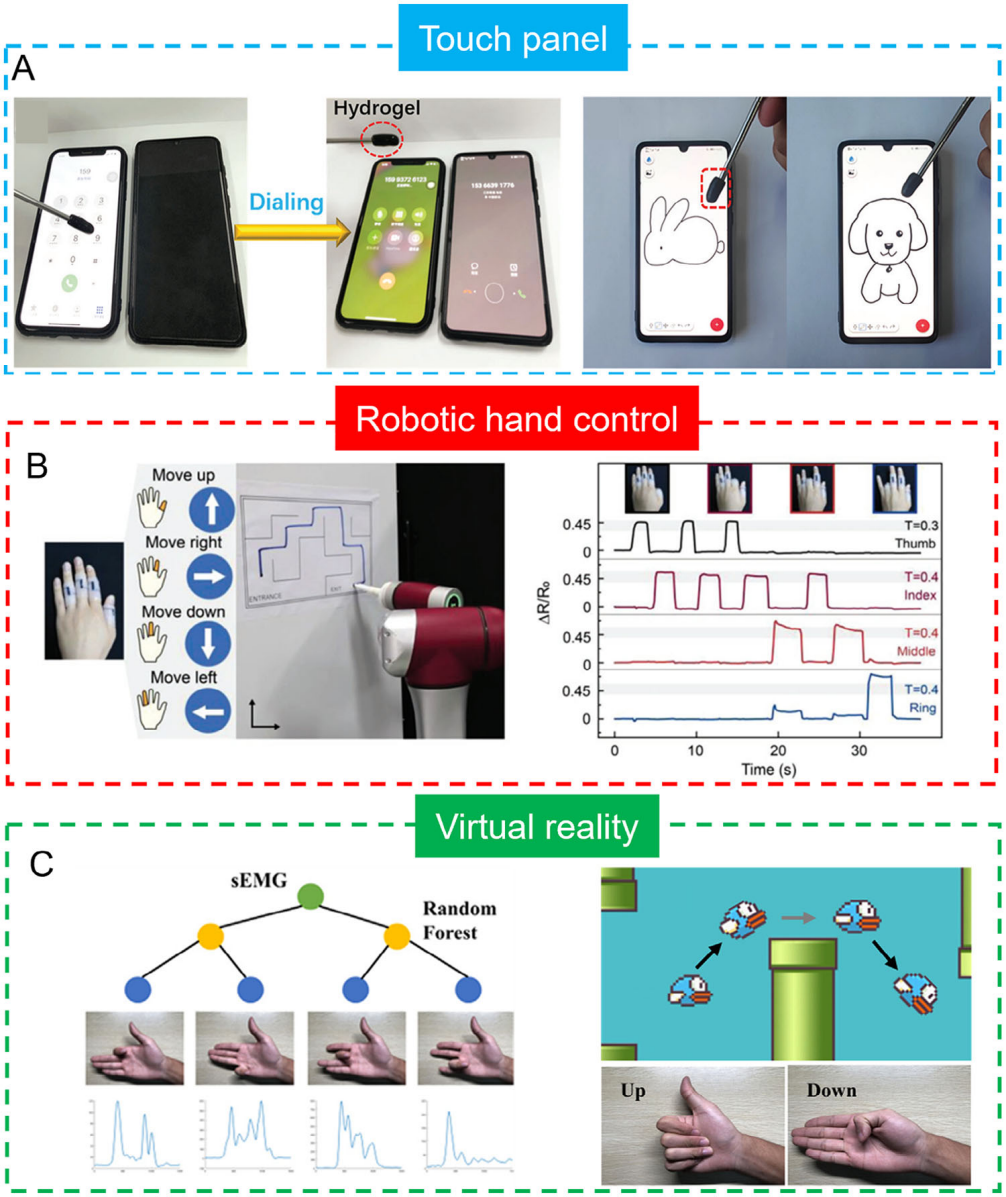
SCPH sensors for human‐machine interaction. (A) SCPHs integrate the touch screen pen for dialing and drawing.^[^ ^160^ ^]^ Copyright 2023, Wiley‐VCH. (B) PEDOT: PSS‐PVA hydrogel remotely controls a robotic arm to execute different movement paths. Reproduced with permission.^[^ ^41^ ^]^ Copyright 2022, Wiley‐VCH. (C) SCPH sensors are employed for virtual character control by capturing EMG signals. Reproduced with permission.^[^ ^184^ ^]^ Copyright 2023, Elsevier.

Deformable and highly sensitive SCPH sensors hold great potential for their application in touch screen technology. For example, Xu et al. demonstrated the development of an exceptionally resilient conductive hydrogel, comprising PVA, Mxene, and PPy. This hydrogel was an ideal conductive material and could be easily integrated into an electronic stylus for seamless smartphone interaction, enabling activities such as dialing numbers and drawing (Figure 20A).^[^ ^160^ ^]^ Furthermore, SCPH sensors have played a pivotal role in scenarios involving remotely manipulated robotic arms, enabling the monitoring of the operator's hand movements and strength to achieve precise and flexible control. Gu et al. had devised a PEDOT: PSS‐PVA hydrogel sensor for remote robot control.^[^ ^41^ ^]^ By assigning distinct action paths to the bending motion of each finger, sensors worn on different fingers could command the robot to execute preset motion trajectories (Figure 20B). This capability showcased the sensor's ability to perform multi‐channel tasks, simultaneously monitoring and relaying the movements of all five fingers to the robot control system. Similarly, Zhai and colleagues had developed a PEDOT: PSS/PVA hydrogel capable of manipulating a robotic arm.^[^ ^197^ ^]^ Hydrogel sensors were affixed to various body parts, such as the fingers, elbows, and shoulders, and paired with the robotic arm's Bluetooth module through an interaction sensor. As these body parts moved, the sensors received electrical signals and promptly transmitted them to the robotic arm, enabling the appropriate action to be performed. Moreover, hydrogel sensors have also found utility in virtual and augmented reality technologies. Wang et al. devised a smart EMG wristband with SCPHs that accurately recognized different gestures by capturing EMG signals in real time.^[^ ^184^ ^]^ This precise gesture recognition has empowered users to manipulate virtual characters seamlessly and instantaneously (Figure 20C).

In conclusion, SCPH sensors find extensive application in a diverse array of human–machine interface domains, encompassing touch screens, robotic arm control, and virtual reality. Integrating hydrogel sensors into these interfaces holds immense potential for revolutionizing human–computer interaction, offering heightened sensitivity, flexibility, and multimodal capabilities. This advancement facilitates precise gesture recognition and motion control, fostering a more natural and intuitive user experience. Moreover, hydrogel sensors’ inherent flexibility and adaptability enable their integration across various devices and scenarios, allowing users a more convenient and comfortable interaction. Under the relentless pursuit of innovation and the continued implementation of hydrogel sensor technology, human–computer interaction stands poised to undergo further enhancement, enabling users to engage more intelligently and efficiently in an interaction.

### 5.5 Soft robot sensing

A soft robot is a system built on flexible materials and flexible structures. Compared with traditional hard‐bodied robots, soft‐bodied robots have higher flexibility and plasticity and can adapt to various complex environments and task requirements.^[^ ^198^, ^199^, ^200^ ^]^ SCPH sensors, as a sensing platform with high flexibility and conductivity, provide the necessary support for the sensing and interaction capabilities of soft‐bodied robots. Traditional sensors often require hardware support and fixed installation. In contrast, SCPH sensors can be integrated into the flexible structure of soft‐bodied robots, enabling sensing and interaction with the environment. This flexible sensor platform provides more possibilities for soft robot applications.

**FIGURE 21:**
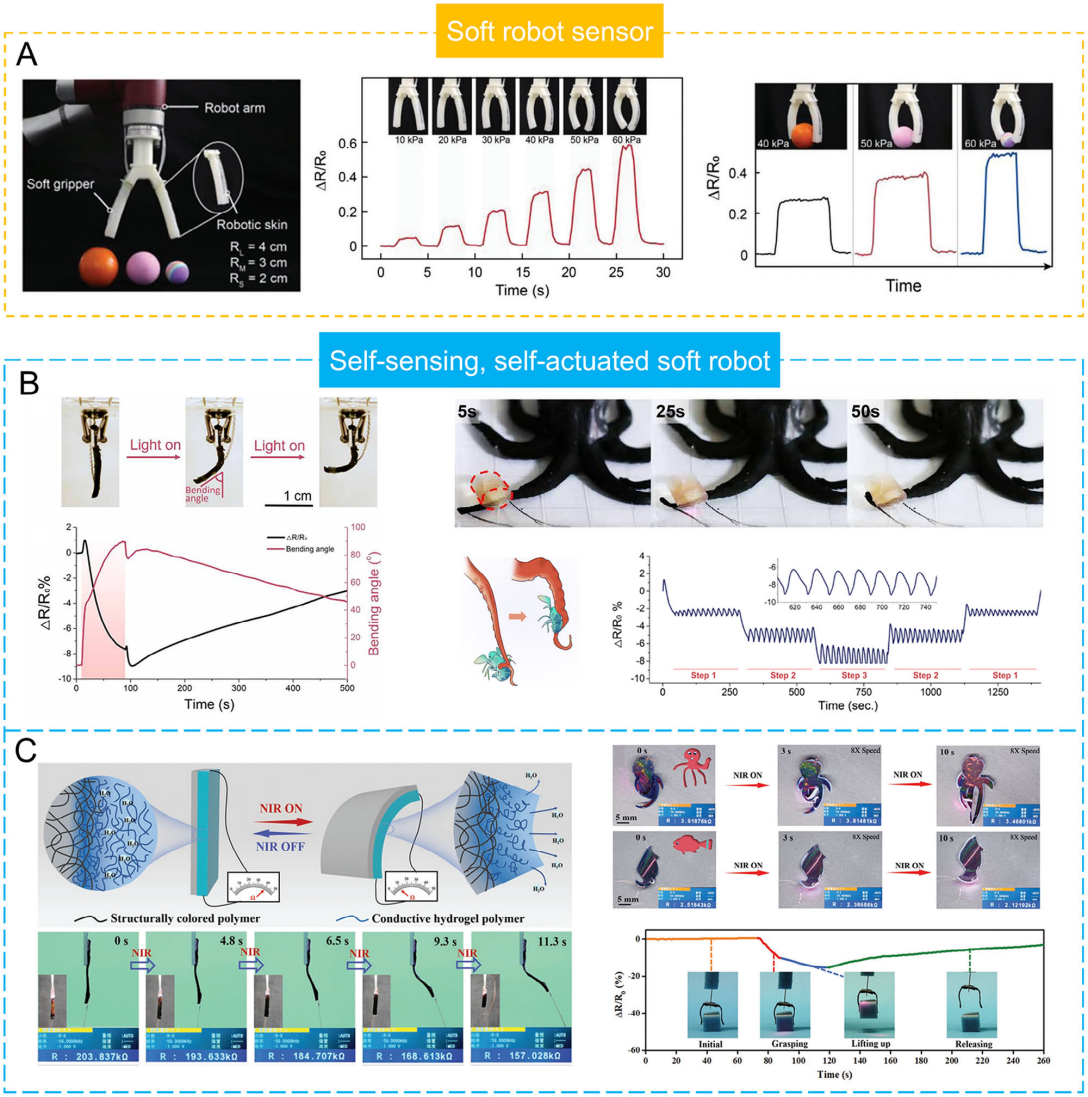
SCPH sensors for soft robot sensing. (A) PEDOT: PSS/PVA hydrogel monitors the process of object grasping by a soft robotic gripper. Reproduced with permission.^[^ ^41^ ^]^ Copyright 2022, Wiley‐VCH. (B) The light‐driven feature of SCPHs and the soft machine octopus developed by SCPHs realize the self‐actuation and sensing of grasping objects. Reproduced with permission.^[^ ^201^ ^]^ Copyright 2021, American Association for the Advancement of Science. (C) The schematic diagram of the programmable shape of the SCPHs actuator, as well as the shape deformation of the SCPHs artificial octopus, fish, and gripper driven by near‐infrared light. Reproduced with permission.^[^ ^202^ ^]^ Copyright 2023, Wiley‐VCH.

SCPH sensors bestow soft robots with perceptual capabilities, greatly enhancing human–robot interaction and enabling the realization of autonomous robotic systems. For instance, Gu et al. devised a 3D printable SCPH strain sensor by combining PEDOT: PSS nanofibers and PVA.^[^ ^41^ ^]^ The exceptional electrical and mechanical properties of SCPHs were achieved by leveraging electrostatic interactions between PSS/PVA and PDEOT, hydrogen bonding among PVA chains, and chain entanglement between PVA and PSS, which fostered a robust connection at the phase interface between the conductive and mechanical phases. Subsequently, the researchers seamlessly integrated the SCPH sensor into a soft machine gripper to monitor the gripping process. By discerning the bending angle of the soft gripper, the sensor indirectly gauges the object's size by detecting strain changes (Figure 21A). Furthermore, the sensor is proficient in recognizing the loading and unloading states of the machine gripper.

However, integrating additional sensing elements into soft actuators presents significant challenges, particularly in adhesion and stiffness matching. Therefore, developing self‐sensing and self‐actuating materials holds excellent importance for soft robots. He et al. employed a one‐pot reaction with the ice template method to fabricate strain‐sensing and fast photo‐thermal responsive composite SCPHs.^[^ ^201^ ^]^ On one hand, when irradiated with near‐infrared light, the SCPHs exhibited spontaneous actuation, resulting in shape change. Simultaneously, the SCPHs functioned as sensors capable of monitoring their actuation. By capitalizing on the inherent unity of sensing and driving functions within the SCPHs, a closed‐loop control system was designed to achieve active braking behavior. The resulting soft robot octopus, constructed using SCPHs, possessed both driving and sensing capabilities. It could adjust the curvature of its curl to accommodate objects of varying sizes, enabling it to “grasp” heavy objects and exhibit environment sensing and active braking behaviors akin to those observed in living organisms (Figure 21B). Similarly, Wang et al. introduced conductive components, K‐MXene and PEDOT: PSS, into a thermo‐responsive poly(*N*‐isopropylacrylamide) (PANIPAM) to develop a SCPH with exceptional responsiveness and strain sensitivity.^[^ ^202^ ^]^ The SCPH was combined with polymers featuring structural colors, successfully designing a body‐sensitive hydrogel actuator capable of photo‐thermal self‐actuation and self‐sensing. In addition, the researchers utilized this actuator to create a variety of programmable shape actuators, including a synthetic octopus, an artificial fish, and a pliable gripper (Figure 21C). These devices could be actuated by near‐infrared light while monitoring their motion.

In summary, SCPH sensors find extensive applications in soft robotics. They offer a means to provide haptic feedback to the robot, enabling it to perceive the intensity and contour of external objects’ touch, thereby facilitating more accurate operation and interaction. Furthermore, the SCPH sensor can also detect alterations in shape, pressure, temperature etc., enabling the robot to achieve adaptive control.

### 5.6 Information transmission

Information transmission holds great significance in human life, facilitating the seamless exchange, sharing, and dissemination of knowledge, enabling individuals to access the information they seek conveniently.^[^ ^203^, ^204^ ^]^ In recent years, researchers have successfully developed a series of wearable communication devices based on strain/pressure sensing by utilizing SCPHs, further advancing the development of information transmission. These devices realize the transmission and recognition of information by detecting signals such as the degree of bending of the human finger, changes in pressure, and the way of writing, providing novel, and convenient communication.

**FIGURE 22:**
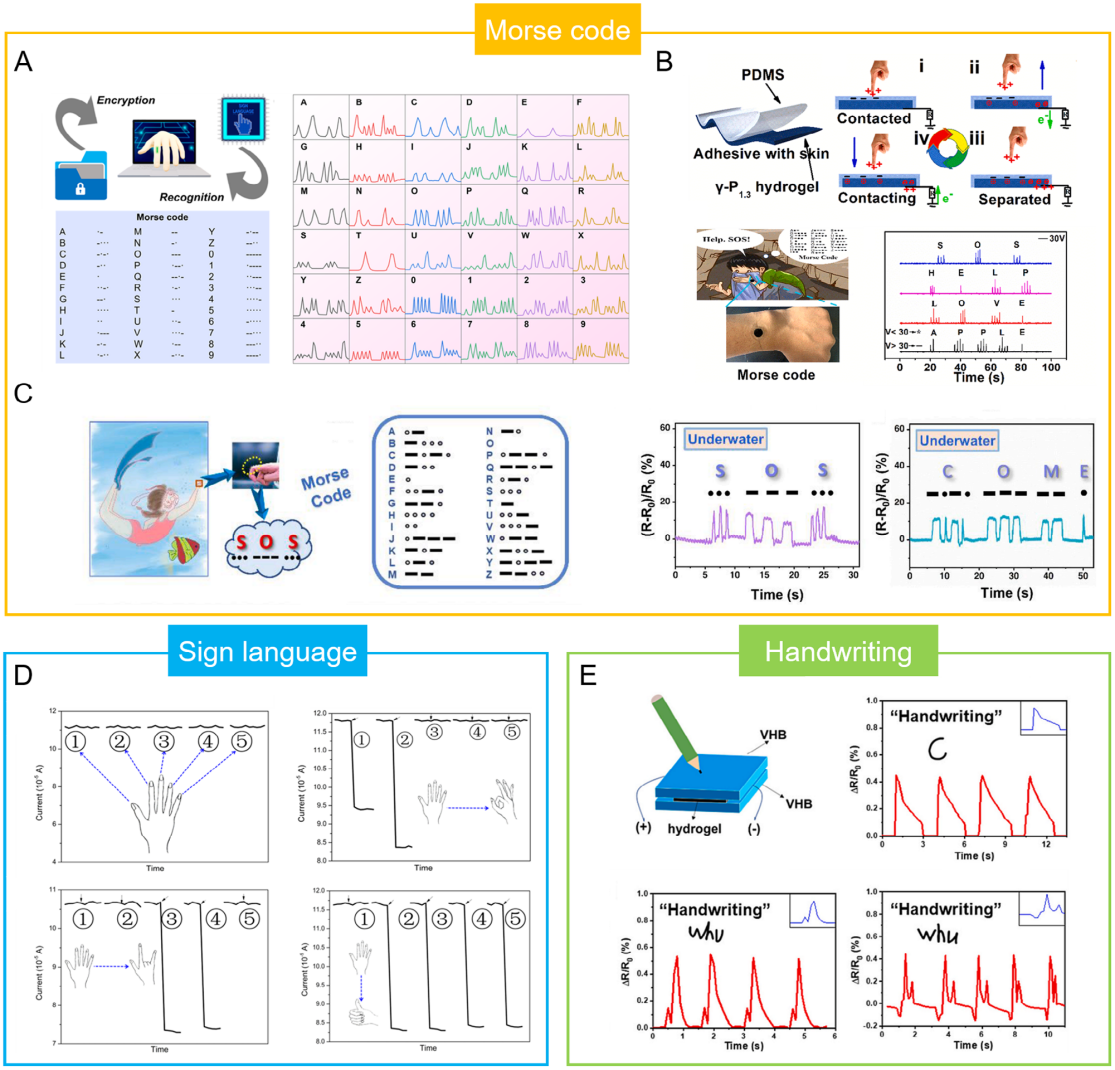
SCPH sensors for information transmission. (A) The sensor transmits finger bending signals into alphabetic and numeric information via Morse code. Reproduced with permission.^[^ ^208^ ^]^ Copyright 2023, American Chemical Society. (B) The SCPH tactile sensors transmit touch signals via Morse code. Reproduced with permission.^[^ ^108^ ^]^ Copyright 2022, Elsevier. (C) The SCPH sensor encrypts and transmits finger bend signals underwater based on Morse code. Reproduced with permission.^[^ ^125^ ^]^ Copyright 2022, Elsevier. (D) The SCPH strain sensors attached to five fingers recognize sign language by monitoring gesture changes. Reproduced with permission.^[^ ^209^ ^]^ Copyright 2023, American Chemical Society. (E) Messaging is based on different individual handwriting habits. Reproduced with permission.^[^ ^89^ ^]^ Copyright 2022, Elsevier.

Morse code is a coding system for transmitting textual information that represents letters, numbers, and punctuation marks through a combination of short and long messages.^[^ ^205^, ^206^, ^207^ ^]^ Inspired by the fact that the bending of the fingers can express short and long messages, the researchers developed a Morse code‐based communication strategy for transmitting information. For example, Uyama et al. developed a symbol‐to‐speech transmission device using functionalized adenine molecules and chitosan‐grafted polyaniline copolymers as materials.^[^ ^208^ ^]^ This device utilizes two different electrical signals output by the small and large bending deformations of the finger, marked with dots and dashed lines as the constituent elements of Morse code (Figure 22A). When a finger is bent regularly according to a predetermined Morse code, the sensor attached to a single finger can detect a specific electrical signal, enabling the encrypted transmission of information. By installing hydrogel sensors at the joints of each finger, specific electrical signals for different gestures can be detected, resulting in the translation and transmission of sign language.

In addition to strain, different degrees of contact patterns can be elements of Morse code. Gao's team introduced multiple hydrogen bonds into γ‐PGA/PEDOT: PSS hydrogels to prepare a SCPH‐based skin contact sensor.^[^ ^108^ ^]^ When the sensor is touched in either a strong or weak way, it can output two different electrical signals. These two signals can be compiled by Morse code to transmit various messages such as “HELP, SOS, LOVE, and APPLE” (Figure 22B). Because of the simple and easily recognizable signals used in Morse code, reliable communication is possible even under harsh environmental conditions, such as at sea, in mountainous areas or on the battlefield. Zou and coworkers constructed a SCPH based on multiple dynamic supramolecular interactions by introducing PPy in combination with filipin (SF) and tannic acid (TA) into the same gel network.^[^ ^125^ ^]^ Since tannins can penetrate the hydration layer and form various non‐covalent bonds with the substrate, the hydrogel can adhere well to the surface of the human body, even in humid environments. Thus, volunteers can transmit messages underwater by flexing their fingers regularly using a preset Morse code (Figure 22C).

In addition, different gestures can represent additional information. The specific gesture information can be obtained by detecting a specific combination of signals in each gesture. By attaching hydrogel to five fingers, the sensor receives different electrical signals after changing gestures due to each finger's diverse bending, thus distinguishing between different gestures. In this way, distinct gestures were realized, such as “OK”, “LOVE”, “PRAISE”, and so on (Figure 22D).^[^ ^209^ ^]^ Moreover, based on individual differences in writing strength and handwriting, the hydrogel sensor can detect and output unique electrical signals for personalized information transfer. Zhou et al. designed a multifunctional sensor by introducing multiple interactions in a SCPH network.^[^ ^89^ ^]^ By inscribing words onto the film‐encapsulated sensor, the sensor could detect and differentiate the handwriting of various individuals, thereby generating distinct electrical signals. This innovative approach held great potential for applications in information anti‐counterfeiting (Figure 22E).

As a multifunctional material, SCPH detects subtle strains and converts them into recognizable information. SCPHs sensor‐based communication strategies enable the transmission of complex and diverse information by discerning elementary actions like gestures, contact patterns, and individual writing styles. The advent of this communication mode has facilitated the transmission of information, enhancing its accessibility, security, and efficiency. This has dramatically benefitted individuals with limited language proficiency or those unable to engage in conventional written communication, offering unparalleled convenience.

Table 3 summarizes the performance of previously reported SCPH sensors, elucidating the variances among these supramolecular approaches, thereby offering a valuable point of reference for the investigation of SCPH sensors.

**TABLE 3:** Performance comparison of SCPHs wearable sensors.

Supramolecular interaction	SCPHs	Sensitivity	Features	Application	Ref.
HB	γ‐PGA/PEDOT: PSS	GF = 1.17 (0–300%) GF = 4.26 (300–550%) GF = 9.89 (550–650%)	Adhesion property Self‐healing Biocompatibility	MP IT	[108]
HB	TOCNF/PANI‐PVAB	SNa^+^ = 0.039, SK^+^ = 0.082, SCa^2+^ = 0.069 mmol^−1^; (Na^+^: 10–100 mmol; K^+^: 1–18.5 mmol; Ca^2+^: 0.41–12.4 mmol)	Self‐healing	SA	[175]
EI	PEDOT: PSS	LOD = 10.3 µm 0−2.5 mm		SA	[173]
EI	PEDOT: PSS / CPE	LOD = 1.2 µmol L^−1^ 2.0–250 µmol L^−1^		SA	[154]
CB	TA‐Ag‐CNT‐PANI	LOD = 3.3 µm 10 µm–200 µm		SA	[138]
HB/CB	PHEMA/PVA/CNFs@PPy	GF = 3.13 (600–1000%)	Self‐healing Adhesive property	MP/IT	[62]
HB/CB	PAM‐ALG‐PPy	GF = 4.1 (0–400%)	Anti‐swelling	MP/IT/ESM	[100]
EI	HPAA/PANI	GF = 2.6 (0–300%) GF = 7.8 (300–1210%) GF = 17.9 (1210–1520%)	Anti‐swelling	MP/IT	[66]
HB/EI	PPy microgels/P(AAm‐*co*‐LMA)/SDS	5.00 (0–400%) 12.37 (400–1000%) 18.40 (1000–1600%)		ESM	[67]
HB/CB/EI	PAA/PANI	GF = 3.93 (0–75.63%) GF = 9.01 (169.04–268.9%) GF = 18.28 (169.04−268.9%) TCR = 1.64%/C 40−110°C	Adhesive property Self‐healing	MP/TS	[194]

Abbreviations: MP, motion perception; IT, information transmission; SA, sweat analysis; ESM, electrophysiological signal monitoring; TS, temperature sensing.

## 6 SUMMARY AND OUTLOOK

This review provides an overview of various supramolecular strategies employed in the development of SCPHs, encompassing hydrogen bonding, electrostatic interactions, host‐guest interactions, and coordination bonds. By incorporating these supramolecular interactions, the intermolecular binding within the polymer chains is significantly enhanced, thereby imparting stability to the hydrogel structure and leading to high‐performance SCPHs. Subsequently, the properties and functionalities of SCPHs relevant to wearable sensor applications are comprehensively discussed. The fine‐tuning of performance and attaining multifunctionality in SCPHs can be achieved through a rational design and selection of supramolecular interactions. Moreover, the review comprehensively elucidates the specific application scenarios of SCPHs in wearable sensors. These applications encompass diverse areas such as motion perception, sweat analysis, temperature sensing, electrophysiological signal monitoring, human–machine interaction, soft robot sensing, and information transmission. Despite the remarkable accomplishments of SCPHs in the sensor field, both opportunities and challenges exist in this promising domain.

Firstly, despite numerous reports mentioning the application of supramolecular strategies in synthesizing SCPHs, the methods for evaluating supramolecular interactions still need improvement. Most of these reports merely offer qualitative descriptions of supramolecular interactions without employing quantitative characterizations. Consequently, there is an urgent need to establish standardized characterization methods and evaluation criteria for studying supramolecular interactions in SCPHs. Such endeavors will provide valuable guidance for developing SCPH sensors with satisfactory sensing performance.

Secondly, the mechanism of supramolecular interactions needs to be further studied and improved. Most existing studies fail to investigate the intricate mechanisms underlying supramolecular interactions, particularly about the formation and dissociation processes. Moreover, the interplay between multiple supramolecular interactions and the impact of external conditions, such as temperature, solvent, and stress, on these interactions, remain largely unexplored. Therefore, it is imperative to delve deeper into these aspects to gain a comprehensive understanding of supramolecular interactions.

Thirdly, promoting the industrialization of SCPHs represents a crucial undertaking. Despite the abundant research achievements on SCPH sensors, the process of industrialization for these sensors remains in its nascent stage, encountering numerous obstacles. On one hand, the intricate preparation process of existing SCPHs imposes limitations on its widespread adoption and application in industrial settings. On the other hand, the stability and reliability of SCPHs wearable sensors need to be further improved. In practical scenarios, SCPHs may be susceptible to environmental conditions, such as temperature and humidity, resulting in water absorption and dehydration, causing fluctuations and instability in sensor performance. Promoting the industrialization of SCPHs is a challenging but essential task. Consequently, it is imperative to further optimize the preparation process and elevate the long‐term stability and reliability of SCPH sensors to facilitate the extensive utilization of SCPHs in industrial domains.

Fourthly, improving the integration and compatibility of other electronic devices for SCPH sensors is urgent. Existing SCPH sensor modules are relatively simple, poorly integrated, and monofunctional. In practice, SCPH wearable sensors usually need to be integrated with other sensors to realize more functions and application scenarios. However, the differences in operating principles, data formats, and interfaces between SCPH sensors and other electronic devices pose a challenge to integration and compatibility. Hence, it is necessary to focus on researching and improving the integration techniques of SCPH sensors to enhance their compatibility with other devices for meeting diversified application requirements.

Challenges and opportunities are intertwined. As an innovative sensing platform, the SCPH sensor exhibits a promising application outlook. By integrating supramolecular chemical bonding with CPHs, high‐performance and multifunctional sensors have been realized. These sensors hold immense potential for deployment in diverse domains, including motion sensing, sweat analysis, electrophysiological signal monitoring, and temperature sensing. It is foreseeable that the advent of novel wearable sensors based on SCPHs will profoundly enhance people's daily activities.

## CONFLICT OF INTEREST STATEMENT

The authors declare no conflicts of interest.
